# 4D-Flow Cardiovascular Magnetic Resonance Sequence for Aortic Assessment: Multi-Vendor and Multi-Magnetic Field Reproducibility in Healthy Volunteers

**DOI:** 10.3390/jcm12082960

**Published:** 2023-04-19

**Authors:** Bruna Punzo, Brigida Ranieri, Liberatore Tramontano, Ornella Affinito, Monica Franzese, Eduardo Bossone, Luca Saba, Carlo Cavaliere, Filippo Cademartiri

**Affiliations:** 1 IRCCS SYNLAB SDN, Via Emanuele Gianturco 113, 80143 Naples, Italy; bruna.punzo@synlab.it (B.P.); ranieribrigida@gmail.com (B.R.); liberatore.tramontano@synlab.it (L.T.); ornella.affinito@synlab.it (O.A.); monica.franzese@synlab.it (M.F.); 2 Department of Public Health, “Federico II” University of Naples, 80131 Naples, Italy; ebossone@hotmail.com; 3 Department of Radiology, Azienda Ospedaliero-Universitaria (A.O.U.) di Cagliari, 09123 Cagliari, Italy; lucasabamd@gmail.com; 4 Department of Radiology, Fondazione Monasterio/CNR, 56124 Pisa, Italy; filippocademartiri@gmail.com

**Keywords:** cardiac magnetic resonance, 4D-flow, aorta, phase-contrast CMR, aortic blood flow

## Abstract

Objectives: Four-dimensional (4D) flow cardiac magnetic resonance (CMR) represents an emerging technique for non-invasive evaluation of the aortic flow. The aim of this study was to investigate a 4D-flow CMR sequence for the assessment of thoracic aorta comparing different vendors and different magnetic fields of MR scanner in fifteen healthy volunteers. Methods: CMR was performed on three different MRI scanners: one at 1.5 T and two at 3 T. Flow parameters and planar wall shear stress (WSS) were extracted from six transversal planes along the full thoracic aorta by three operators. Inter-vendor comparability as well as scan–rescan, intra- and interobserver reproducibility were examined. Results: A high heterogeneity was found in the comparisons for each operator and for each scanner in the six transversal planes analysis (Friedman rank-sum test; *p*-value ≤ 0.05). Among all, the most reproducible measures were extracted for the sinotubular junction plane and for the flow parameters. Conclusions: Our results suggest that standardized procedures have to be defined to make more comparable and reproducible 4D-flow parameters and mainly, clinical impactfulness. Further studies on sequences development are needed to validate 4D-flow MRI assessment across vendors and magnetic fields also compared to a missing gold standard.

## 1. Introduction

The aorta is the main artery responsible for the oxygenated blood distribution to all parts of the body [1]. In the last decades, non-invasive imaging techniques (mostly Computed Tomography Angiography/CTA and Magnetic Resonance Angiography/MRA) have been developed to assess the morphology of the aorta without intravascular catheters [2,3]. Cardiac magnetic resonance (CMR) imaging is a non-invasive imaging modality with high spatial and temporal resolution, allowing a comprehensive assessment of the aorta without the use of ionizing radiation or the use of iodinated contrast agents. In recent years, the technological development of CMR has enabled performing functional evaluation of vessel flow and cardiovascular hemodynamics as part of conventional examination [4].

In the last decade, four-dimensional (4D) flow CMR has significantly improved from the technical and clinical standpoint becoming progressively more used in clinical routine. In the 2015, Dyverfeldt et al. published a consensus statement on 4D-flow CMR in clinical routine [5]. Therefore, 4D-flow CMR should be considered a comprehensive, non-invasive diagnostic approach able to quantify the blood flow in the main vessels of the chest. Several derived parameters have been investigated such as helical and vortical flow [6,7,8,9] as well as wall shear stress (WSS) [10,11,12,13] using the 4D-flow CMR technique [14].

Even though the technique is growing and improving, there are still several limitations in the widespread application of the 4D-flow CMR methodology and technique. One of the issues is related to reproducibility in different conditions and with different equipment (both vendor-wise and magnetic field-wise).

The aim of this study was to investigate the 4D-flow CMR sequence for the assessment of thoracic aorta (for evaluation of flow parameters and planar wall shear stress) comparing different vendors and different magnetic fields of MR scanner in healthy volunteers.

## 2. Material and Methods

### 2.1. Study Cohort

A total of 15 healthy volunteers (9 female and 6 male) were included in the study. All subjects had no aortic or cardiac pathology nor symptoms related to other diseases or co-morbidities.

All volunteers gave their written informed consent before participating in the study, and they underwent 4D-flow CMR based on an IRB-approved protocol (code 8/17 approved by Ethics Committee “Fondazione Pascale”). The study was approved by the Institutional Ethics Committee in accordance with the ethical standard of the Declaration of Helsinki.

### 2.2. CMR

CMR was performed on three different scanners: Philips digital 1.5 T (Achieva d-Stream 1.5 T MRI, Philips Healthcare, Best, The Netherlands), Philips digital 3 T (Achieva d-Stream 3 T MRI, Philips Healthcare, Best, The Netherlands) and Siemens 3 T (Biograph 3 T mMR, Siemens Healthineers, Forchheim, Germany). All subjects underwent CMR including, retrospectively, ECG gated time-resolved (CINE) balanced steady-state free precession (SSFP) imaging in four-chamber, two-chamber and short axis. Moreover, prospectively ECG gated time-resolved three-dimensional (3D) phase-contrast (PC) MR imaging with three-directional velocity encoding (4D-flow MR) was employed to measure in vivo 3D blood flow velocities in the whole aorta. The 4D-flow MR was acquired in a sagittal oblique 3D volume covering the entire thoracic aorta with prospective ECG gating and a respiratory navigator placed on the lung–liver interface. Further 4D-flow MR pulse sequence parameters were as follows: velocity encoding (VENC) 200 cm/s, field of view (FOV) 224 × 224 × 224 mm, scan matrix 160 × 160 × 160 (voxel size of 2.5 × 2.5 × 2.5 mm^3^), flip angle 8°, repetition time 4.35 ms and echo time 2.55 ms. No Compressed Sense was used. This sequence is still under development and not commercially available yet. The total acquisition time varied from 15 to 20 min depending on heart rate and navigator efficiency.

### 2.3. D-Flow Post-Processing

**Figure 1:**
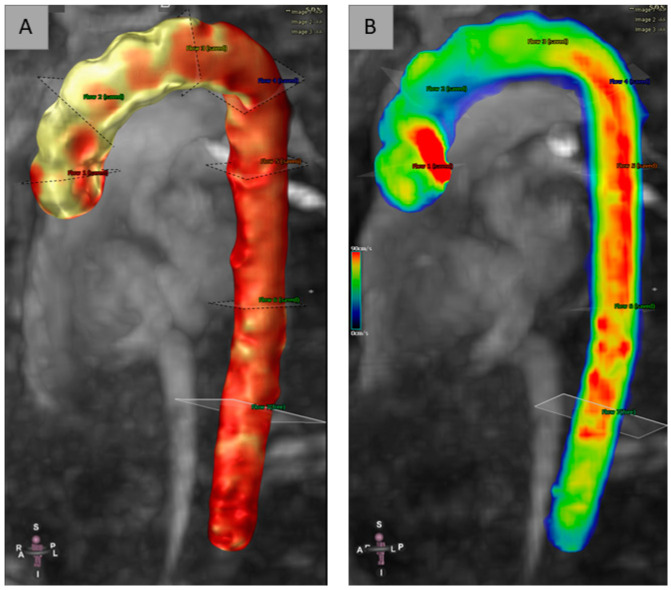
Segmentation with centerline: ROIs location in the full thoracic aorta (**A**); Velocity represented on colorimetric scale (**B**).

Six cross-sectional planes were equally distributed in the full thoracic aorta and were positioned along the centerline and perpendicular to the longitudinal axis of the aortic wall. Due to the variable shape and extend of the aorta, the three operators (OP1, OP2, OP3) have tried to place the six planes in the best standardized and reproducible way. Region of interest (ROI) 1 was positioned at the level of the sinotubular junction (above the bulb and aortic root). ROI_2 was equidistant positioned from ROI_1 and from the highest point of the aortic arch, proximal to the brachiocephalic trunk. ROI_3 was positioned on the highest point of the aortic arch, between the left common carotid artery and the left subclavian artery. The remaining ROIs (ROI_4, ROI_5 and ROI_6) were positioned along the descending aortic tract: ROI_4 at level of the at the aortic isthmus, ROI_5 in the descending aorta below the pulmonary artery, and ROI_6 was placed at level of the diaphragmatic aortic tract. All ROI locations are shown in Figure 1.

All data sets were analyzed using Circle Cardiovascular Imaging: CVI^42^ version 5.10.1 (Calgary, AB, Canada). A CVI^42^ 4D-flow tool was used for the flow assessment. The data were analyzed by three blinded expert operators (OP1, OP2, and OP3). The ascending aorta was contoured in the magnitude images with the sharpest blood/tissue contrast. Contours were propagated to phase contrast images in all temporal phases, which were corrected manually and controlled carefully. Phase-contrast MR images were evaluated for different parameters: flow parameters (peak velocity (PV), total volume (TV), maximum pressure gradient (MaxPressGrad), mean pressure gradient (MeanPressGrad), maximum flow (MaxFlow), maximum mean velocity (MaxMeanVel)), and planar Wall Shear Stress (WSS) parameters (axial WSS (AxWSS), circumferential WSS (CircWSS), maximum axial WSS (MaxAxWSS), maximum circumferential WSS (MaxCircWSS)) and were extracted from six transversal planes along the aorta.

Moreover, 4D-flow gives capabilities for large blood flow assessment via blood flow visualization using color-coded 3D multiplanar reformations, streamlines, and velocity vectors.

As with eco color-doppler US, adding color coding allows a visualization of low and high velocities within the volume at a glance. In general, red is used for high velocities and blue is used for low velocities.

All data extracted from the processing were evaluated, and inter- and intra-observer analysis was performed from three different researchers to measure data reproducibility.

### 2.4. Power Analysis

A priori power analysis was performed considering a *repeated*-*measures design* in which multiple measurements by three different radiological operators are made on each MRI scanner (Philips 1.5 T, Philips 3 T, Siemens 3 T). The minimum sample size was computed using the ANOVA test for repeated measures given a power of 0.85, a small effect size (f = 0.25) and an alpha level of 0.05. Based on previous assumptions, the required total sample size was 39: 13 for each MRI scanner.

### 2.5. Data Analysis

All statistical analysis was performed using R Statistical Software (version 4.1.0; R Foundation for Statistical Computing, Vienna, Austria) after the removal of outliers. Descriptive analysis was used to describe the basic features of the data. Descriptive data are presented as mean and standard deviation (SD), coefficient variation (CV) and 95% confidence intervals (CI). Association among parameters was assessed by Spearman’s rank correlation, and it was calculated on ROIs average. The Shapiro–Wilk test was used to assess the normality of the data. Alpha was set at *p* ≤ 0.05. *p*-values are adjusted using the Bonferroni multiple testing correction method.

Agreement in the test results repeatedly by the same operator (intra-rater reliability), by three different operators (inter-operator agreement) and by three different devices (inter-vendor reliability) was determined. Comparison among different ROIs (intra-rater reliability) was assessed by Friedman rank-sum test.

The inter-operator agreement was assessed by Lin’s concordance correlation coefficient (CCC) [15]. The Bland–Altman plot was used to measure the limits of agreement between the measurements of the two operators and to evaluate the ROI_1 reproducibility. The limits of agreement equaling two SD of the mean difference above and below the mean were plotted [16].

The inter-device agreement was assessed by a Kruskal–Wallis rank-sum test followed by post hoc analysis (Wilcoxon signed-rank test).

**Figure 2:**
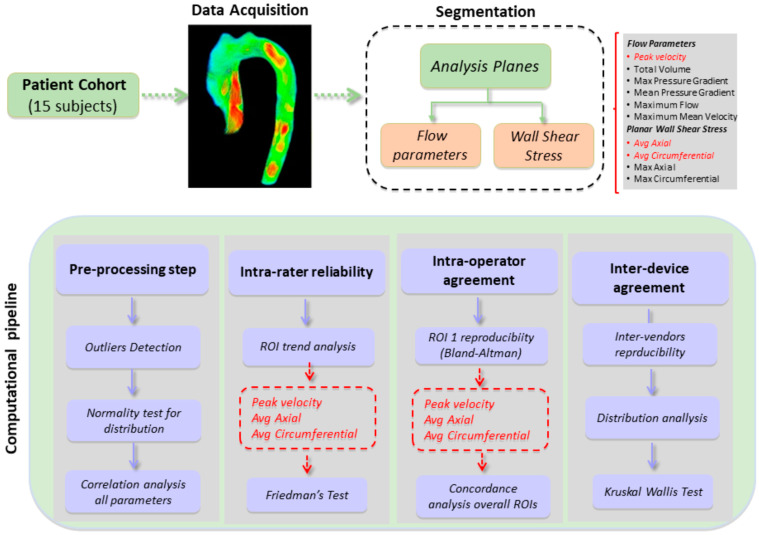
Workflow.

The methodological workflow is reported in Figure 2.

## 3. Results

The population demographics and characteristics of 15 healthy volunteers are reported in Supplementary Table S1.

### 3.1. Intra-Rater Reliability

The intra-rater reliability results are summarized in Supplementary Tables S2–S4.

**Figure 3:**
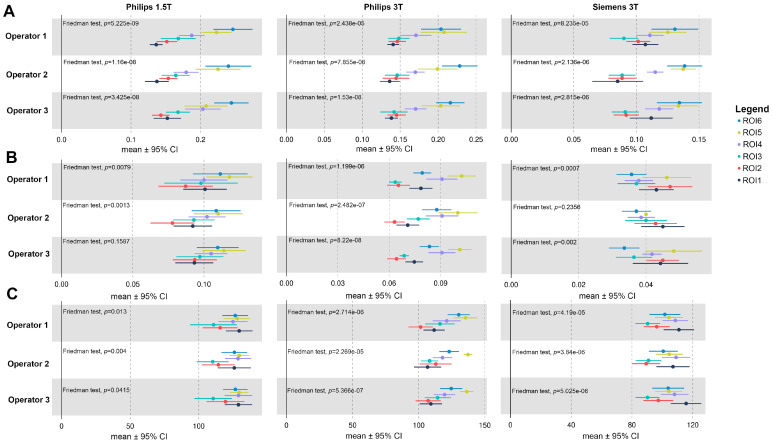
Forest plot of the mean with 95% CI obtained from each ROI and from the three devices (Philips 1.5 T, Philips 3 T and Siemens 3 T) for the (**A**) AxialWSS, (**B**) CircWSS, (**C**) Flow parameters. Horizontal lines through the circle represent 95% confidence intervals. The circle represents the mean.

Figure 3 shows a forest plot of the mean with the corresponding 95% CI obtained from each ROI for AxWSS (A), CircWSS (B) and Flow (C) parameters. Regardless of operator, parameters and device, all ROIs show statistically significant differences (Friedman rank-sum test; *p*-value ≤ 0.05). Only the parameter CircWSS shows any significant difference when measured by OP3 on a Philips 1.5 T device and OP2 on a Siemens 3 T device.

### 3.2. ROI_1 Reproducibility

The previous analysis showed a high variability between the different ROIs selected. This may depend on the different anatomical positions of planes selected along the aorta. The anatomical variability did not allow establishing a standard distance for each examined ROI. One of the planes that is least affected by this variability is the ROI_1 positioned at the aortic root. We therefore went to investigate how much these measurements in ROI_1 alone was reproducible among themselves.

**Figure 4:**
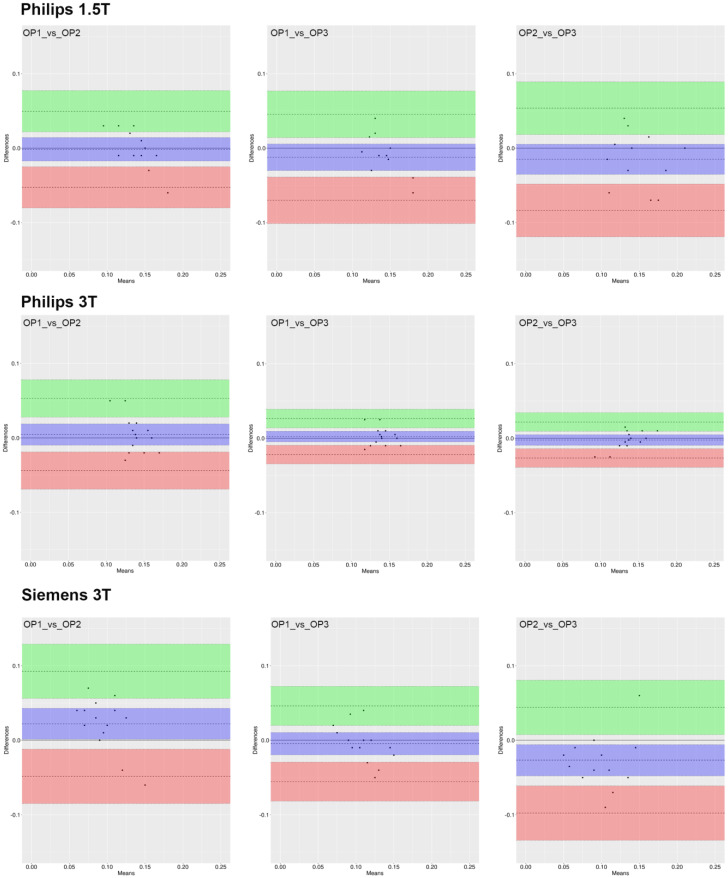
Bland–Altman plot of AxialWSS measures for each pair of raters. The dashed line represents the mean difference along with the 95% CI (violet box). The lower (red box) and higher (green box) limits of agreement (LoA) defined as the mean difference ± 1.96 standard deviations of differences are reported along with the corresponding 95% CI.

**Figure 5:**
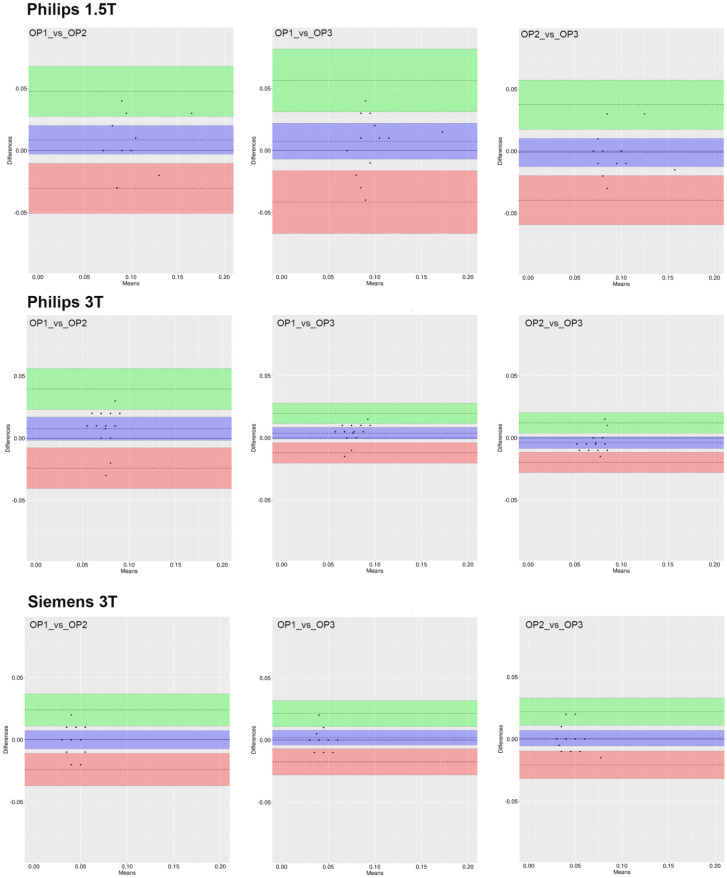
Bland–Altman plot of CircWSS measures for each pair of raters. The dashed line represents the mean difference along with the 95% CI (violet box). The lower (red box) and higher (green box) limits of agreement (LoA) defined as the mean difference ± 1.96 standard deviations of differences are reported along with the corresponding 95% CI.

**Figure 6:**
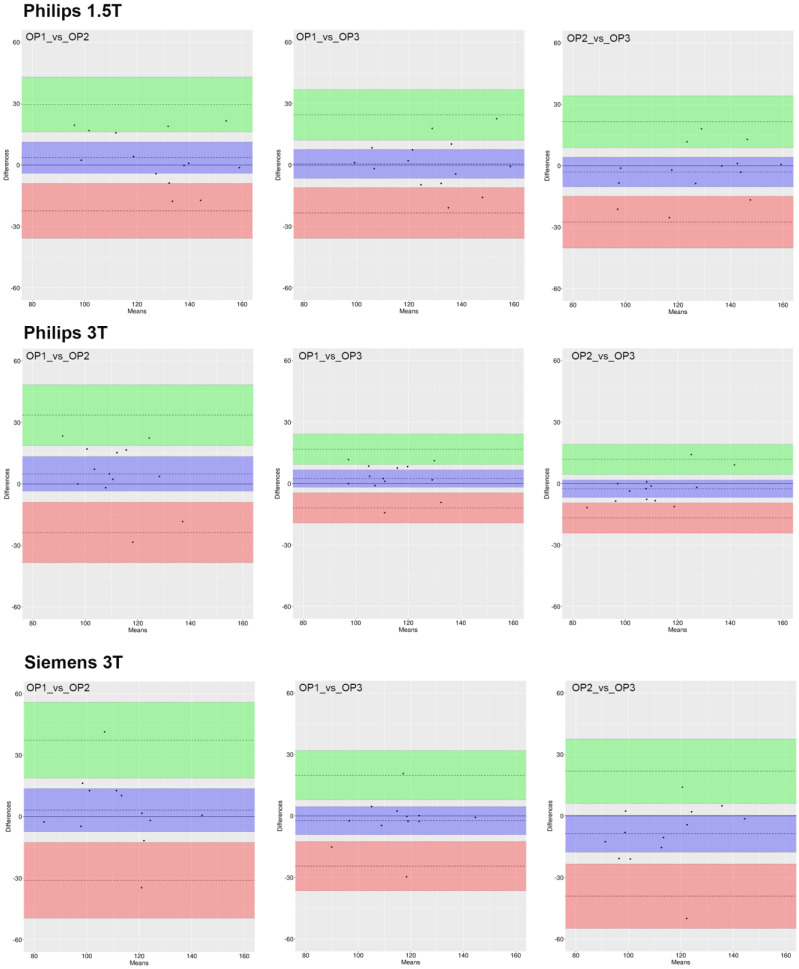
Bland–Altman plot of flow measures for each pair of raters. The dashed line represents the mean difference along with the 95% CI (violet box). The lower (red box) and higher (green box) limits of agreement (LoA) defined as the mean difference ± 1.96 standard deviations of differences are reported along with the corresponding 95% CI.

Results from the analysis of the Bland–Altman plots (Figure 4, Figure 5 and Figure 6) demonstrate that 95% of all measurement differences lay within the statistical LoA (±2SD), indicating that the ROI_1 reproducibility among three investigators was acceptable.

### 3.3. Pre-Processing Data

A preliminary correlation analysis was performed to evaluate the adding value of other extracted 4D-flow parameters. The results showed that for each device, these parameters did not have a high correlation between them in all operators (Supplementary Figure S1); thus, we considered all extracted parameters for further inter-operator and inter-device agreement analysis. Summary statistics for 4D-flow parameters are reported in Supplementary Table S5.

### 3.4. Inter-Operator Agreement

The CCC was used to assess the agreement between operators. CCC values are reported in Supplementary Table S6 along with the corresponding 95% confidence interval (CI). Most of the CCC values were higher than 0.7, implying a good agreement. Only the parameter CircWSS measured on the devices Philips 3 T and Siemens 3 T experienced CCC values (CCC = 0.45, 95% CI = −0.071–0.784, and CCC = 0.61, 95% CI = 0.145–0.852, respectively) corresponding to moderate agreement between the OP1 and OP2.

Results from the analysis of the Bland–Altman plots (Supplementary Figure S2) demonstrate that 95% of all measurement differences lay within the statistical LoA (±2SD), indicating that the agreement between measurements taken by the three investigators was acceptable.

### 3.5. Inter-Device Agreement

**Figure 7:**
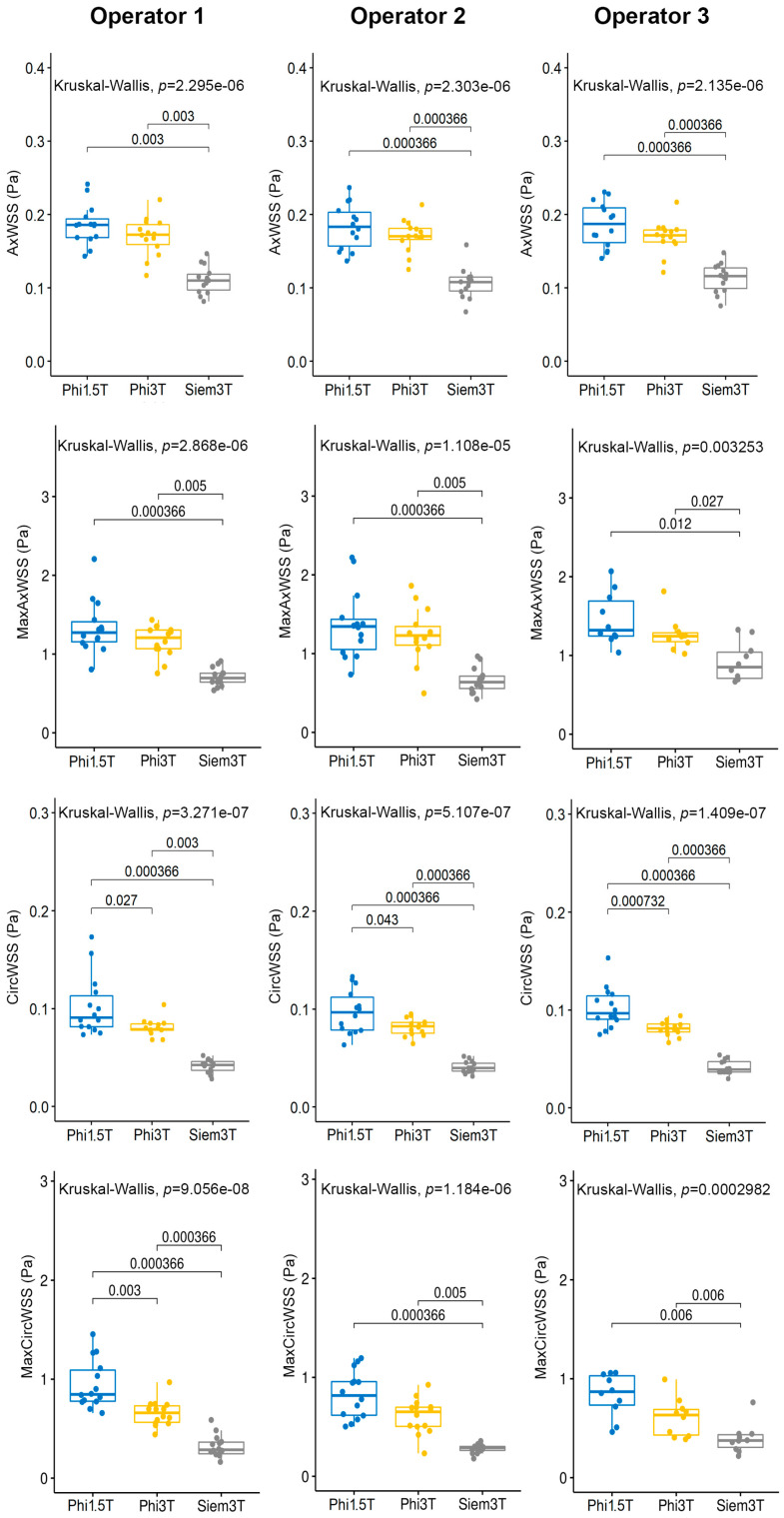
Inter-device agreement of AxWSS, MaxAxWSS, CircWSS and MaxCircWSS. For each parameter (AxWSS, MaxAxWSS, CircWSS, MaxCircWSS), boxplots of Kruskal–Wallis test show the comparison of measurements from 3 devices (Philips 1.5 T, Philips 3 T, and Siemens 3 T) using data by operator 1, operator 2 and operator 3.

Overall, for most of the parameters, the Siemens 3T measurements are the lowest, followed by Philips 3 T and Philips 1.5 T, except for MaxMeanVel measured by OP3, MaxFlow and TV (Supplementary Table S5; Figure 7; Supplementary Figures S3 and S4). In the latter cases, the ANOVA analysis is not significant. All the other parameters show statistically significant differences among the devices (Kruskal–Wallis rank-sum test; *p*-value ≤ 0.05). The most significant differences are between the two Philips devices and Siemens 3 T (post hoc analysis; Wilcoxon signed rank test, *p*-value ≤ 0.05).

Statistically significant differences among the three devices were also found for the CircWSS parameter regardless of operators, for the MaxCircWSS-AllPhases parameter only for the OP1 shown in Figure 7 and for the MeanPressGrad parameter for OP2 and OP3 (Supplementary Figure S4) (post hoc analysis; Wilcoxon signed-rank test, *p*-value ≤ 0.05).

Moreover, to evaluate the inter-device reproducibility, we estimated the coefficient of variation (CV) (Supplementary Table S5). Regardless of operator, on the Philips 3 T, the lowest CV was observed for the parameters CircWSS (meanCV = 10.2%), Flow (meanCV = 12.3%) and MeanPressGrad (meanCV = 24.0%). On the Philips 1.5 T, the lowest CV was observed for the parameters MaxFlow (meanCV = 19.2%) and TotalVolume (meanCV = 16.7%). Other parameters (AxWSS, MaxAxialWSS-AllPhases, MaxMeanVel and MaxPressGrad) show the lowest CV on the same device for only two raters. Only the parameter MaxCircWSS-AllPhases shows the lowest CV on different devices depending on the rater (Supplementary Table S5).

## 4. Discussion

Time-resolved 3D PC MRI sequences, named 4D-flow sequence, represent an emerging technique for non-invasive evaluation of the aortic flow without contrast agent injection [17]. This technique can be used in both healthy volunteers, in order to validate its applicability, as well as in patients affected by aortic pathologies. In the literature, 4D-flow can be used to assess the adverse hemodynamic consequences of bicuspid aortic valve (BAV), such as altered distribution of aortic blood flow helicity, vorticity, and eccentricity. Some studies compared the blood flow pattern and WSS map in healthy individuals and patients with BAV, and they found that the presence of a BAV alters the flow pattern and WSS distribution in the ascending aorta [18,19].

This technique gives new insights into physiological and pathophysiological flow patterns not currently observable with conventional two-dimensional (2D) flow sequences [20].

This study is a single-center experience that investigates 4D-flow multi-vendor and multi-magnetic field reproducibility. It is important to note that nowadays, the sequence is under investigation and not routinely employed for clinical use. Moreover, the absence of reference standards and the absence of normal ranges for the parameters evaluated limit any conclusion about the best MRI systems for 4D-Flow assessment. For this reason, in our study, a preliminary setting of the technical parameters was performed.

The sequence parameters were evaluated for each scanner, and the discrepancies were modified to make the sequence as reproducible as possible. FOV, slice thickness, flip angle, VENC, etc. were modified accordingly (see Materials and Methods).

We use three different machines for both the vendor and magnetic field, as previously described, and three different operators evaluated the following parameters: PV, TV, MaxPressGrad, MeanPressGrad, MaxFlow, MaxMeanVel, AxWSS, CircWSS, MaxAxWSS, and MaxCircWSS.

Usually, the quantitative analysis of CMR images is based on manual contouring or manual correction of semi-automatic segmented ROI in CMR images.

A high heterogeneity was found in the comparisons for each operator and for each machine in the six ROIs analysis. All the measures were statistically significant and therefore different from each other.

According to our experience, the low reproducibility of the measurements could depend on anatomical aortic variations (aorta length, diameter) such as intra-operator variability given the difficulty of establishing a standard and reproducible distance along the entire thoracic aortic axis. In fact, intra-rater reliability analysis showed statistically significant differences among all ROIs regardless of operator, parameters, and device. This suggests that the variability observed in the measurements may be due to differences in the anatomical positions of the planes selected along the aorta. However, the ROI_1 (at the level of sinotubular junction) has a higher degree of reproducibility. This is almost certainly due to the possibility of a fixed and reproducible anatomical landmark such as the aortic root. Regardless of the equipment, the agreement overall between the operators is fair/moderate. For both AxWSS, CircWSS and Flow (PV), there is good concordance between all three operators. This is an important finding, as it suggests that measurements taken at this specific location may be more reliable than those taken at other locations along the aorta.

In this study, 10 different parameters were evaluated with CCC. Inter-operator agreement analysis showed that most of the CCC values were higher than 0.7, indicating good agreement between the operators. However, there were some cases where the CCC values were lower, suggesting only moderate agreement between the operators. This was particularly true for the parameter CircWSS measured on the Philips 3 T and Siemens 3 T devices. Nonetheless, the Bland–Altman plots demonstrated that the agreement between measurements taken by the three investigators was acceptable. Therefore, it would be extremely useful to seek a standard method (as uniform as possible) in order to minimize operator-dependent variability.

Instead, significant differences were found between the different vendor scanners: the inter-device agreement analysis showed that there were statistically significant differences among the devices for most of the parameters except for MaxMeanVel measured by OP3, MaxFlow, and TV. The Siemens 3T measurements were consistently the lowest, which were followed by Philips 3 T and Philips 1.5 T. The most significant differences were observed between the two Philips devices and Siemens 3 T. This suggests that the choice of device may have an impact on the measurements obtained.

In the recent literature, most of the validation studies have been limited to one vendor platform or multicenter enrollment that can influence the acquisition data. Thus, there is a need to demonstrate how 4D-flow performs on scanners from different vendors [21].

Finally, the coefficient of variation (CV) was calculated to evaluate the inter-device reproducibility. The results showed that the lowest CV was observed for different parameters depending on the device and the rater. Nonetheless, the parameters with the lowest CV were CircWSS, Flow, and MeanPressGrad on Philips 3 T, and MaxFlow and TotalVolume on Philips 1.5 T. These findings suggest that certain parameters may be more reliable than others, depending on the device and the rater.

Overall, these results highlight the importance of carefully selecting the location of the ROI as well as the device used for measurements. Additionally, it is crucial to ensure that the measurements are taken by well-trained operators who can achieve a high level of agreement between themselves.

Further studies of sequences investigation across vendors and different magnetic field are needed to standardize 4D-flow protocols across vendors and magnetic fields. In particular, we will deepen the study both on healthy subjects and on pathological subjects.

The efficacy of non-invasive 4D-flow CMR protocol could shed light on how to standardize the measures assessment obtaining hemodynamic details, improving the CMR information.

## 5. Limitations

In this study, only a small number of healthy volunteers were enrolled. Moreover, the age range of our population was restricted to 26–49 years old, and elderly patients were not included.

Nowadays, each vendor used its recommended protocol for optimal acquisition. Nevertheless, the adjustment of acquisition parameters has been applied to optimize the 4D-flow sequence on three different machines, and this could represent a potential source of error. Moreover, the lack of an evaluation on intra-device agreement (i.e., same patient re-examined in the same scan system) represented the main limitation of the present study.

## 6. Conclusions

The 4D-flow CMR provided valuable information on hemodynamic parameters. Its clinical utility has been especially useful in assessing flow patterns through the heart and great vessels [22]. However, aortic hemodynamic parameters assessed with various vendor-provided protocols obtained with 4D-flow CMR are not equivalent in a population of healthy volunteers. Overall, the plane positioned at the ROI_1 showed the most inter-vendor stability and agreement. Probably, this is due to the accurate ROI_1 placement thanks to the aortic root reference point. A standardized anatomical reference point in the setting of ROIs during post-processing analysis could be useful for parameters’ reproducibility.

The artificial intelligence application could help to better identify this type of “marker” with the aim of decreasing the variability of the measurements.

Regarding the absence of existing commercial 4D-flow MR sequence for clinical use, it would be suggested that the follow-up examination should occur at the same scanner. In this way, the lack of potential confounders should be reduced [23].

## Data Availability

The data presented in this study are available on request from the corresponding author.

## Supplementary Materials

The following supporting information can be downloaded at: https://www.mdpi.com/article/10.3390/jcm12082960/s1.
