# Supplementary material for: 4D-Flow Cardiovascular Magnetic Resonance Sequence for Aortic Assessment: Multi-Vendor and Multi-Magnetic Field Reproducibility in Healthy Volunteers
Source: J Clin Med. 2023 Apr 19;12(8):2960. doi: 10.3390/jcm12082960 (PMC10141060; doi:10.3390/jcm12082960)
Supplement: Supplementary file 1 [file jcm-12-02960-s001.zip › jcm-2303694- supplementary.pdf]

Supplementary Material

4D-flow Cardiovascular Magnetic Resonance sequence for aortic assessment: multi-vendor and multi-magnetic field reproducibility in healthy volunteers

Bruna Punzo[1†], Brigida Ranieri [1†], Liberatore[1], Tramontano, Ornella Affinito[1], Monica Franzese[1], Eduardo Bossone[2], Luca Saba[3], Carlo Cavaliere[1*], Filippo Cademartiri[4].

*** Correspondence:** carlo.cavaliere@synlab.it

1 Supplementary Tables

Supplementary Table S1_Population demographics and characteristics.

Variables	Mean ± SD (range)	Philips 1.5 T	Philips 3.0 T	Siemens 3.0 T
Gender (male/female)	6/9	-	-	-
Age (years)	33.71 ± 6.58	-	-	-
Height (cm)	171.07 ± 8.09	-	-	-
Weight (Kg)	70.21 ± 14.40	-	-	-
BMI	23.76 ± 3.01	-	-	-
BSA (m^2)	1.82 ± 0.22	-	-	-
Heart rate (bpm)	66.5 ± 6.12	69.14±8.87	66.86±8.99	63.50±6.05
Ejection fraction (%)	62.8 ± 5.25	62.93±4.55	62.87±5.24	62.61±6.14
LV-EF (%)	63.87 ± 8.47	69.06±5.65	65.25±6.64	60.01±8.36

RV-EF (%)	54.75 ± 7.64	55.71±6.90	55.63±6.58	54.96±7.62
LV-EDV/BSA (ml/m^2)	75.94 ± 14.53	74.10±10.47	74.68±16.78	75.55±12.80
LV-ESV/BSA (ml/m^2)	27.85 ± 10.13	23.27±6.87	26.37±10.32	30.95±11.08
LV-SV/BSA (ml/m^2)	48.08 ± 9.49	50.83±5.25	48.30±9.93	44.60±5.15
RV-EDV/BSA (ml/m^2)	83.24 ± 22.41	76.62±13.55	83.40±18.08	80.09±17.11
RV-ESV/BSA (ml/m^2)	38.54 ± 14.22	33.88±7.57	37.56±11.84	36.77±12.09
RV-SV/BSA (ml/m^2)	44.34 ± 9.75	42.74±9.84	45.83±8.41	43.02±7.24
LV Mass (g/m^2)	55.89 ± 9.08	56.38±8.02	56.64±9.55	53.85±8.66

BMI, Body Mass Index; BSA, Body Surface Area; LV-EF, Left Ventricle Ejection Fraction; RV-EF, Right Ventricle Ejection Fraction; LV-EDV, Left Ventricle Endo Diastolic Volume; LV-ESV, Left Ventricle Endo Systolic Volume; LV-SV, Left Ventricle Stoke Volume; RV-EDV, Right Ventricle Endo Diastolic Volume; RV-ESV, Right Ventricle Endo Systolic Volume; RV-SV, Right Ventricle Stoke Volume; LV Mass, Left Ventricle Mass.

Supplementary Table S2_Inter-variabilityPhilips 3T.

Philips 3T device	ROI 1			ROI 2			ROI 3			ROI 4			ROI 5			ROI 6		
4DFlow parameters	*mean±sd*	*CV*	*95%CI*	*mean±sd*	*CV*	*95%CI*	*mean±sd*	*CV*	*95%CI*	*mean±sd*	*CV*	*95%CI*	*mean±sd*	*CV*	*95%CI*	*mean±sd*	*CV*	*95%CI*
**OPERATOR 1**																		
AxialWSS	0,14±0,01	10,6	0,01	0,15±0,02	15,1	0,01	0,15±0,03	17,4	0,02	0,17±0,04	23	0,02	0,21±0,06	28,2	0,03	0,20±0,05	24,7	0,03
CircWSS	0,08±0,01	16,5	0,01	0,07±0,01	19,5	0,01	0,06±0,01	10,1	0,004	0,09±0,02	19,1	0,01	0,10±0,02	15,4	0,01	0,08±0,04	12,6	0,01
Flow	111,7±15,4	13,8	8,9	101,4±17,6	17,4	10,2	116,1±21,3	18,3	12,3	121,2±20,4	16,6	11,6	135,4±15,9	11,7	10,1	130,3±15,4	11,9	9,3
**OPERATOR 2**																		
AxialWSS	0,14±0,02	18,3	0,01	0,14±0,03	23,3	0,02	0,15±0,03	21,1	0,02	0,17±0,02	13,6	0,01	0,20±0,05	24,9	0,03	0,23±0,04	19,6	0,03

CircWSS	0,07±0,01	18	0,01	0,06±0,01	18	0,01	0,08±0,01	16,4	0,01	0,09±0,02	20, 0	0,01	0,1±0,02	21,9	0,01	0,09±0,02	18,5	0,01
Flow	106,7±19,7	18,4	11,4	112,9±23,0	20,4	13,3	108,2±12,0	11,1	7,3	118±18,1	12,0	8,2	137,3±5,7	4,2	3,6	123,0±14,1	11,5	8,2
	**OPERATOR 3**																	
AxialWSS	0,14±0,02	11,9	0,01	0,15±0,02	16,0	0,01	0,14±0,03	24,2	0,02	0,17±0,03	16,2	0,02	0,20±0,05	24,0	0,03	0,22±0,04	16,5	0,02
CircWSS	0,07±0,01	13,3	0,01	0,06±0,01	16,4	0,01	0,07±0,005	7,01	0,003	0,09±0,02	16,6	0,01	0,10±0,01	12,9	0,01	0,08±0,01	13,0	0,01
Flow	109,2±16,1	14,7	9,3	107,2±18,3	17,1	10,6	114,4±18,9	16,5	11	119,6±15,5	12,9	8,9	136,4±9,1	6,7	5,8	124,6±16,1	12,9	9,3

Supplementary Table S3_Inter-variabilitySiemens 3T.

**Siemens 3T device**	**ROI 1**			**ROI 2**			**ROI 3**			**ROI 4**			**ROI 5**			**ROI 6**		
4DFlow parameters	***mean±sd***	***CV***	***95%CI***	***mean±sd***	***CV***	***95%CI***	***mean±sd***	***CV***	***95%CI***	***mean±sd***	***CV***	***95%CI***	***mean±sd***	***CV***	***95%CI***	***mean±sd***	***CV***	***95%CI***
**OPERATOR 1**																		
AxialWSS	0,11±0,02	18,5	0,02	0,10±0,02	18,1	0,01	0,09±0,02	18,1	0,01	0,11±0,02	18,9	0,01	0,13±0,03	22,8	0,02	0,13±0,04	27,2	0,02
CircWSS	0,04±0,01	22,0	0,01	0,05±0,01	26,9	0,01	0,04±0,01	28,8	0,01	0,04±0,01	21,2	0,004	0,05±0,01	28,7	0,01	0,04±0,01	23,8	0,005
Flow	110,9±18,4	16,6	11,1	96,3±16,3	17,0	9,4	90,3±15,6	17,2	9,0	108,6±16	14,8	9,2	104,4±17,4	16,7	10,1	101,6±19,5	19,2	11,2
**OPERATOR 2**																		
AxialWSS	0,09±0,04	45,6	0,02	0,09±0,02	23,8	0,01	0,09±0,02	22,9	0,01	0,12±0,01	10,2	0,01	0,14±0,02	12,2	0,01	0,14±0,03	18,6	0,02
CircWSS	0,05±0,01	27,2	0,01	0,04±0,01	28,1	0,01	0,04±0,01	29,4	0,01	0,04±0,01	20, 0	0,004	0, 04±0	0	0	0,04±0,01	22,2	0,005
	107,0±21,0	19,5	12,1	89,4±17,0	19,1	10,3	90,9±16,0	17,6	9,3	109±18,0	16,4	10,3	104,5±17	16,2	9,8	100,6±18,1	18,0	10,5

Flow																		
	**OPERATOR 3**																	
AxialWSS	0,11±0,03	29,3	0,02	0,09±0,02	20,9	0,01	0,09±0,02	22,5	0,01	0,12±0,02	18,2	0,01	0,13±0,03	22,4	0,02	0,13±0,03	25,5	0,02
CircWSS	0,04±0,02	35,4	0,01	0,05±0,01	20,4	0,01	0,04±0,01	28,2	0,01	0,04±0,005	12,9	0,003	0,05±0,02	33,4	0,01	0,03±0,01	25,7	0,005
Flow	115,6±19,1	16,5	11,0	97,3±18,7	19,2	10,8	90,2±14,9	16,5	8,6	108,0±17,3	16,0	10,0	104,2±17,0	16,3	9,8	103,8±20,0	19,3	11,6

Supplementary Table S4_Inter-variabilityPhilips 1.5T.

**Philips 1.5T device**	**ROI 1**			**ROI 2**			**ROI 3**			**ROI 4**			**ROI 5**			**ROI 6**		
4DFlow parameters	***mean±sd***	***CV***	***95%CI***	***mean±sd***	***CV***	***95%CI***	***mean±sd***	***CV***	***95%CI***	***mean±sd***	***CV***	***95%CI***	***mean±sd***	***CV***	***95%CI***	***mean±sd***	***CV***	***95%CI***
									**OPERATOR 1**									
AxialWSS	0,14±0,02	12,6	0,01	0,15±0,03	18,8	0,02	0,17±0,05	28,5	0,03	0,19±0,04	19	0,02	0,22±0,04	18,3	0,02	0,25±0,05	21,9	0,03
CircWSS	0,10±0,03	29	0,02	0,09±0,04	41,8	0,02	0,10±0,05	50,4	0,03	0,10±0,03	31,6	0,02	0,12±0,03	26,2	0,02	0,11±0,04	32,8	0,02
Flow	129,4±18,5	14,3	10,7	115,6±24,3	21,1	14,0	110,8±32,5	29,3	18,7	124,8±20,4	21,1	11,7	128±18,1	14,2	10,5	126,5±18	14,2	10,4
									**OPERATOR 2**									
AxialWSS	0,14±0,03	23,7	0,02	0,15±0,02	16,1	0,01	0,16±0,04	23,5	0,02	0,18±0,03	19,3	0,02	0,22±0,06	27,4	0,04	0,24±0,06	26,3	0,04
	0,09±0,03	28	0,01	0,08±0,03	37,8	0,02	0,09±0,03	30	0,02	0,10±0,02	24, 3	0,01	0,11±0,03	30	0,02	0,11±0,03	30,2	0,02

CircWSS	125,8±23,0	18,3	13,3	114,2±22,8	19,6	13,2	110,1±22,2	20,2	12,8	128,5±18,1	14,1	10,4	129,5±13,3	10,3	8,1	125,9±17,9	14,2	10,3
Flow																		
									**OPERATOR 3**									
AxialWSS	0,15±0,04	24,8	0,02	0,14±0,02	17,2	0,01	0,17±0,03	19,7	0,02	0,20±0,05	24,0	0,03	0,21±0,06	28,4	0,03	0,24±0,05	19,3	0,03
CircWSS	0,10±0,03	27,2	0,01	0,10±0,03	29	0,02	0,10±0,03	29	0,02	0,10±0,03	29	0,02	0,10±0,03	29	0,02	0,10±0,03	29	0,02
Flow	129,4±18,5	14,3	10,7	129,4±18,5	14,3	10,7	129,4±18,5	14,3	10,7	129,4±18,5	14,3	10,7	129,4±18,5	14,3	10,7	129,4±18,5	14,3	10,7

Supplementary Table S5_Inter-operator & inter-device.

4DFlow parameters	OPERATOR 1		OPERATOR 2		OPERATOR 3	
**AxialWSS**	***mean±sd***	***CV***	***mean±sd***	***CV***	***mean±sd***	***CV***
Philips 1.5 T	0,19±0,03	14,8	0,18±0,03	16,7	0,19±0,03	16,5
Philips 3 T	0,17±0,03	15,4	0,17±0,03	12,9	0,17±0,03	13,1
Siemens 3 T	0,11±0,02	16,7	0,11±0,02	19,7	0,11±0,02	17,2
**MaxAxialWSS**	***mean±sd***	***CV***	***mean±sd***	***CV***	***mean±sd***	***CV***
Philips 1.5 T	1,39±0,36	25,6	1,48±0,44	29,6	1,46±0,33	22,7
Philips 3 T	1, 17±0,20	17,2	1,24±0,34	27,8	1, 18±0,33	27,6
Siemens 3 T	0,72±0,10	14,5	0,71±0,15	20,4	0,92±0,24	26,7
**CircWSS**	***mean±sd***	***CV***	***mean±sd***	***CV***	***mean±sd***	***CV***
Philips 1.5 T	0,10±0,03	29,9	0,10±0,02	23,0	0,10±0,02	20,6
Philips 3 T	0,08±0,01	10,8	0,08±0,01	10,7	0,08±0,01	9,0
Siemens 3 T	0,04±0,01	16,2	0,04±0,01	14,7	0,04±0,01	17,2
**MaxCircumferentialWSS**	***mean±sd***	***CV***	***mean±sd***	***CV***	***mean±sd***	***CV***
Philips 1.5 T	0,92±0,25	27,4	0,86±0,26	30,5	0,84±0,22	26,2
Philips 3 T	0,66±0,13	19,6	0,61±0,18	29,5	0,59±0,22	36,8
Siemens 3 T	0,27±0,07	24,5	0,30±0,03	9,5	0,40±0,15	37,8
**Flow**	***mean±sd***	***CV***	***mean±sd***	***CV***	***mean±sd***	***CV***
Philips 1.5 T	122,5±17,7	14,4	121,8±17,2	14,2	123,4±16,9	13,7
Philips 3 T	117,7±15,5	13,2	116,4±13,9	12,0	117,6±14,0	11,9
Siemens 3 T	101,8±14,9	14,6	100,7±15,6	15,5	103,2±15,2	14,7

**MaximumFlow**	***mean±sd***	***CV***	***mean±sd***	***CV***	***mean±sd***	***CV***
Philips 1.5 T	234,1±40,7	17,4	234,0±36,1	15,4	228,5±40,4	17,7
Philips 3 T	255,0±65,8	25,8	247,4±63,6	25,8	241,4±60,5	25,1
Siemens 3 T	237,3±49,4	20,8	242,2±57,2	23,6	234,1±46,5	19,9
**MaximumMeanVelocity**	***mean±sd***	***CV***	***mean±sd***	***CV***	***mean±sd***	***CV***
Philips 1.5 T	78,0±9,0	11,5	79,2±13,7	17,3	77,7±10,4	13,4
Philips 3 T	65,6±13,6	20,7	66,6±9,4	14,1	63,8±10,3	16,2
Siemens 3 T	65,7±10,9	16,6	65,5±10,9	16,6	69,3±11,4	16,4
**MaxPressureGradient**	***mean±sd***	***CV***	***mean±sd***	***CV***	***mean±sd***	***CV***
Philips 1.5 T	5,8±1,3	22,1	5,9±1,4	23,0	6,0±1,2	19,2
Philips 3 T	5,3±1,3	25,9	4,8±1,3	27,0	4,4±1,1	25,4
Siemens 3 T	4,24±1,33	31,4	4,20±1,22	29,1	4,36±1,27	29,2
**MeanPressureGradient**	***mean±sd***	***CV***	***mean±sd***	***CV***	***mean±sd***	***CV***
Philips 1.5 T	1,36±0,32	23,8	1,32±0,32	23,9	1,37±0,25	18,5
Philips 3 T	1,07±0,3	25,5	1,01±0,3	28,7	1,00±0,3	30,2
Siemens 3 T	0,77±0,23	29,7	0,77±0,22	29,0	0,82±0,24	29,9
**TotalVolume**	***mean±sd***	***CV***	***mean±sd***	***CV***	***mean±sd***	***CV***
Philips 1.5 T	52,9±9,0	17,0	53,5±8,2	15,4	53,1±7,7	14,5
Philips 3 T	58,3±11,5	19,7	56,00±12,4	22,1	52,1±14,5	27,8
Siemens 3 T	53,7±8,95	16,7	55,5±10,9	19,6	53,3±8,4	15,8

Supplementary Table S6_CCC inter-operator agreement.

	Inter – operator agreement					
Philips 1.5 T	***operator 1 - operator 2***		***operator 1 - operator 3***		***operator 2 - operator 3***	
**4DFlow parameters**	**CCC**	**95%CI**	**CCC**	**95%CI**	**CCC**	**95%CI**
AxialWSS	0,84	0,58- 0,94	0,77	0,44- 0,91	0,79	0,48- 0,93
MaxAxialWSS	0,10	0 - 0,19	0,06	-0,02- 0,13	0,67	0,16- 0,90
CircWSS	0,002	0 - 0,003	0,002	0 - 0,003	-0,014	-0,08- 0,06
MaxCircumferentialWSS	0, 08	-0,01- 0,17	-0, 01	-0,08- 0,06	-0, 05	0,62- 0,56
Flow	0,92	0,78- 0,97	0,94	0,82- 0,98	0,95	0,86- 0,99
MaximumFlow	0	0 - 0	0	0 - 0	0,71	0,20- 0,92
MaximumMeanVelocity	0,84	0,63- 0,93	0,90	0,66- 0,97	0,91	0,77- 0,97
MaxPressureGradient	0,93	0,75- 0,98	0,91	0,71- 0,98	0,95	0,84- 0,98
MeanPressureGradient	0,88	0,60- 0,97	0,86	0,59- 0,96	0,94	0,84- 0,98
TotalVolume	0,838	0,58- 0,94	0,86	0,6663- 0,97	0,82	0,43- 0,95
Philips 3T	***operator 1 - operator 2***		***operator 1 - operator 3***		***operator 2 - operator 3***	
**4DFlow parameters**	**CCC**	**95%CI**	**CCC**	**95%CI**	**CCC**	**95%CI**
AxialWSS	0,68	0,28- 0,88	0,93	0,82- 0,97	0,87	0,67- 0,96
MaxAxialWSS	0,10	0 - 0,19	0,06	-0,02- 0,13	0,94	0,82- 0,98
CircWSS	0,46	-0,07- 0,78	0,82	0,55- 0,94	0,86	0,63- 0,95
MaxCircumferentialWSS	0,20	-0,30- 0,61	0,21	-0,24- 0,59	0,88	0,69- 0,96
Flow	0,90	0,73- 0,97	0,98	0,96- 0,99	0,94	0,83- 0,98

MaximumFlow	0,90	0,71- 0,97	0,89	0,70- 0,96	0,98	0,96- 0,99
MaximumMeanVelocity	0,83	0,63- 0,92	0,77	0,47- 0,91	0,92	0,79- 0,97
MaxPressureGradient	0,89	0,70- 0,96	0, 78	0,53- 0,91	0,92	0,8- 0,97
MeanPressureGradient	0,89	0,70- 0,96	0,90	0,73- 0,96	0,97	0,91- 0,99
TotalVolume	0,90	0,73- 0,96	0,81	0,60- 0,92	0,93	0,83- 0,97

Siemens 3T	***operator 1 - operator 2***		***operator 1 - operator 3***		***operator 2 - operator 3***	
**4DFlow parameters**	**CCC**	**95%CI**	**CCC**	**95%CI**	**CCC**	**95%CI**
AxialWSS	0,73	0,37- 0,9	0,94	0,82- 0,98	0,70	-0,27- 0,47
MaxAxialWSS	0,90	0,78 - 0,96	0,11	-0,2- 0,4	0,11	-0,2- 0,4
CircWSS	0,61	0,15- 0,85	0,83	0,55- 0,94	0,67	0,25- 0,87
MaxCircumferentialWSS	0,17	-0,26- 0,54	0,002	-0,02- 0,02	0,004	-0,003- 0,01
Flow	0,96	0,88- 0,99	0,98	0,93- 0,99	0,96	0,89- 0,99
MaximumFlow	0,96	0,88- 0,99	0,99	0,95- 0,99	0,93	0,79- 0,98
MaximumMeanVelocity	0,83	0,63- 0,92	0,77	0,47- 0,91	0,92	0,79- 0,97
MaxPressureGradient	0,89	0,70- 0,96	0, 78	0,53- 0,91	0,92	0,8- 0,97
MeanPressureGradient	0,89	0,70- 0,96	0,90	0,73- 0,96	0,97	0,91- 0,99
TotalVolume	0,90	0,73- 0,96	0,81	0,60- 0,92	0,93	0,83- 0,97

1.1 Supplementary Figures

Supplementary Figures S1: Correlation plots. For each operator and for each device, Spearman's correlation between 4D-Flow parameters is reported. The circle size is scaled by the correlation coefficient value. Blue and red colours designate positive and negative correlations, respectively. (*$P < 0.05$; **$P < 0.01$; ***$P < 0.001$).

Supplementary Figures S2: Bland – Altman plot of measures for each pair of raters. The dashed line represents the mean difference along with the 95% CI. The 95% limits of agreement are also presented on the plot.

Supplementary Figure S3: Inter device agreement of MaxMeanVel and TV.

For each parameter (MaxMeanVel and TV), boxplots of Kruskal-Wallis test show the comparison of measurements from 3 devices (Philips 1.5 T, Philips 3 T and Siemens 3 T) using data by operator 1, operator 2 and operator 3.

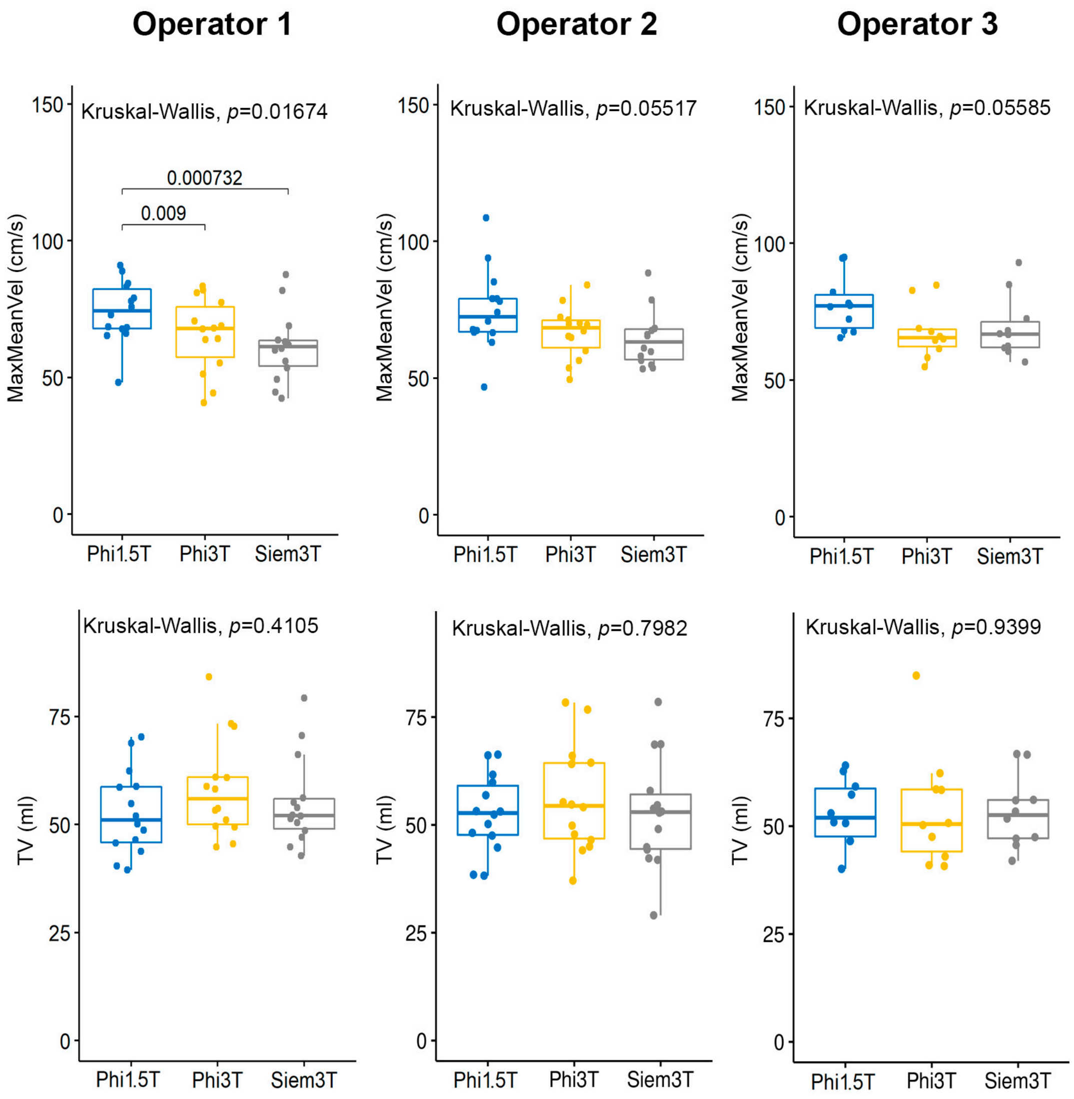
Operator 1
Operator 2
Operator 3
Kruskal-Wallis, p=0.01674
0.000732
0.009
Kruskal-Wallis, p=0.05517
Kruskal-Wallis, p=0.05585
MaxMeanVel (cm/s)
150
100
50
0
Phi1.5T
Phi3T
Siem3T
Kruskal-Wallis, p=0.4105
Kruskal-Wallis, p=0.7982
Kruskal-Wallis, p=0.9399
TV (ml)
75
50
25
0

Supplementary Figure S4: Inter device agreement of PV, MaxFlow, MaxPressGrad and MeanPressGrad.

For each parameter (PV, MaxFlow, MaxPressGrad, MeanPressGrad), boxplots of Kruskal-Wallis test show the comparison of measurements from 3 devices (Philips 1.5 T, Philips 3 T and Siemens 3 T) using data by operator 1, operator 2 and operator 3.

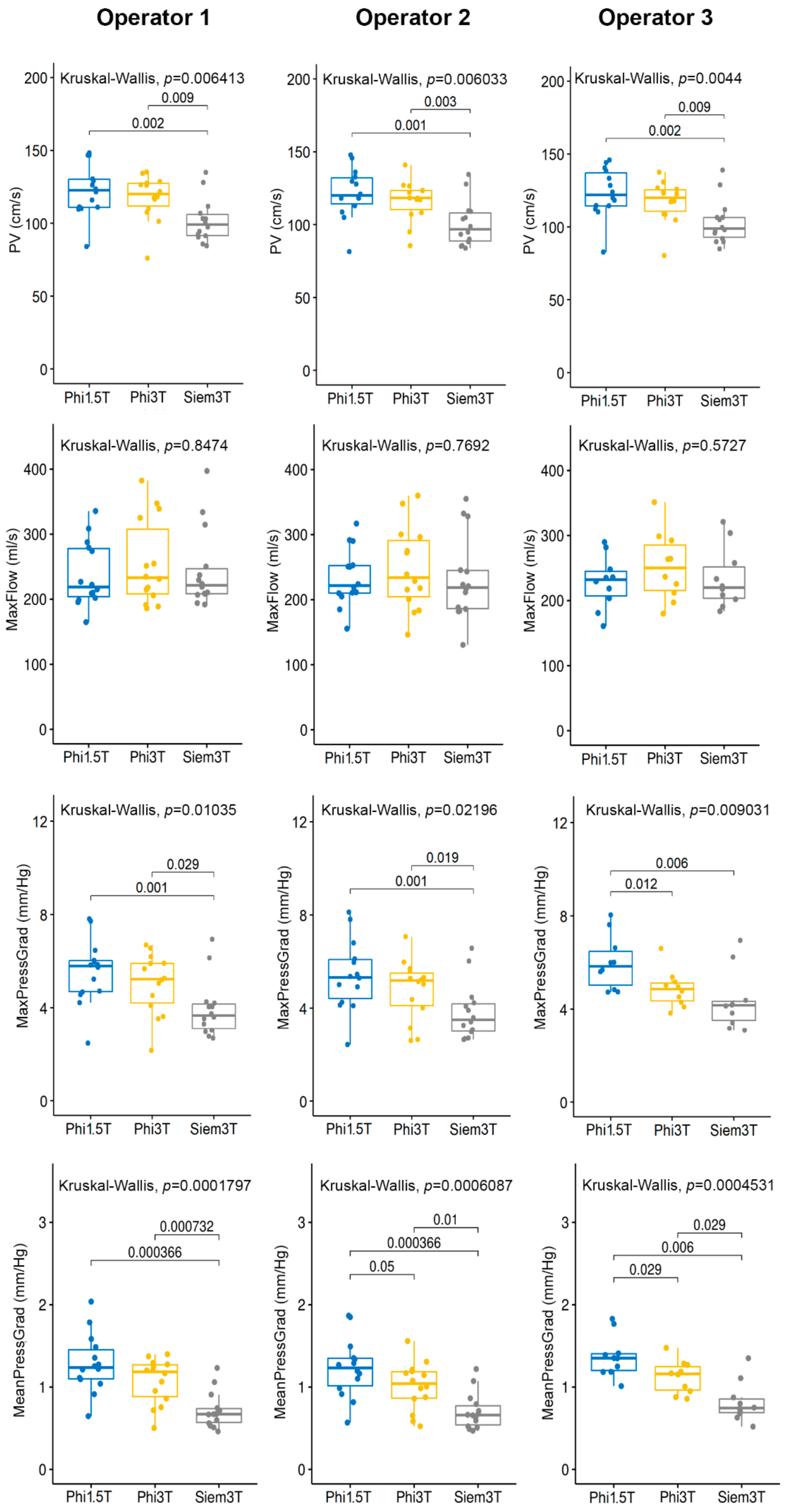

Operator 1
Operator 2
Operator 3
Kruskal-Wallis, p=0.006413
0.009
0.002
Kruskal-Wallis, p=0.006033
0.003
0.001
Kruskal-Wallis, p=0.0044
0.009
0.002
PV (cm/s)
Kruskal-Wallis, p=0.8474
Kruskal-Wallis, p=0.7692
Kruskal-Wallis, p=0.5727
MaxFlow (ml/s)
Kruskal-Wallis, p=0.01035
0.029
0.001
Kruskal-Wallis, p=0.02196
0.019
0.001
Kruskal-Wallis, p=0.009031
0.006
0.012
MaxPressGrad (mm/Hg)
Kruskal-Wallis, p=0.0001797
0.000732
0.000366
Kruskal-Wallis, p=0.0006087
0.01
0.000366
0.05
Kruskal-Wallis, p=0.0004531
0.029
0.006
0.029
MeanPressGrad (mm/Hg)
Phi1.5T
Phi3T
Siem3T
