# Supplementary material for: 4D-Flow Cardiovascular Magnetic Resonance Sequence for Aortic Assessment: Multi-Vendor and Multi-Magnetic Field Reproducibility in Healthy Volunteers
Source: J Clin Med. 2023 Apr 19;12(8):2960. doi: 10.3390/jcm12082960 (PMC10141060; doi:10.3390/jcm12082960)

AxialWSS

Phi1.5T

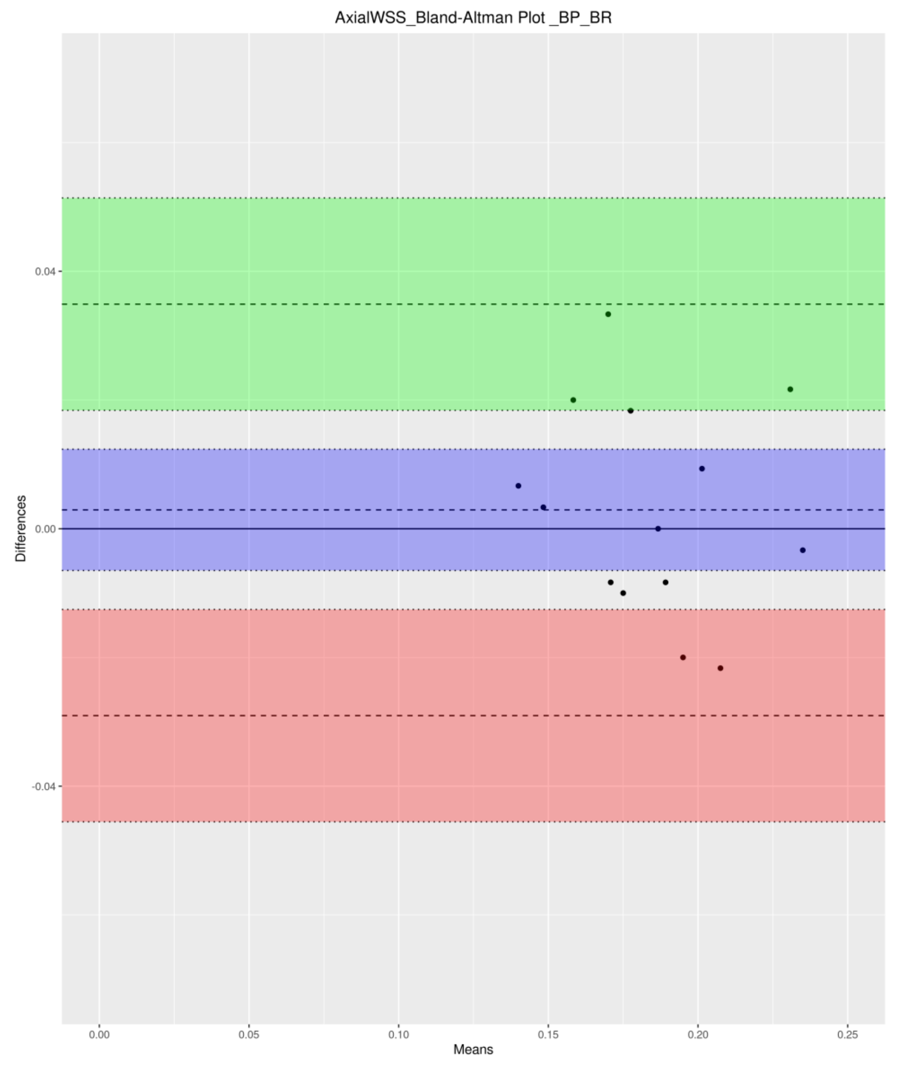

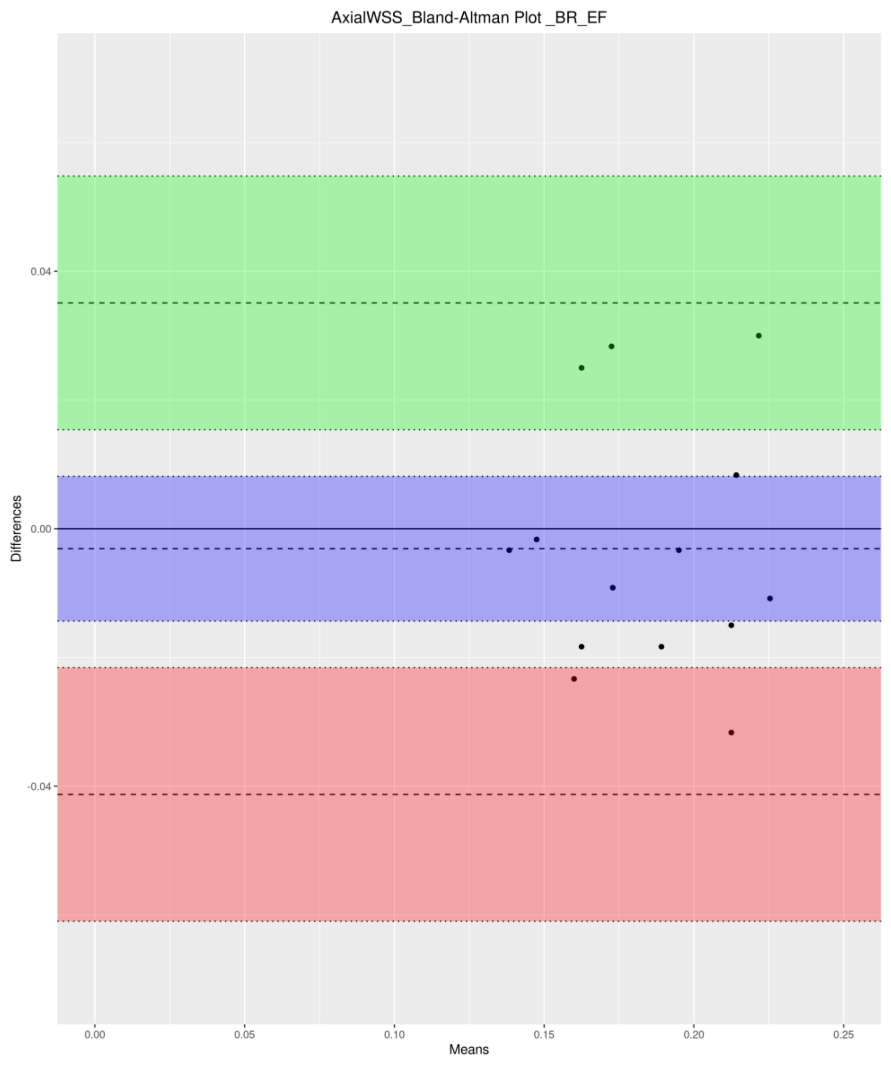

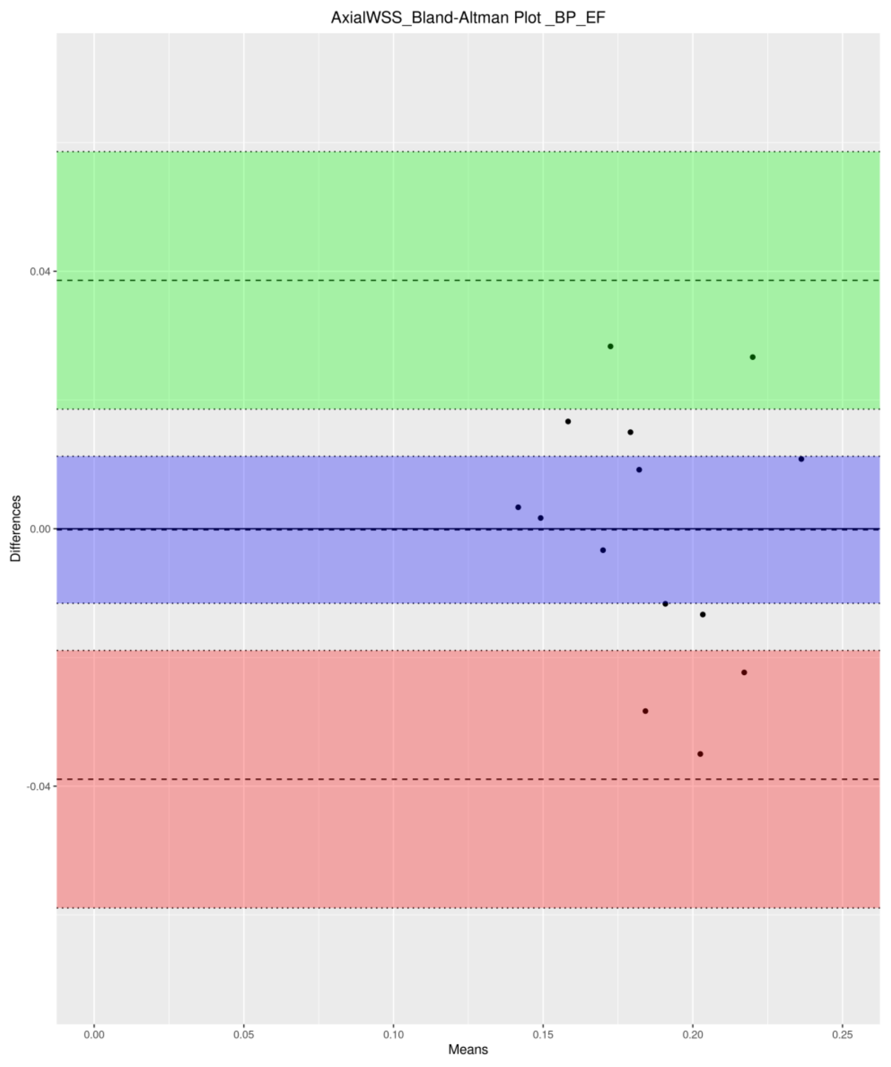

Phi3T

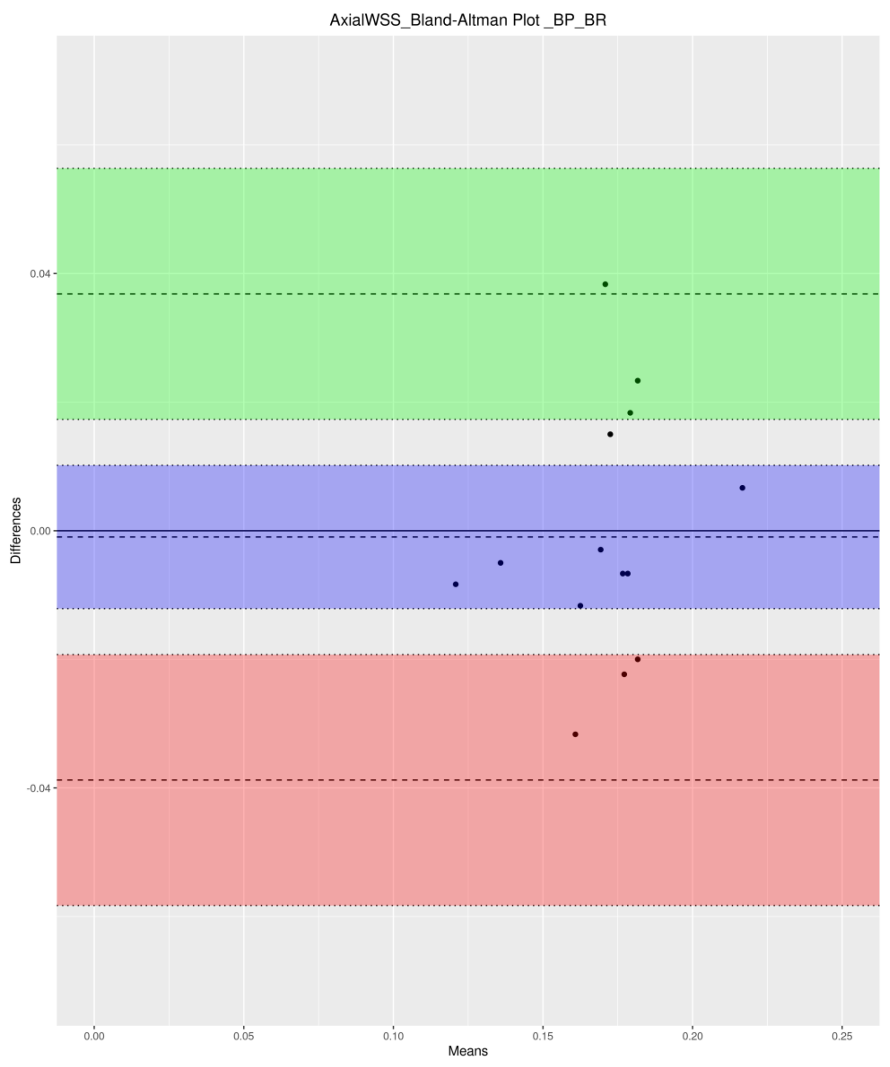

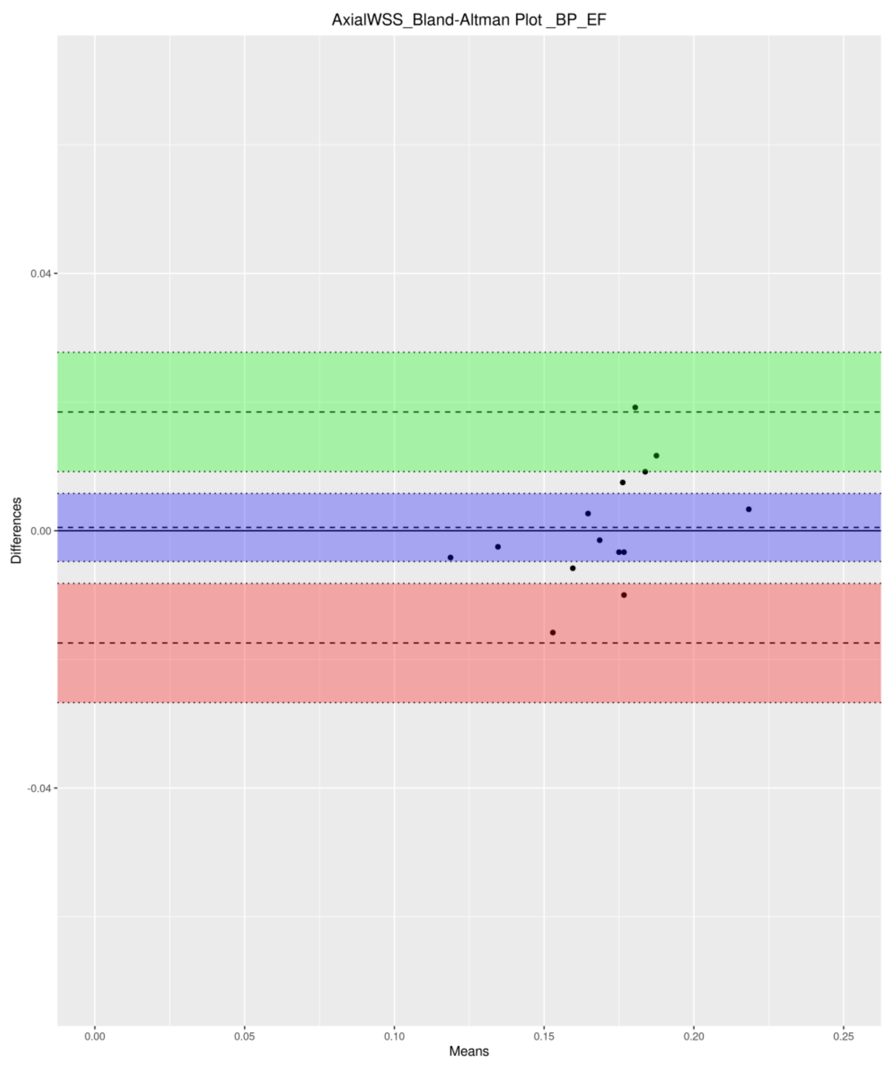

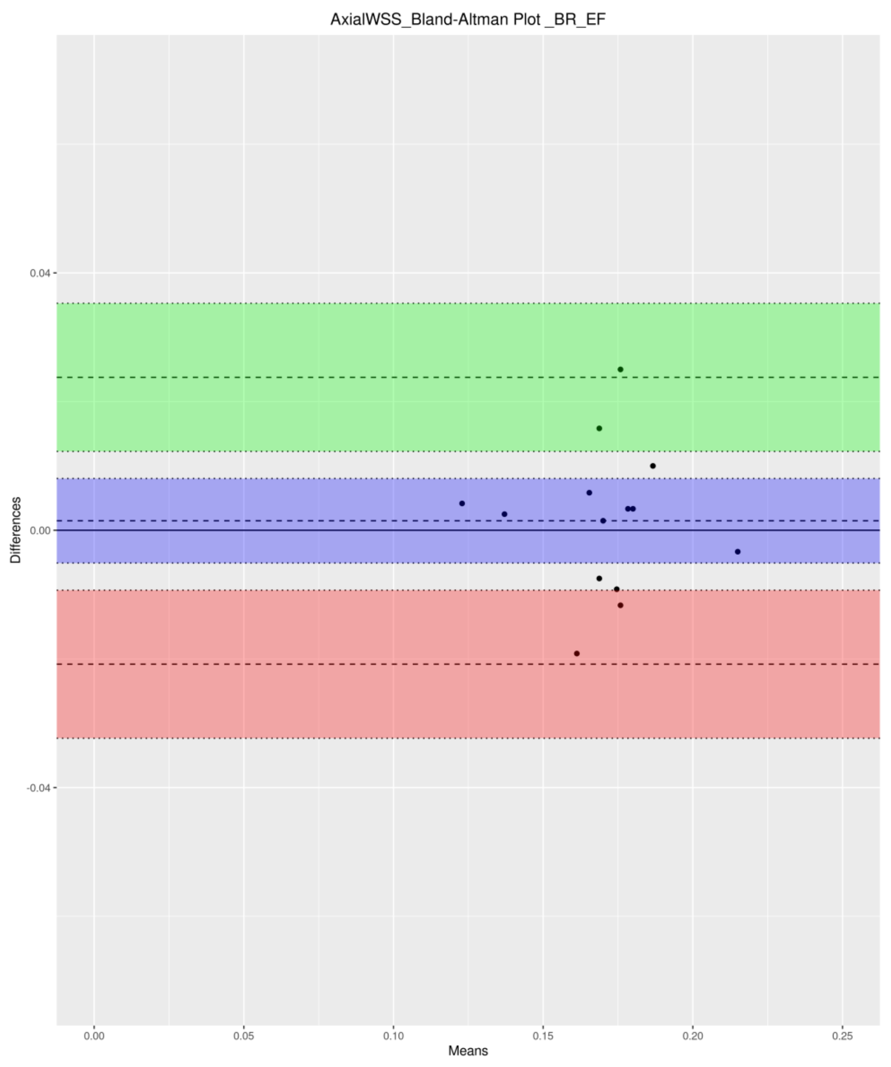

Siem3T

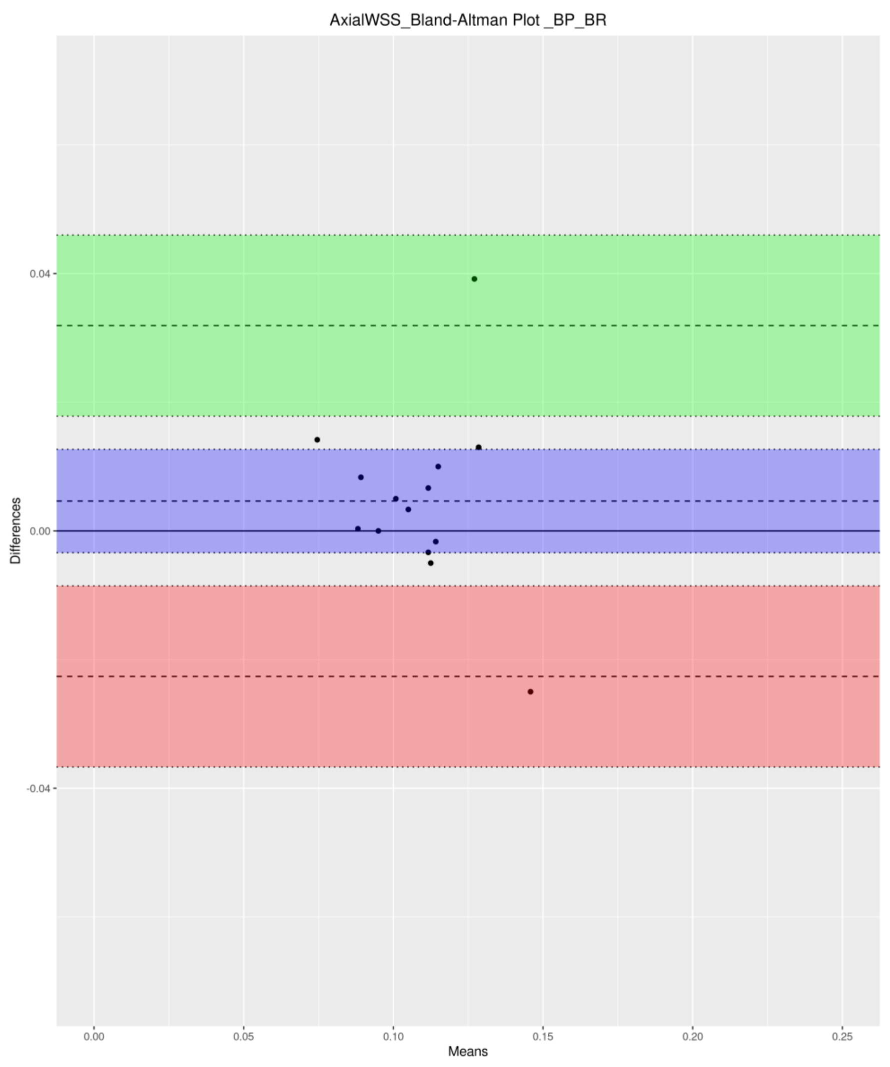

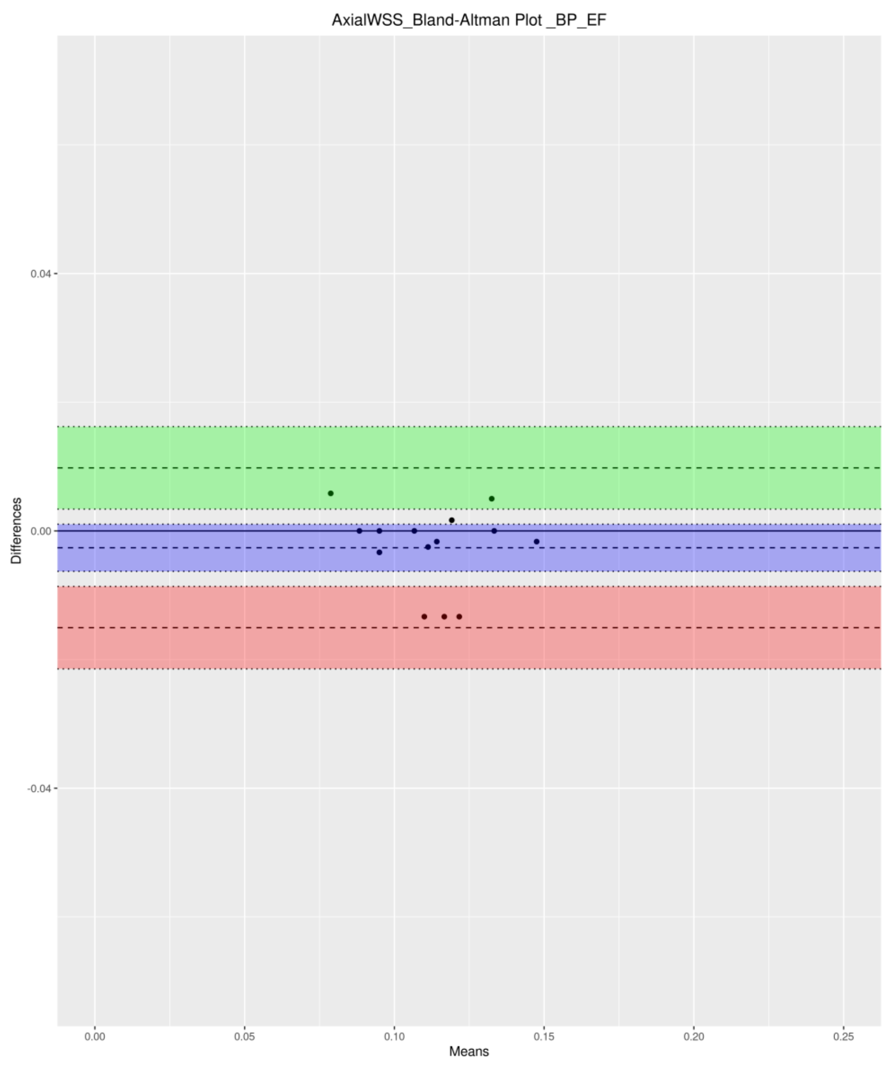

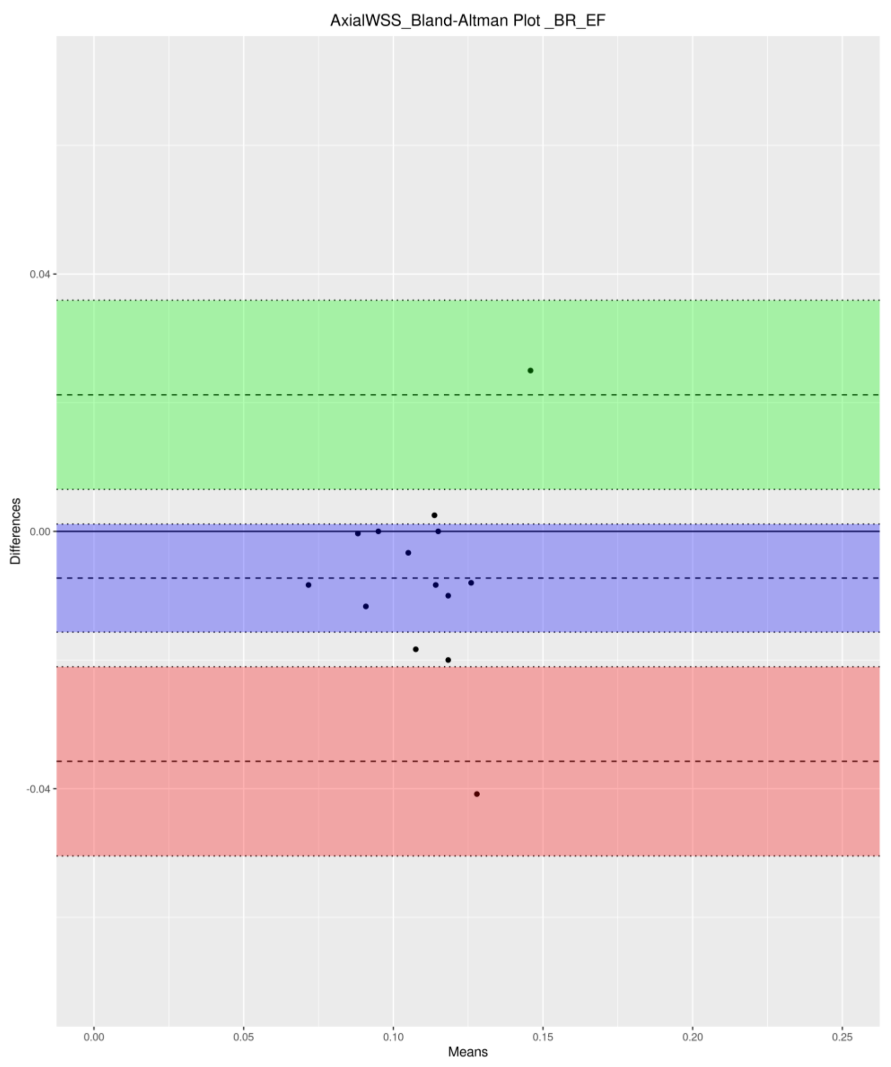

CircWSS

Phi1.5T

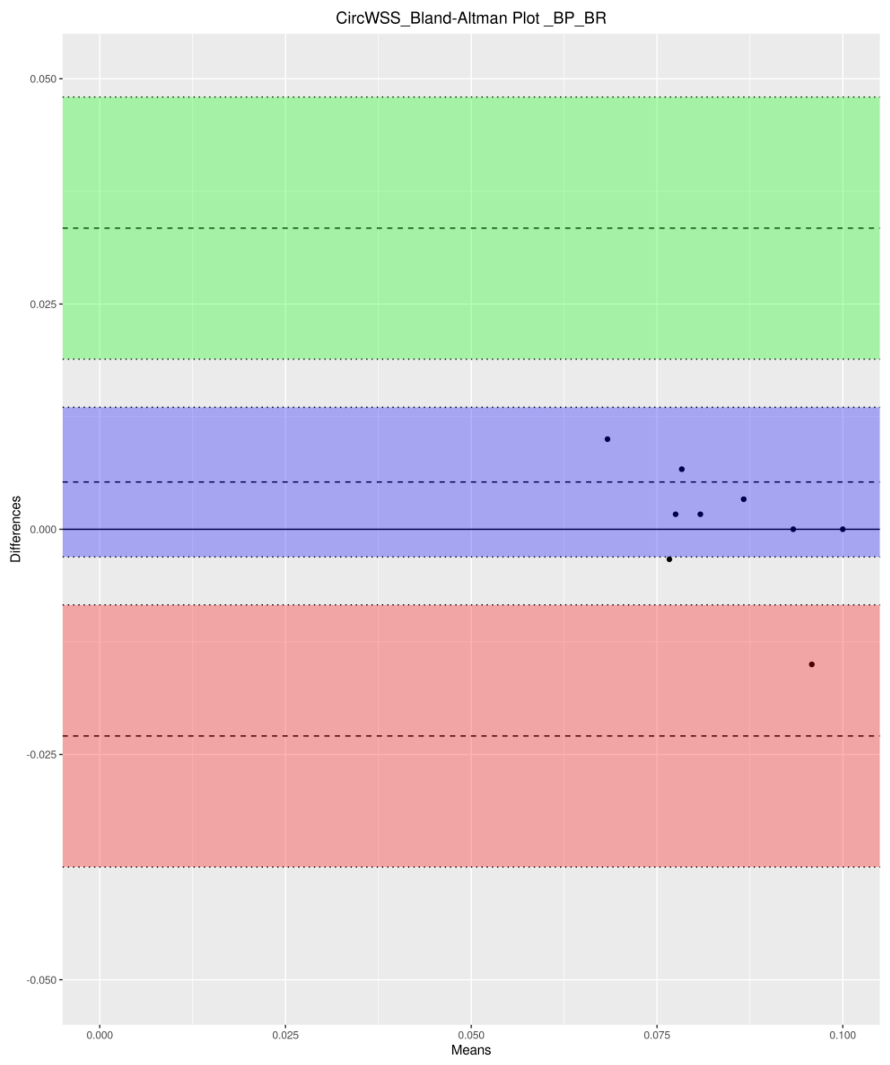

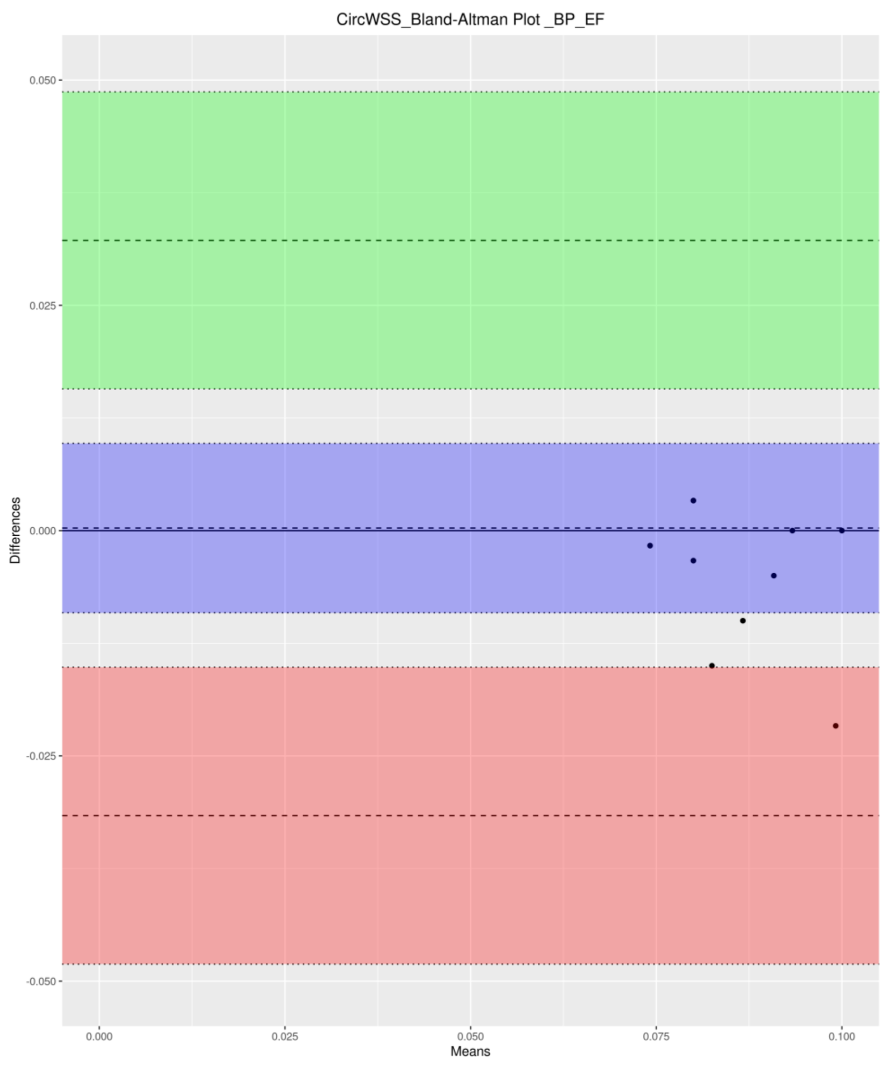

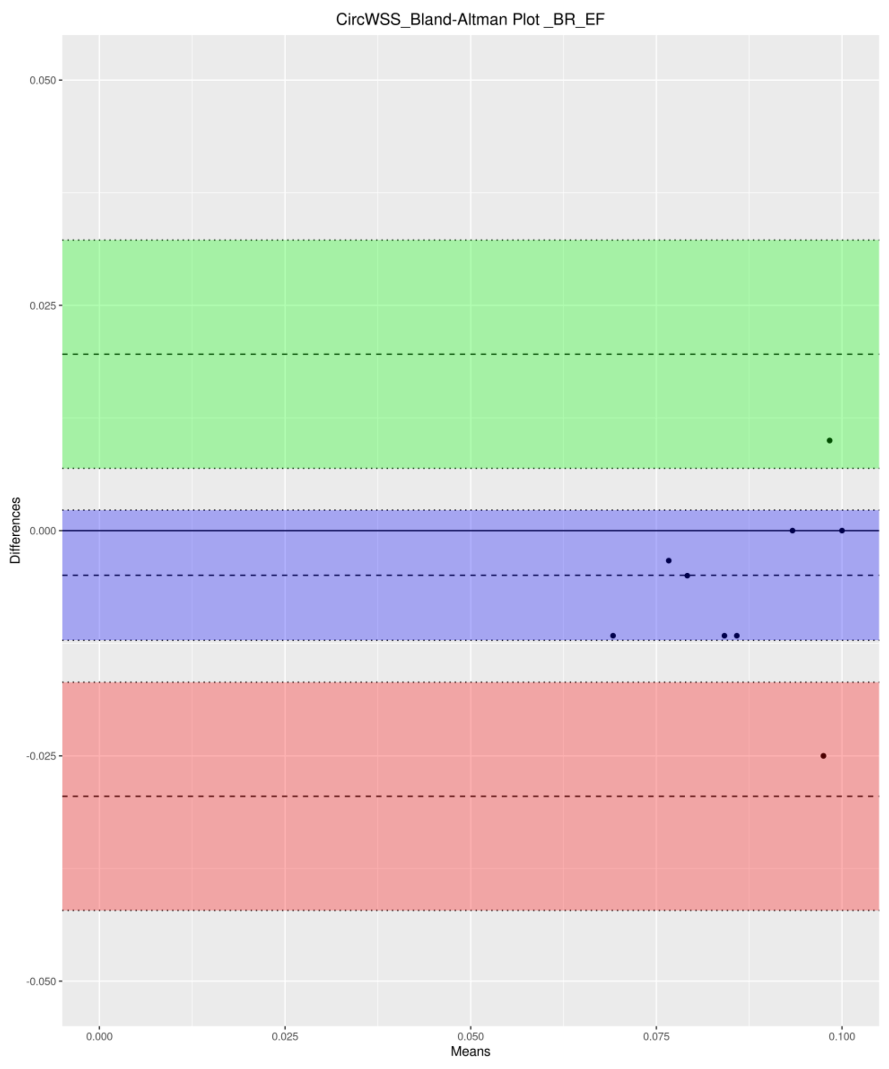

Phi3T

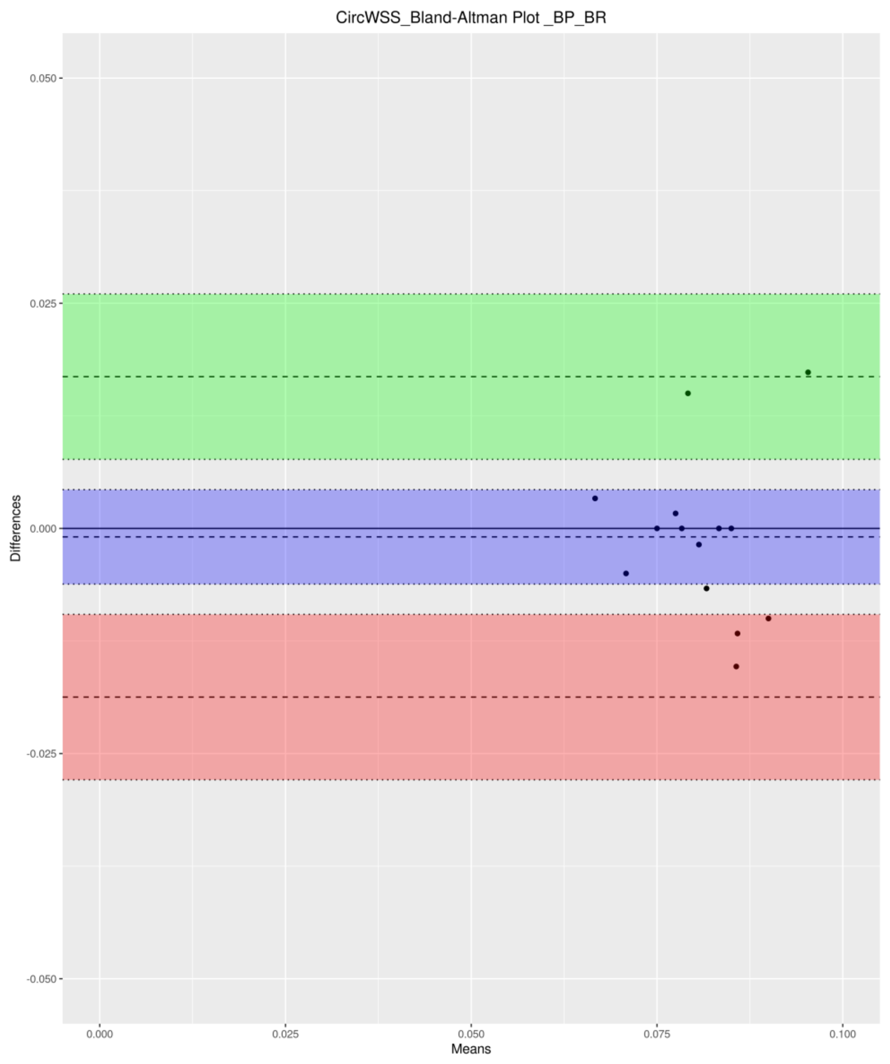

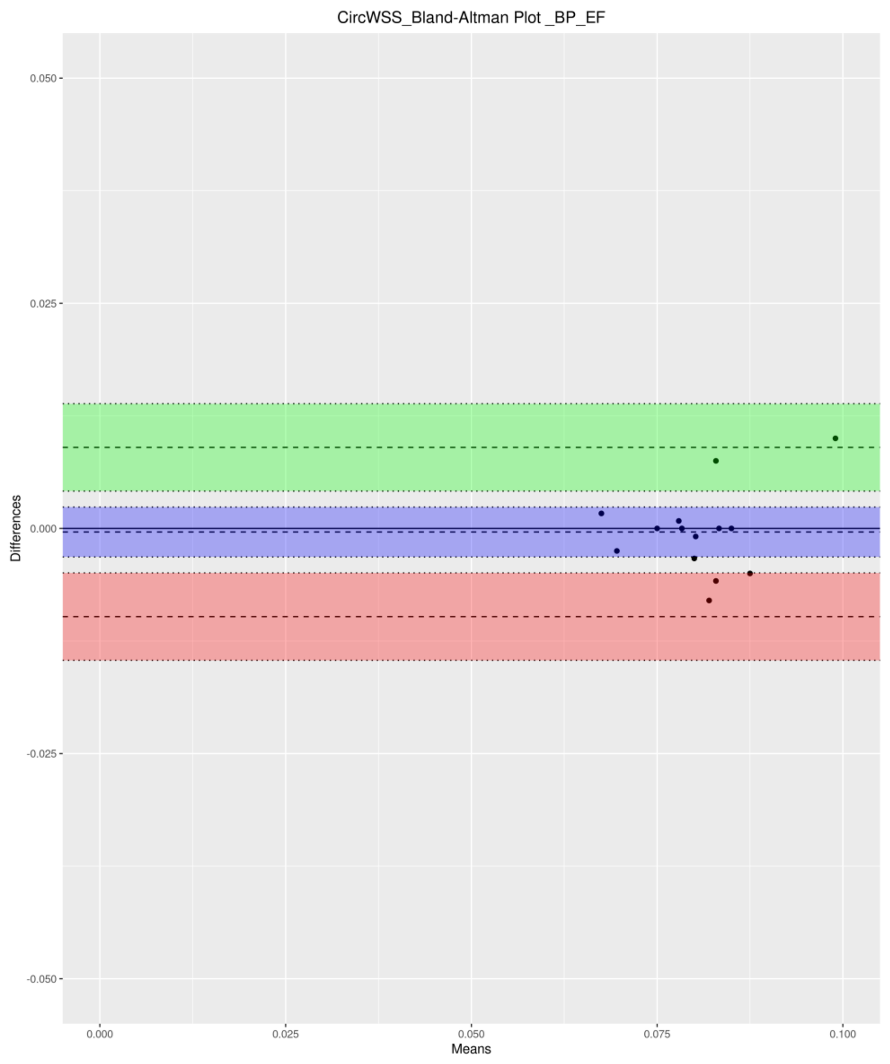

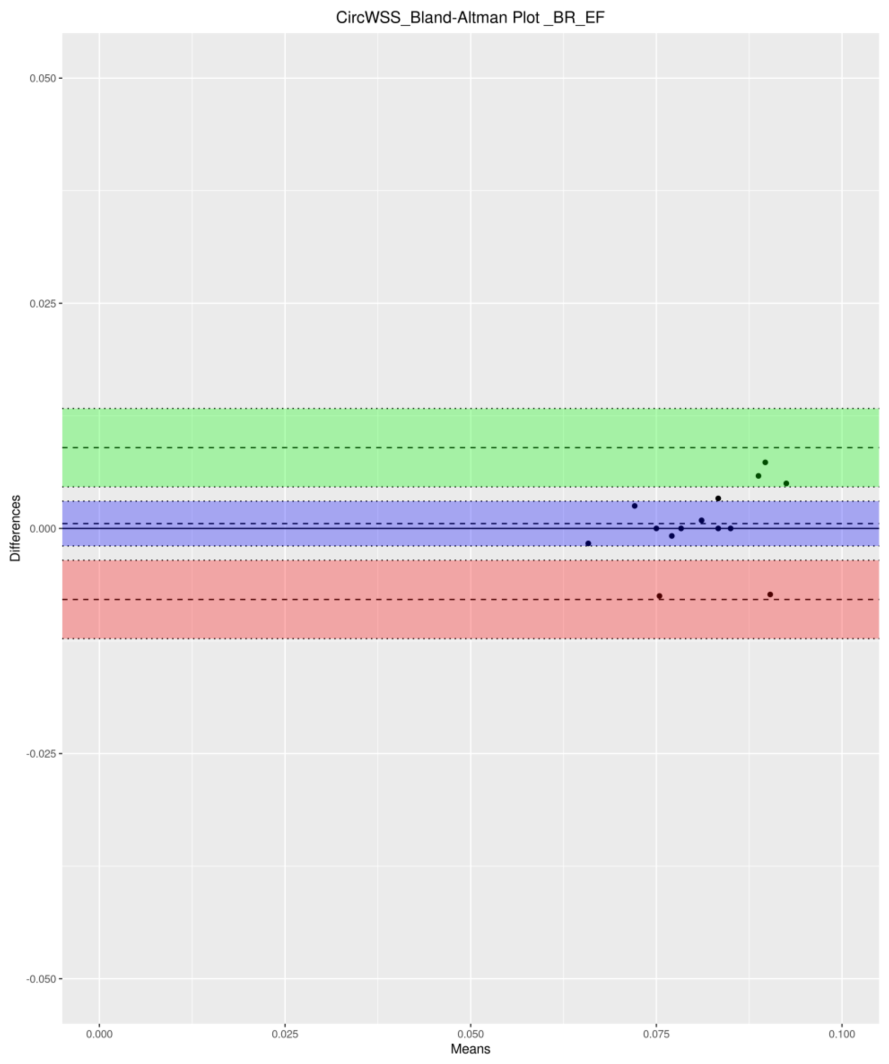

Siem3T

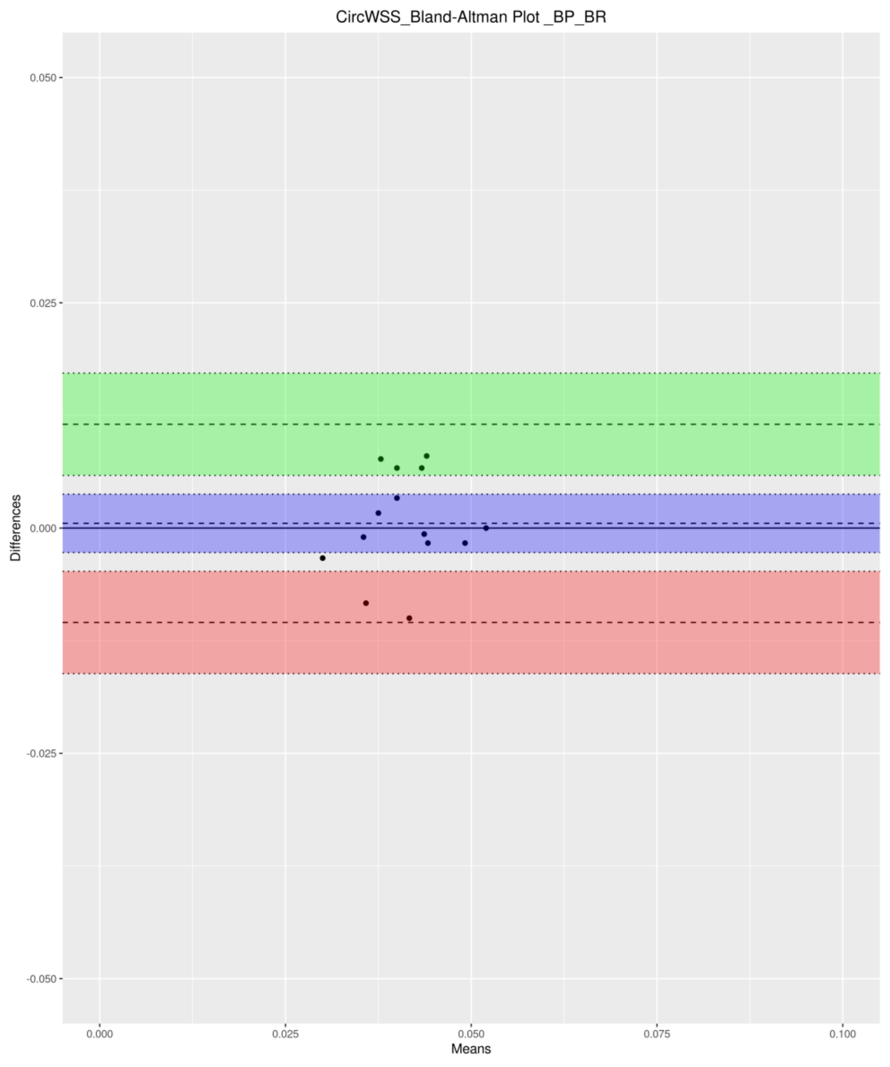

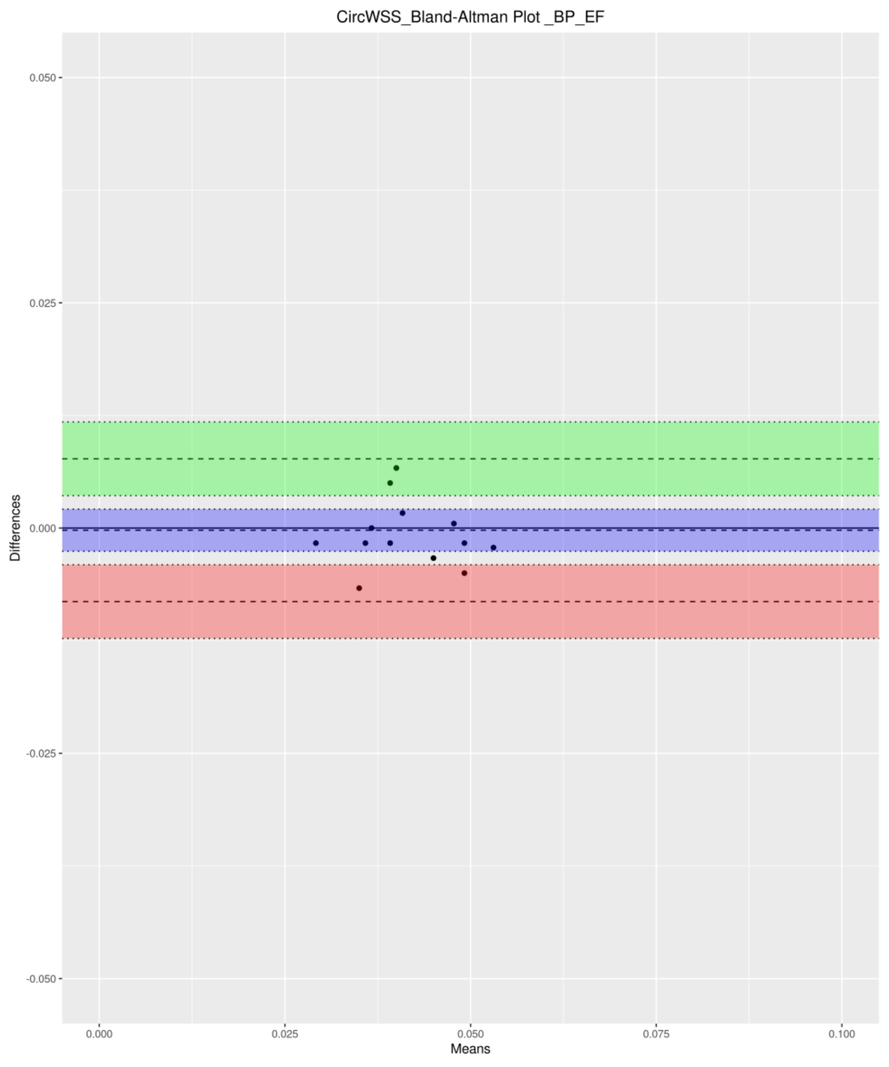

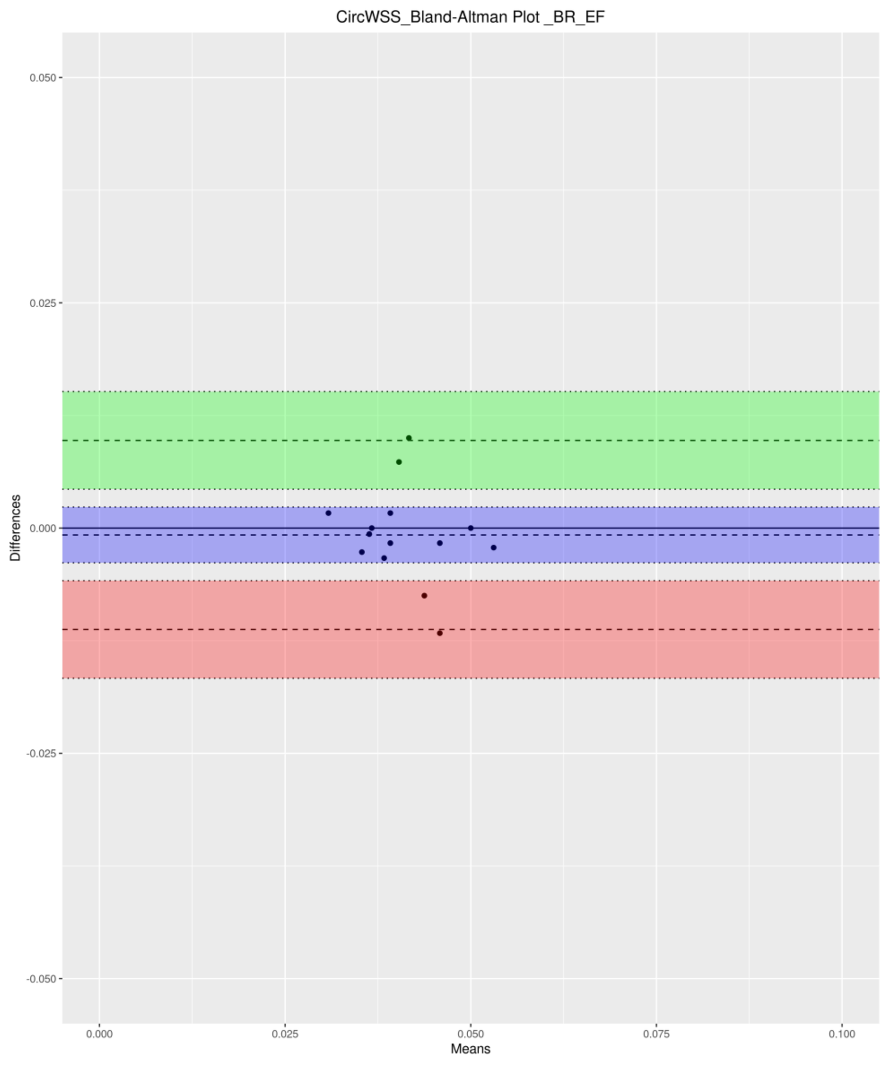

Flow

Phi1.5T

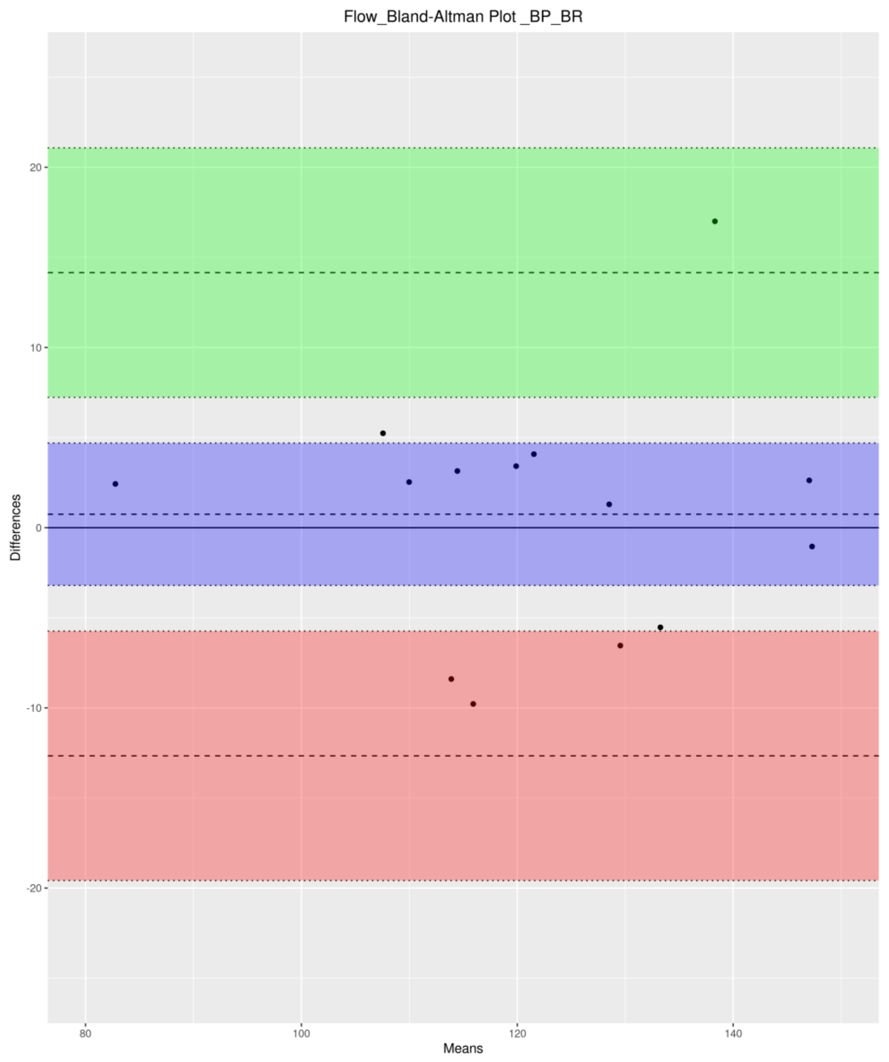

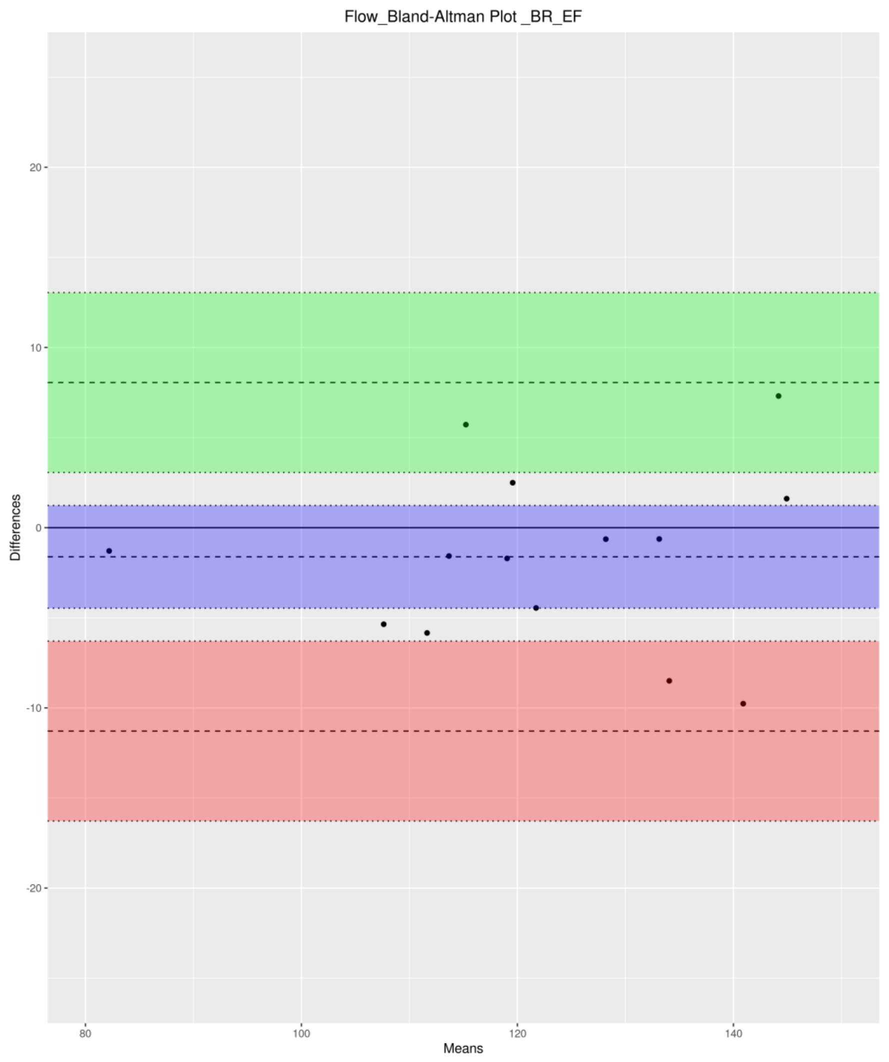

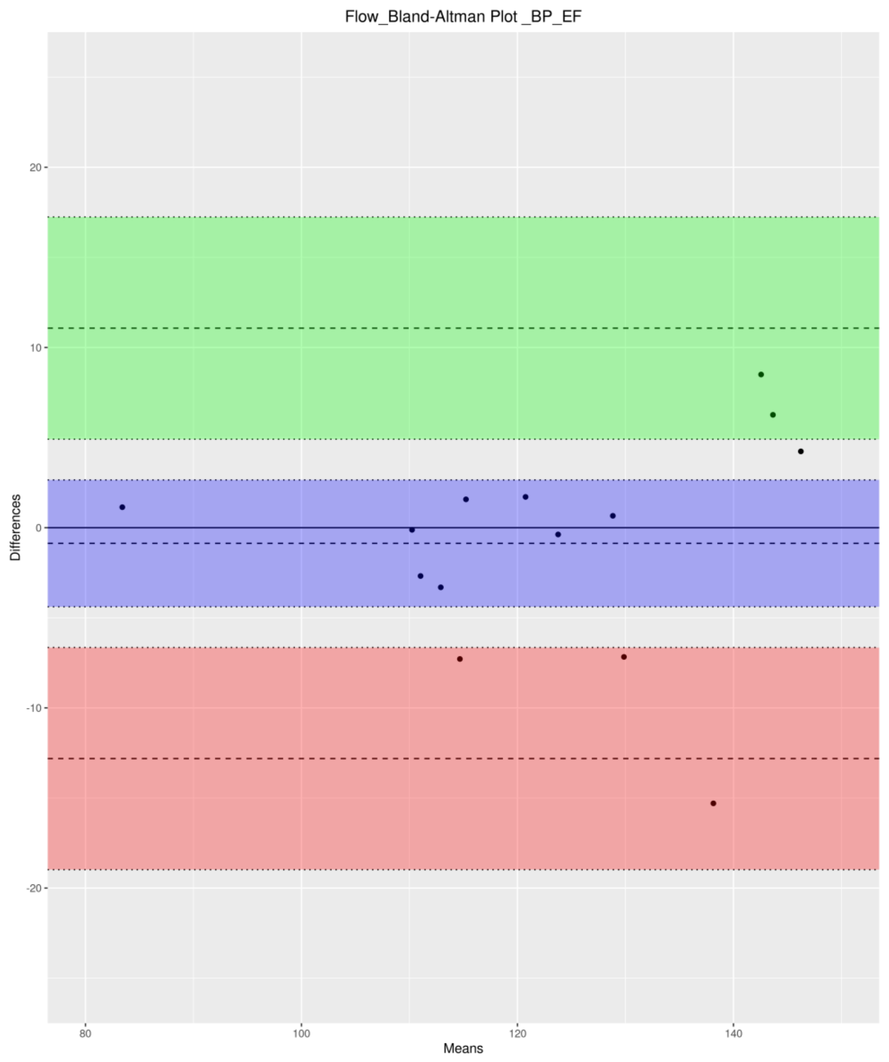

Phi3T

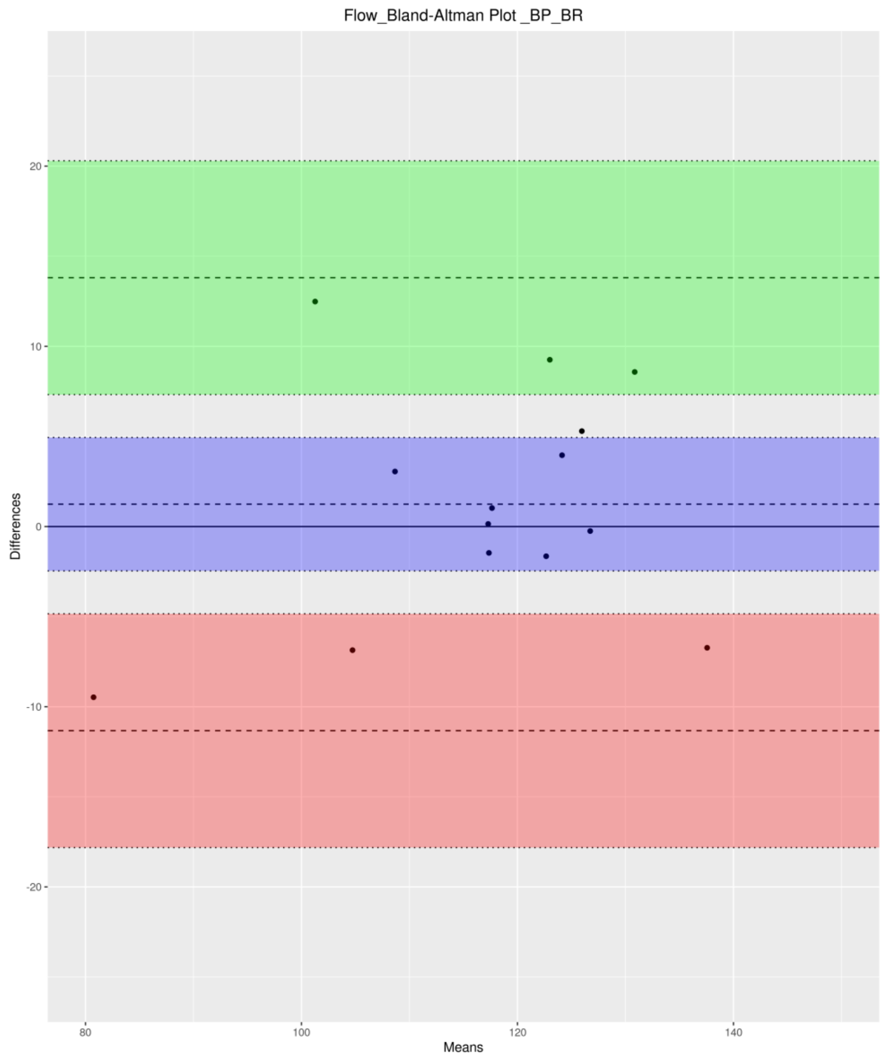

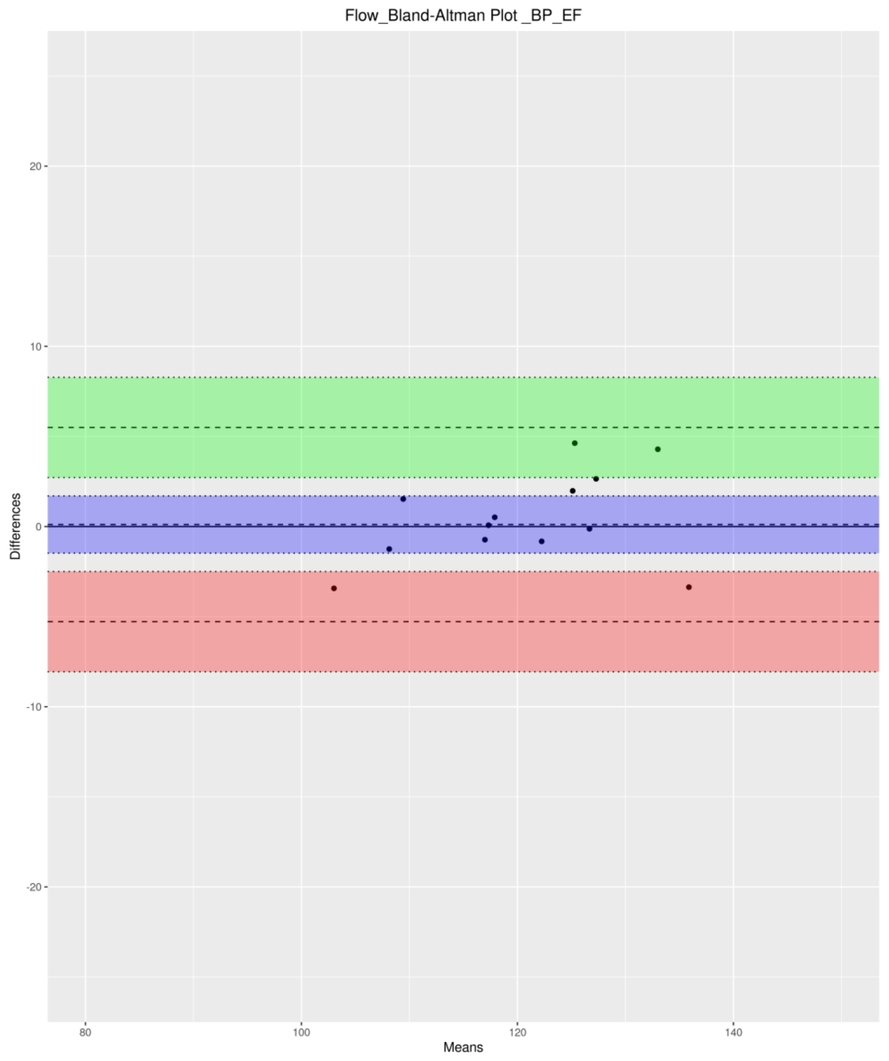

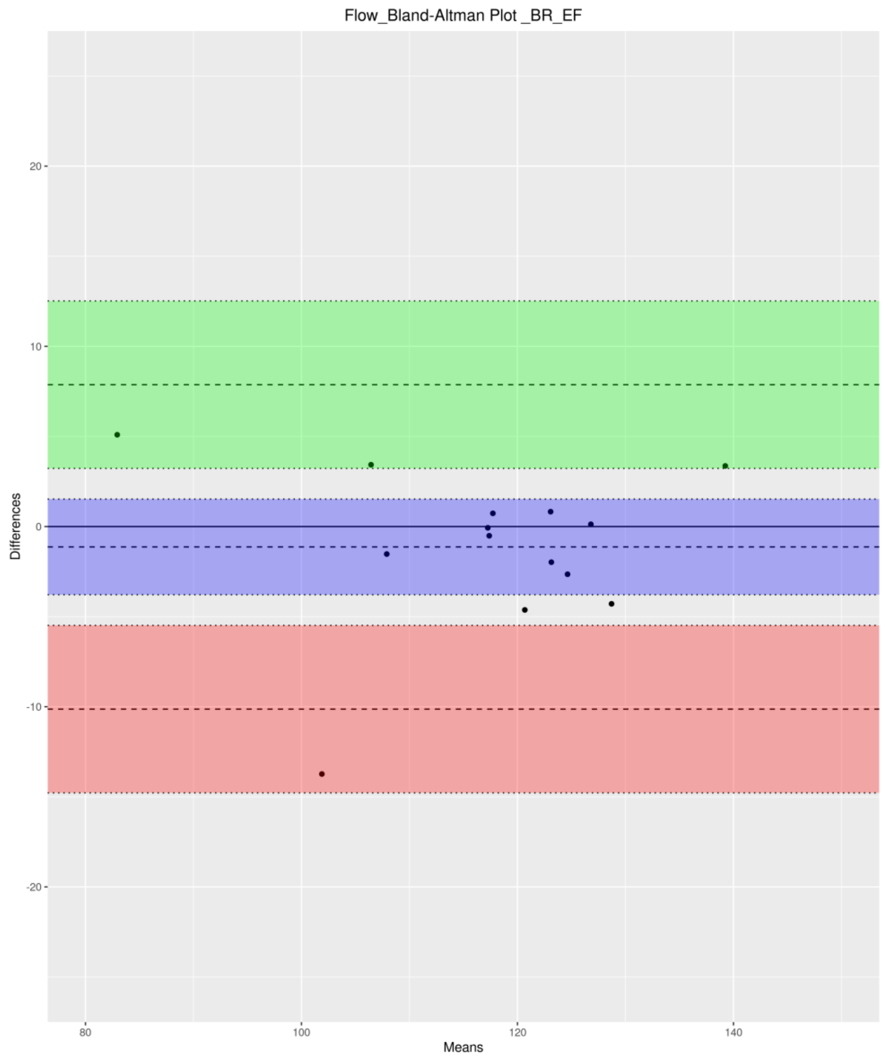

Siem3T

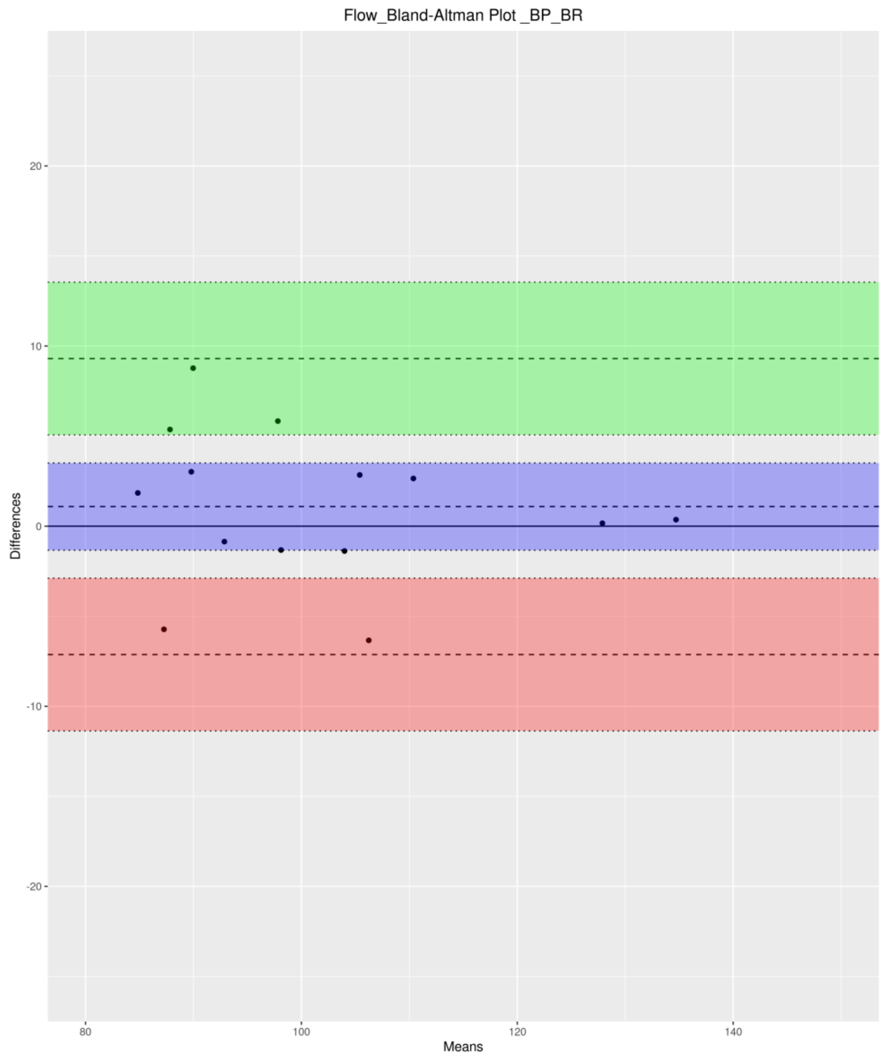

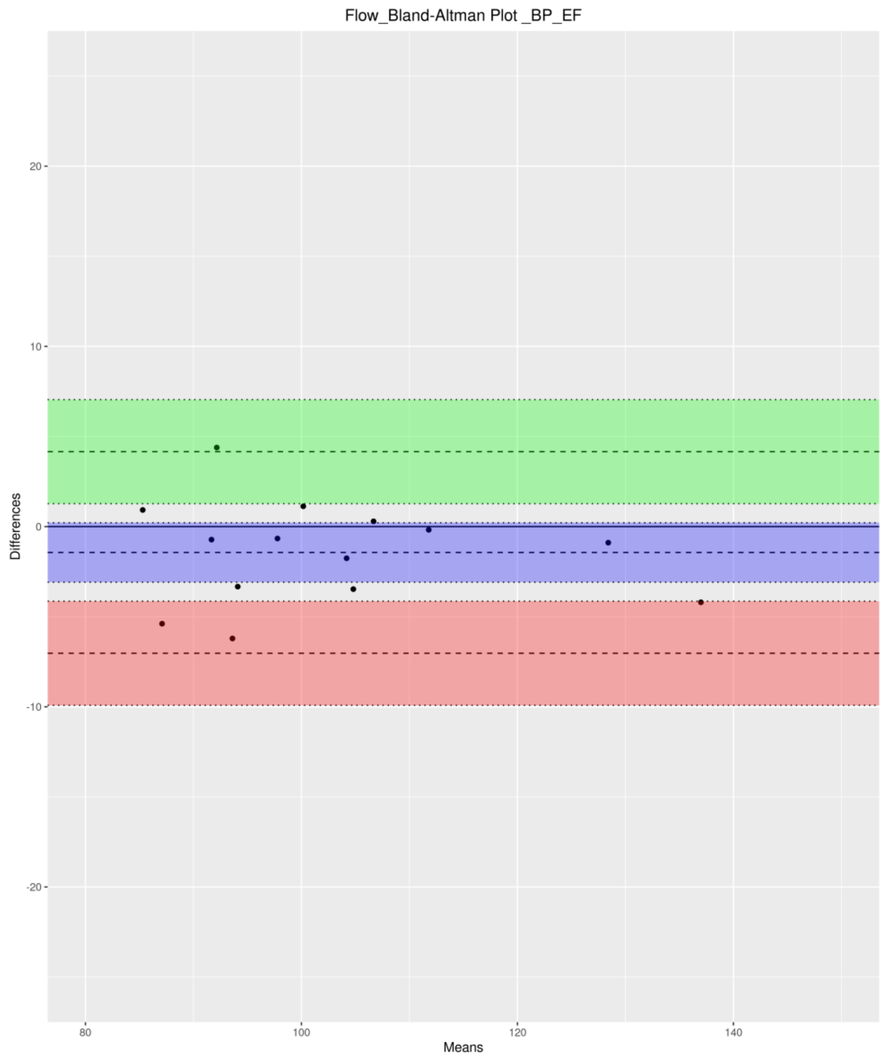

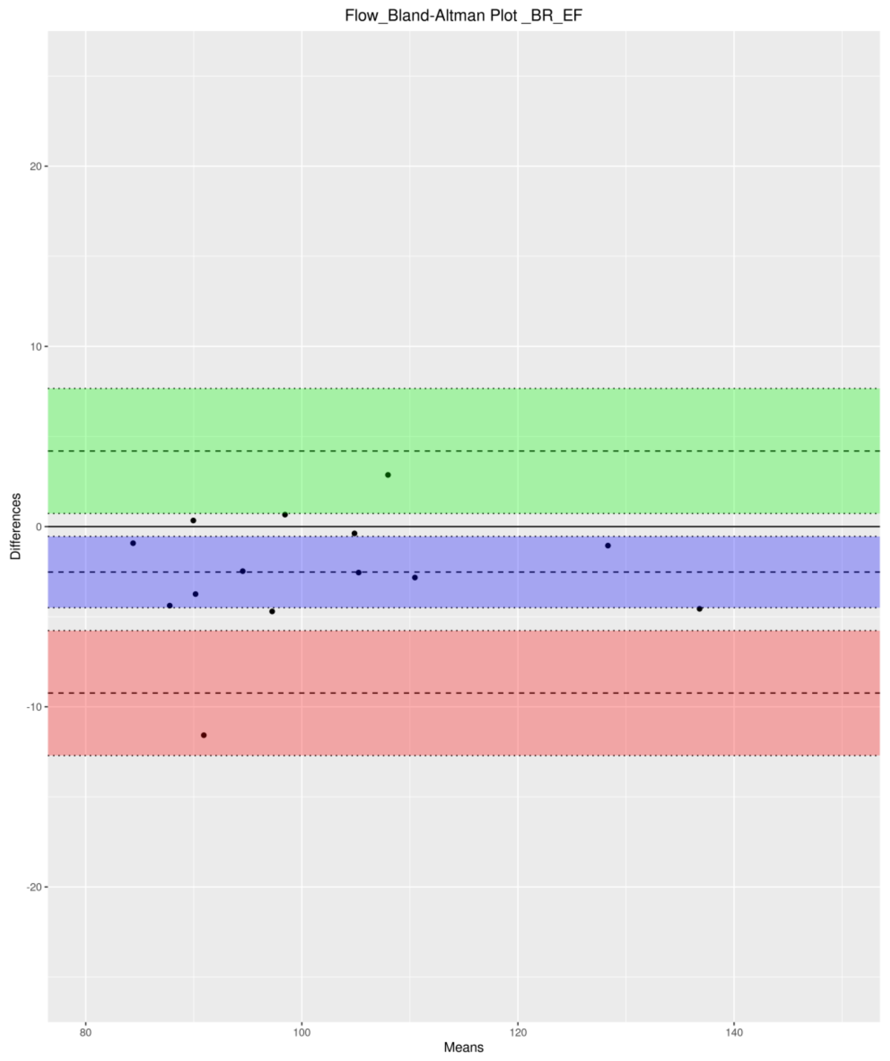

MaxAxialWallShearStressAllPhases

Phi1.5T

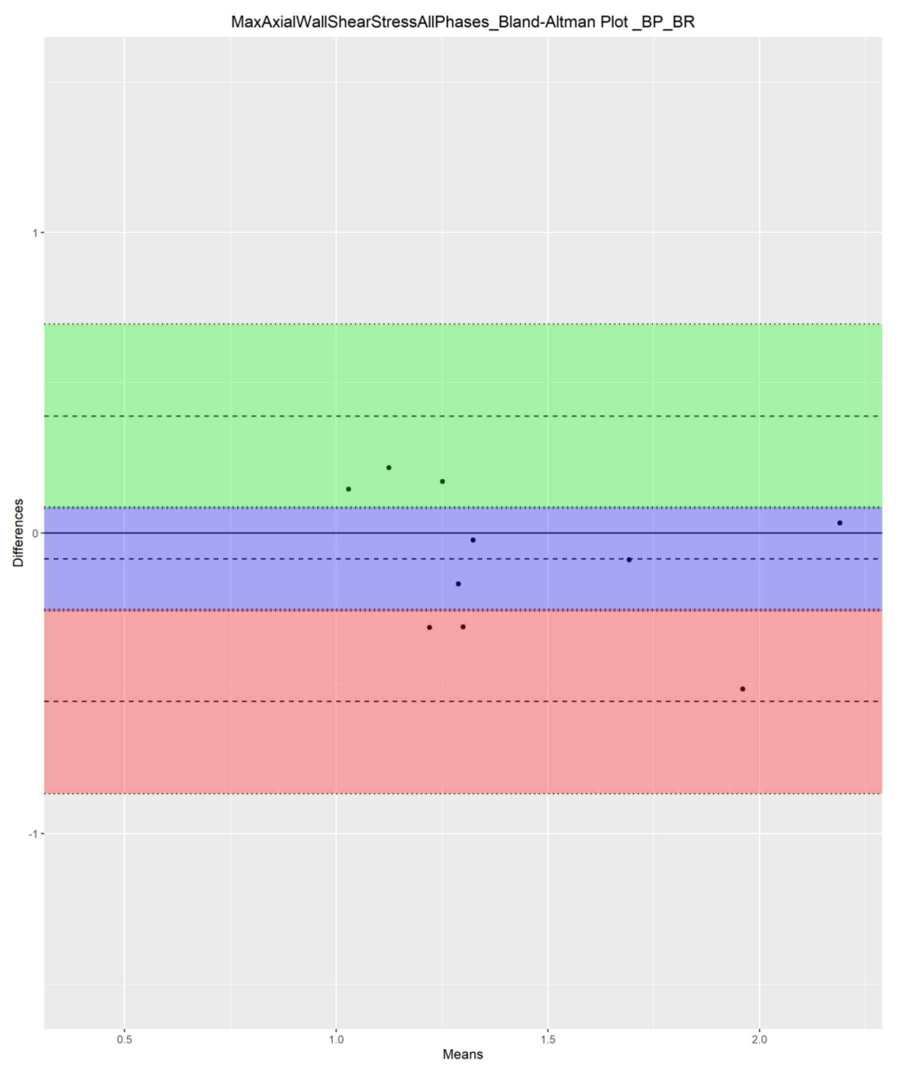

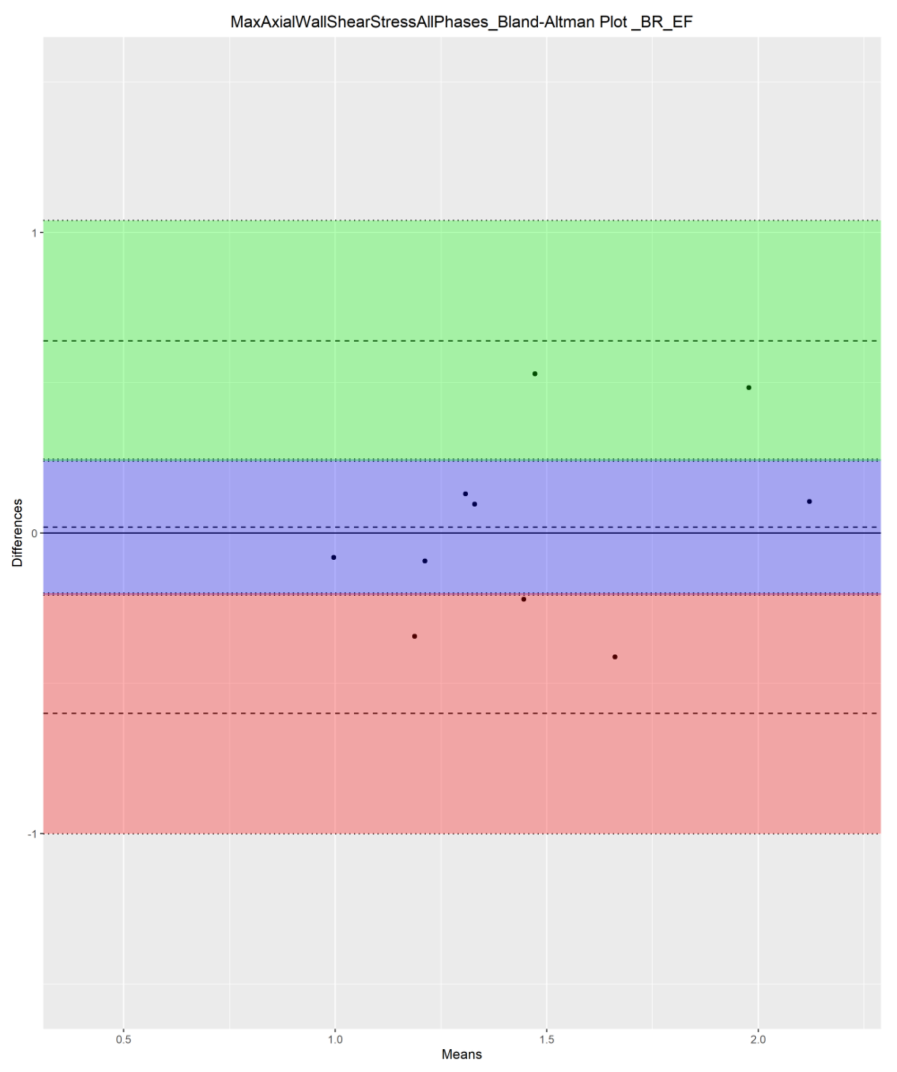

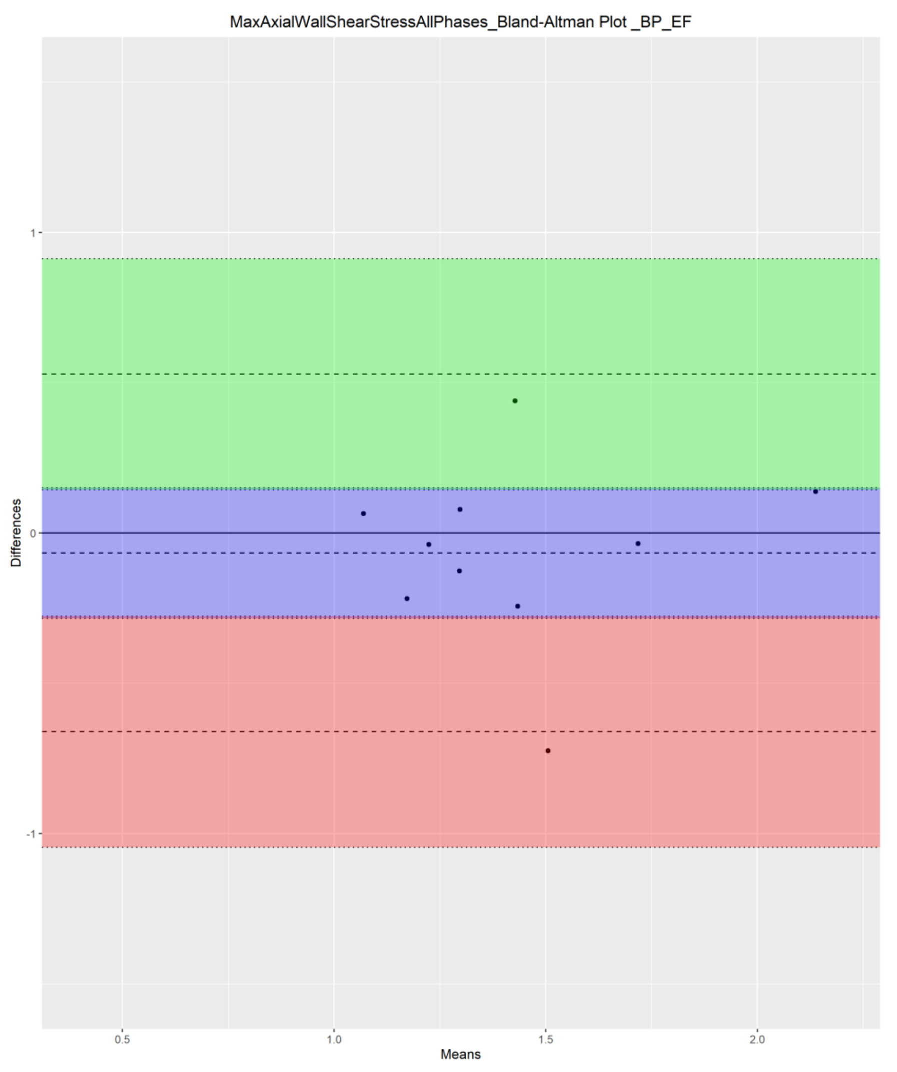

Phi3T

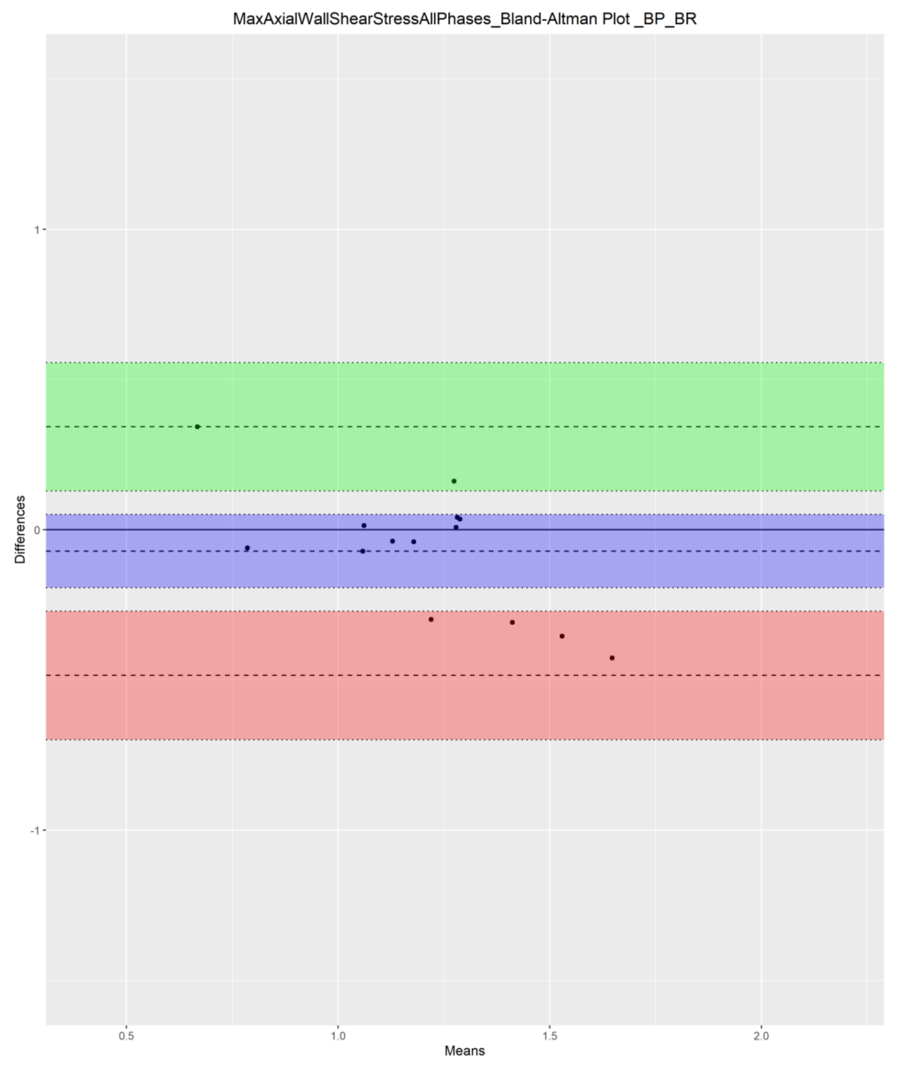

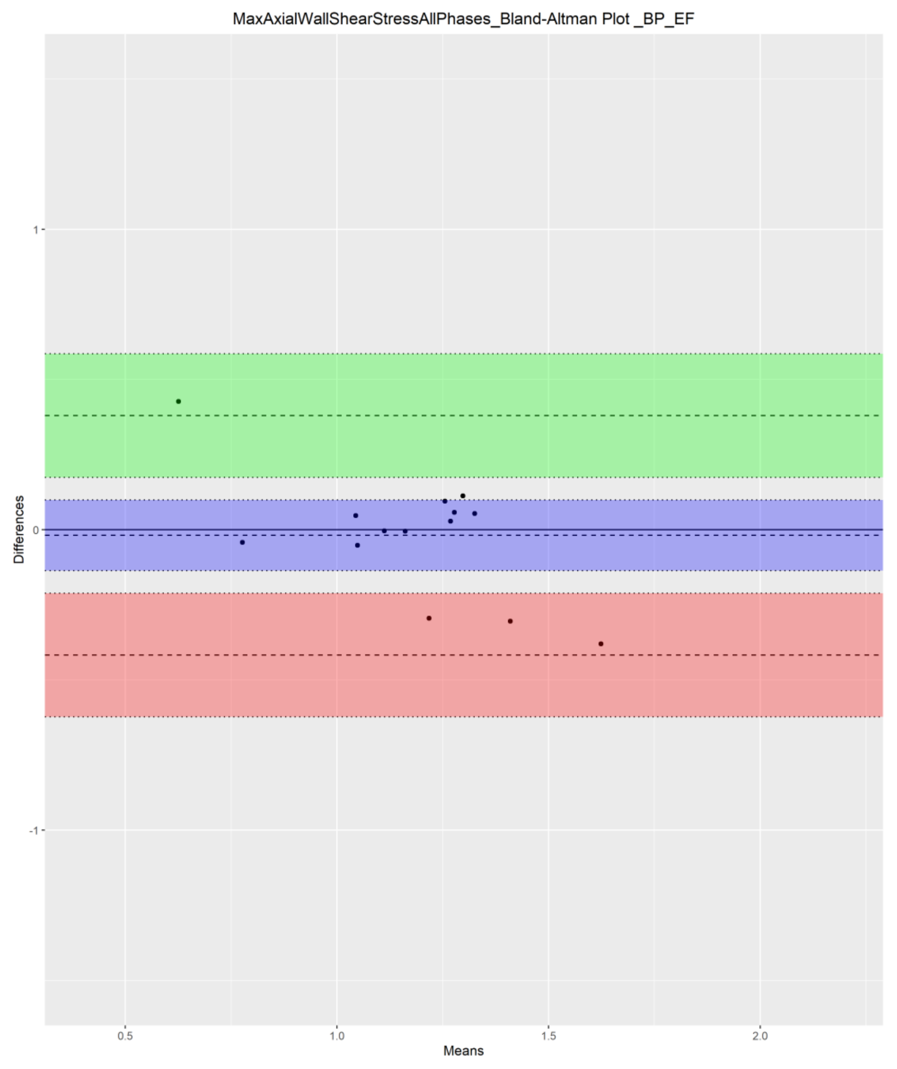

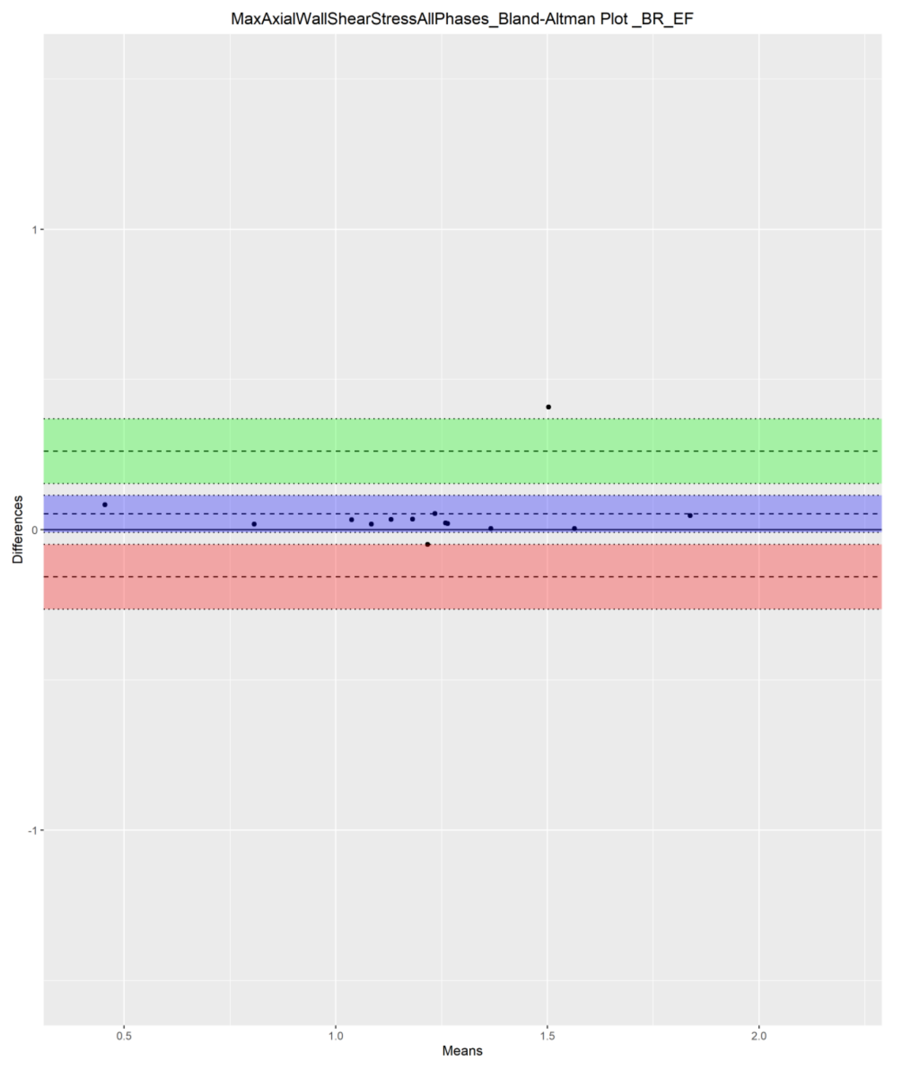

Siem3T

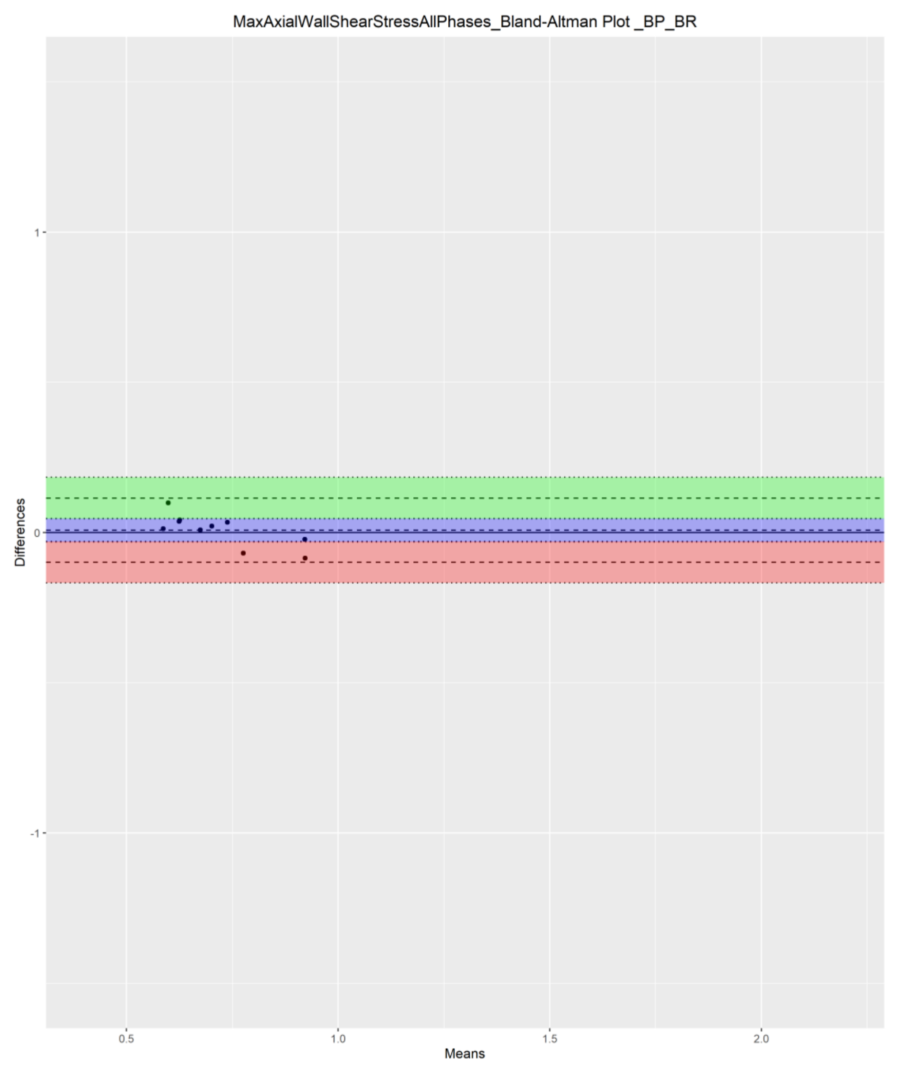

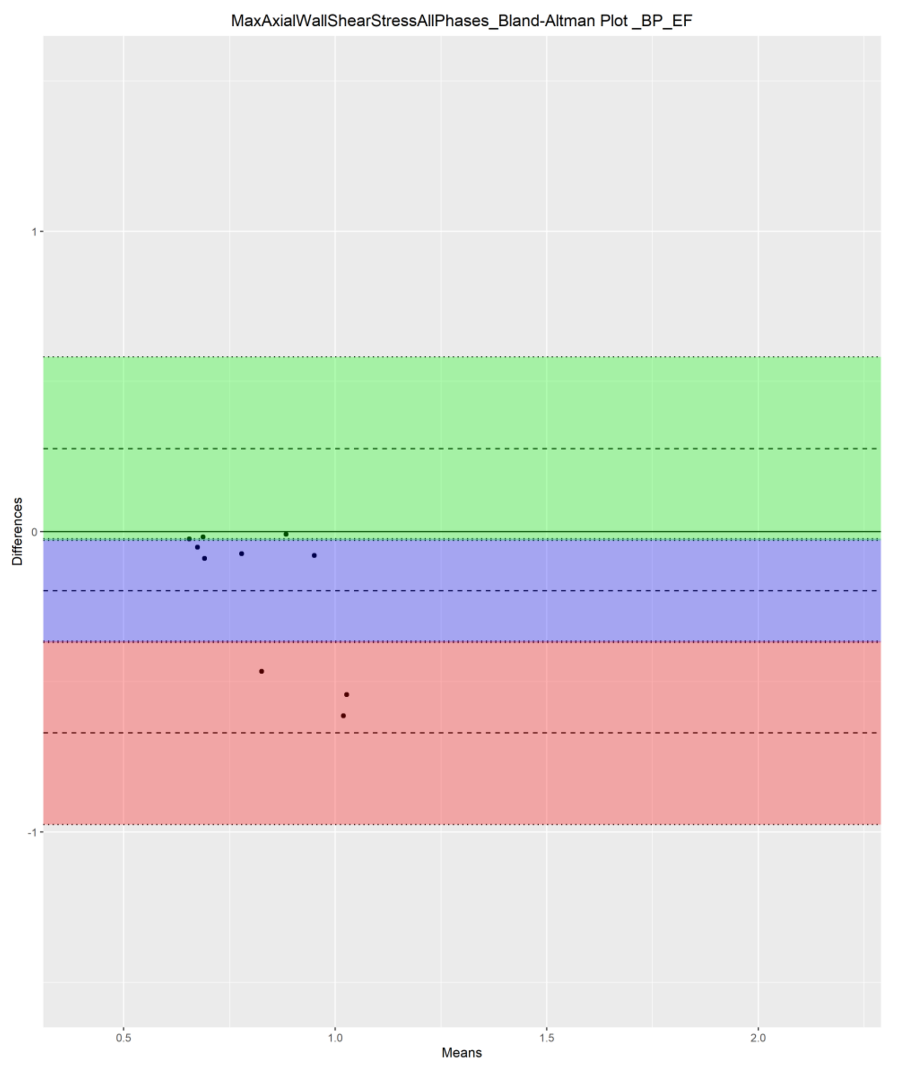

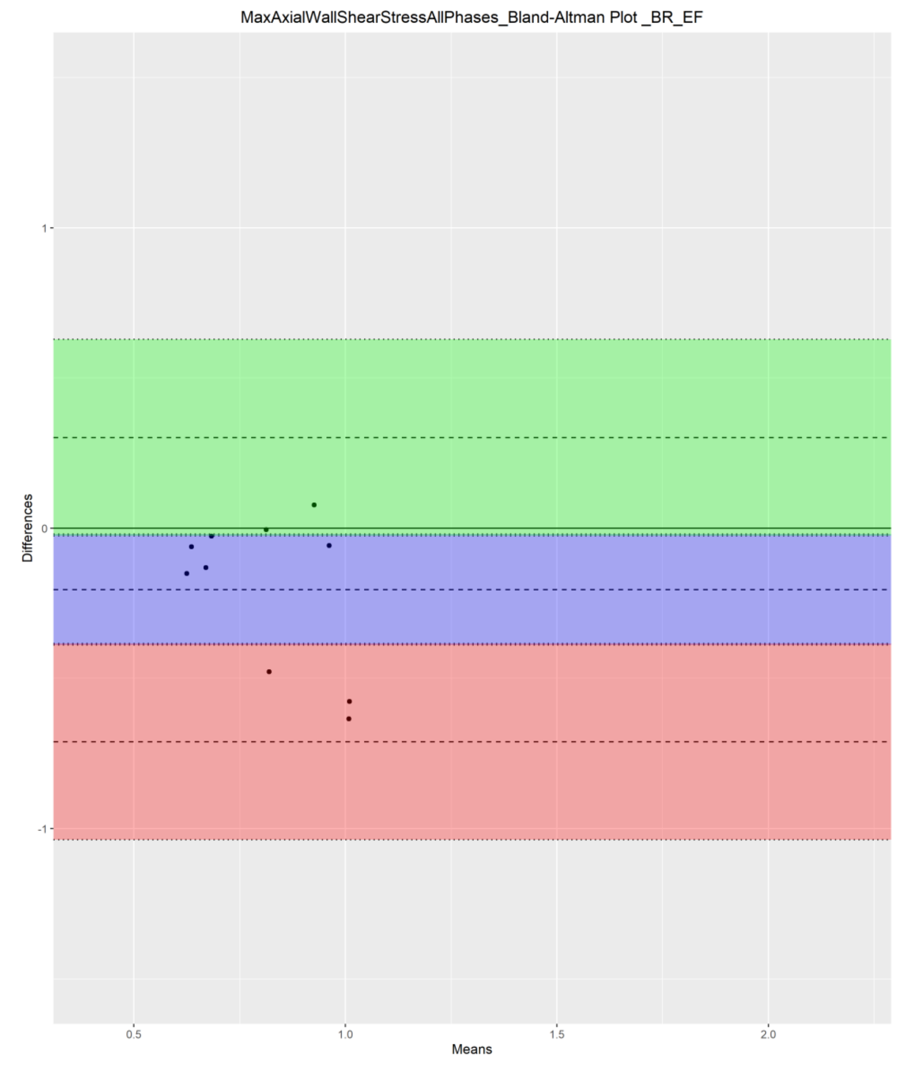

MaxCircumferentialWallShearStressAllPhases

Phi1.5T

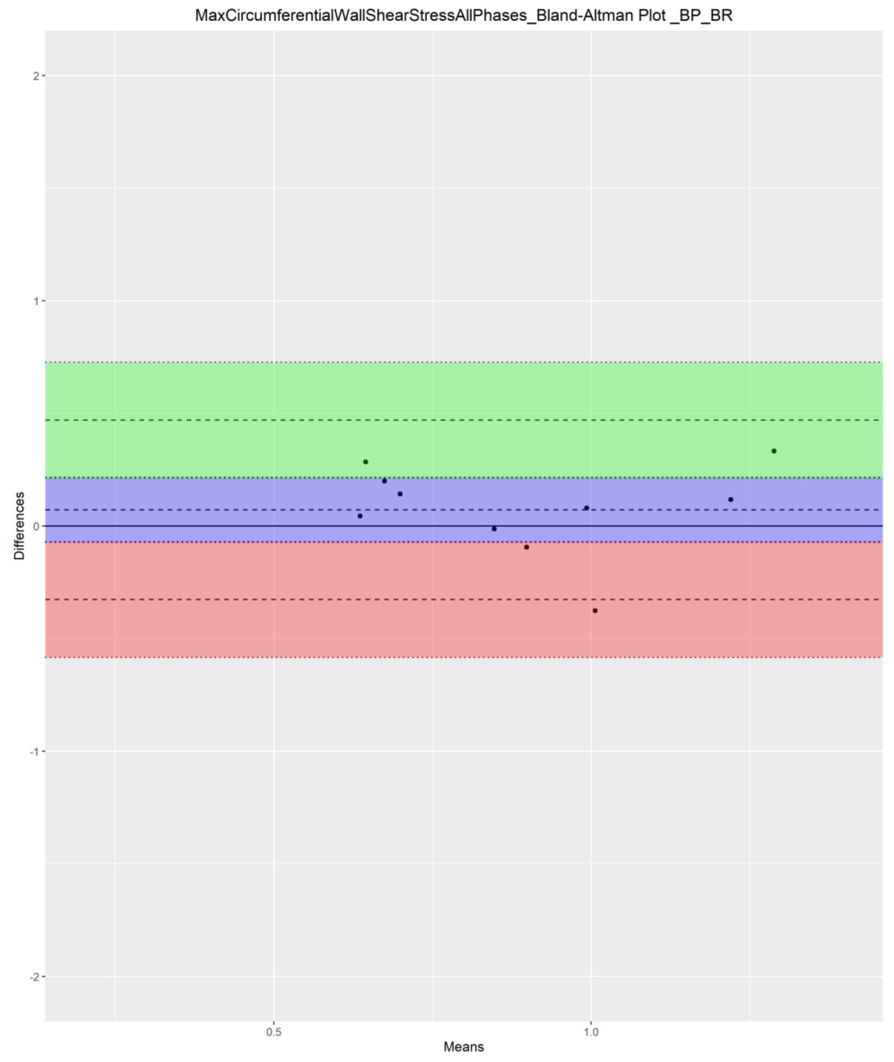

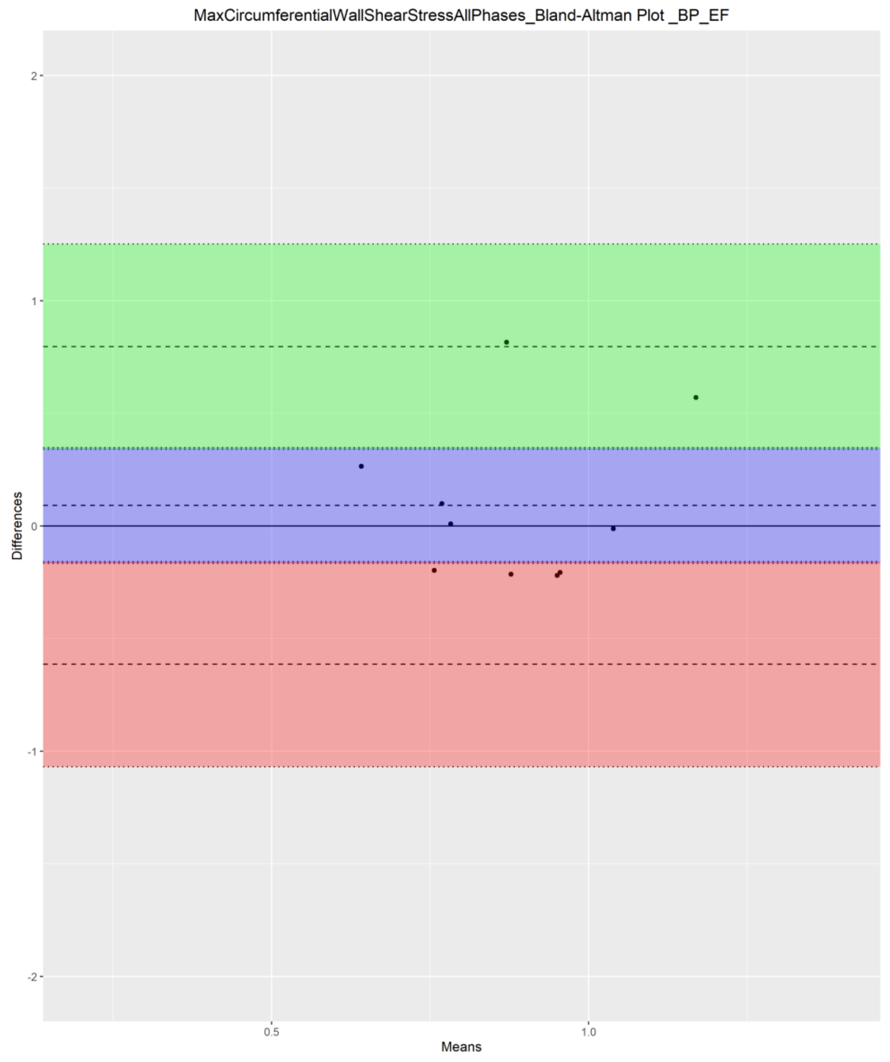

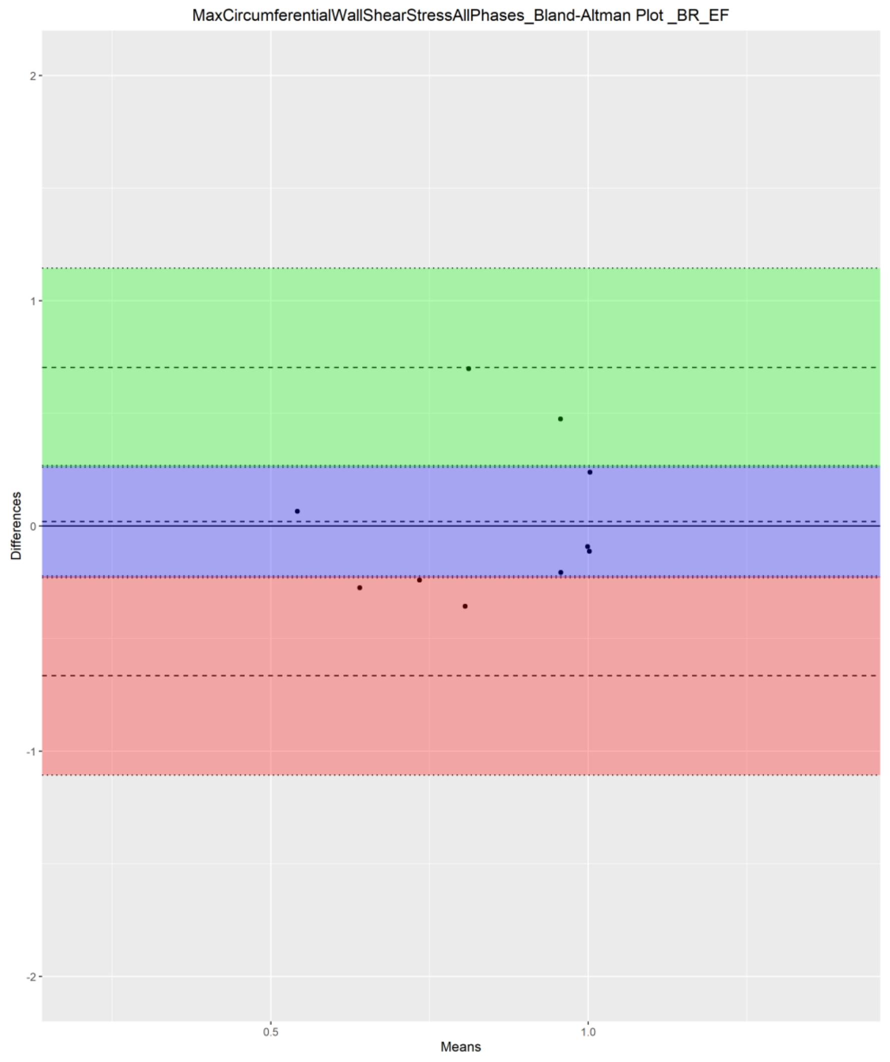

Phi3T

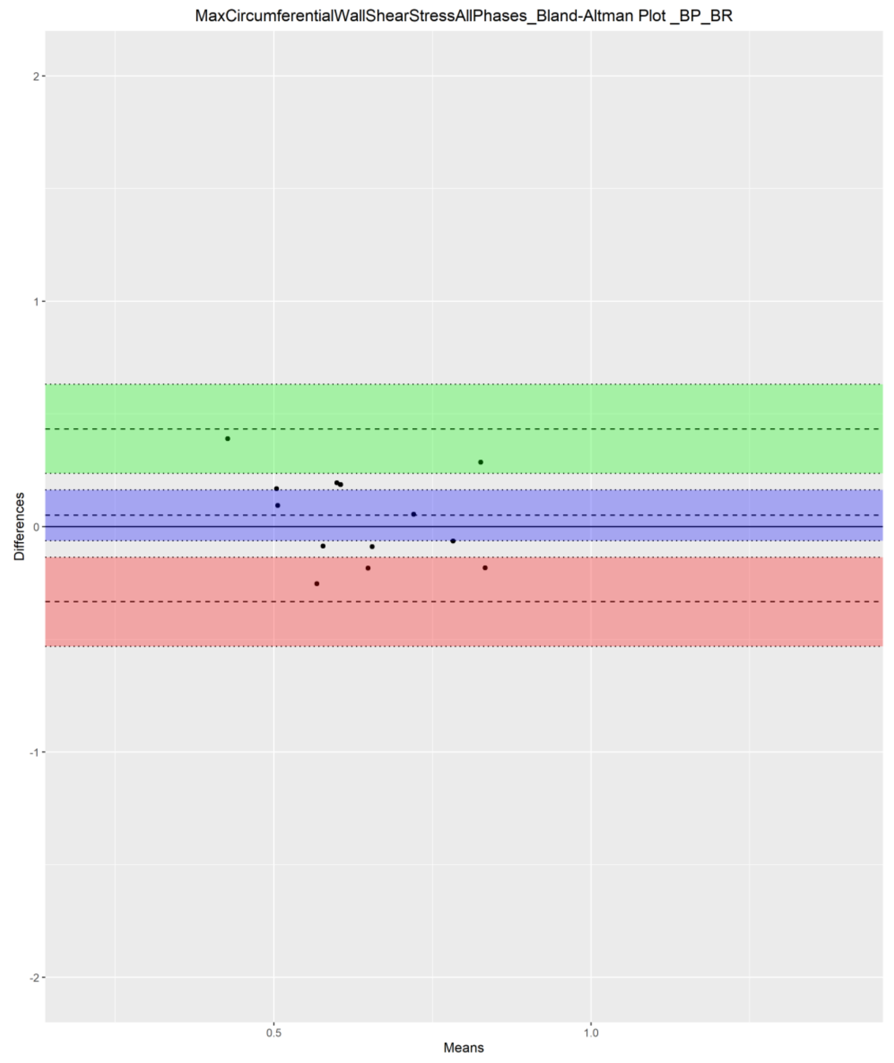

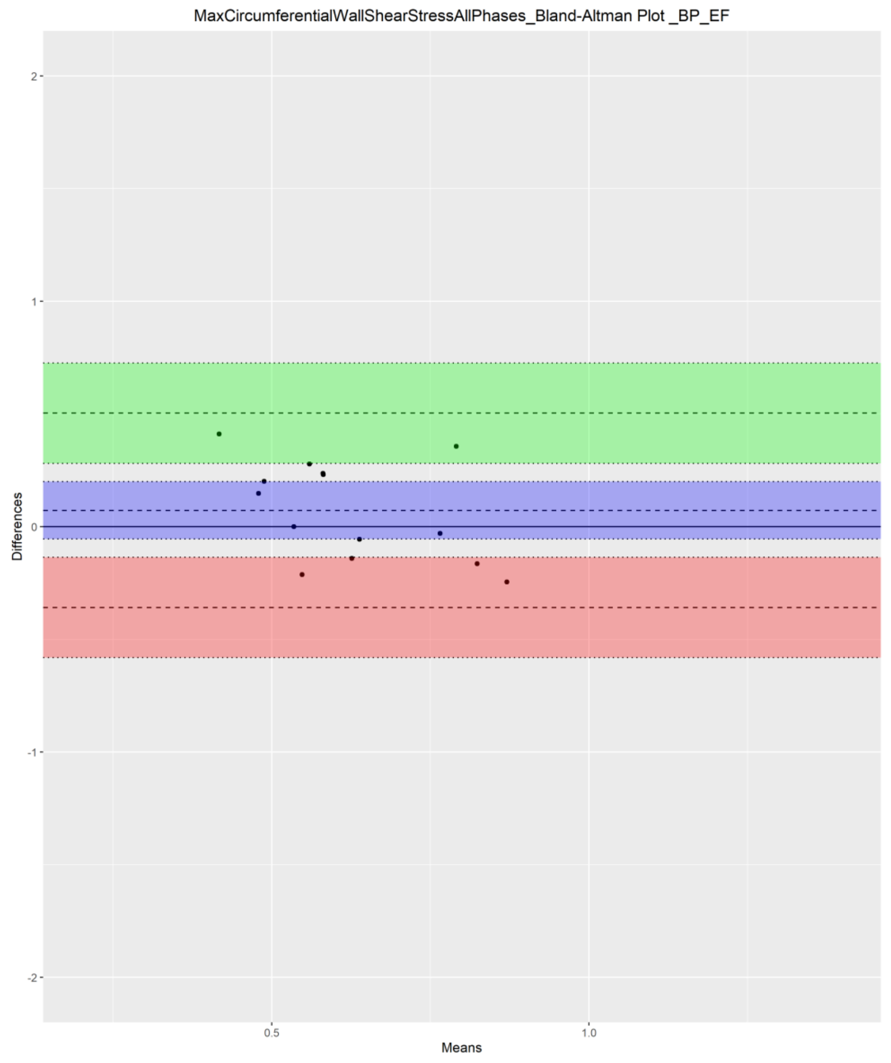

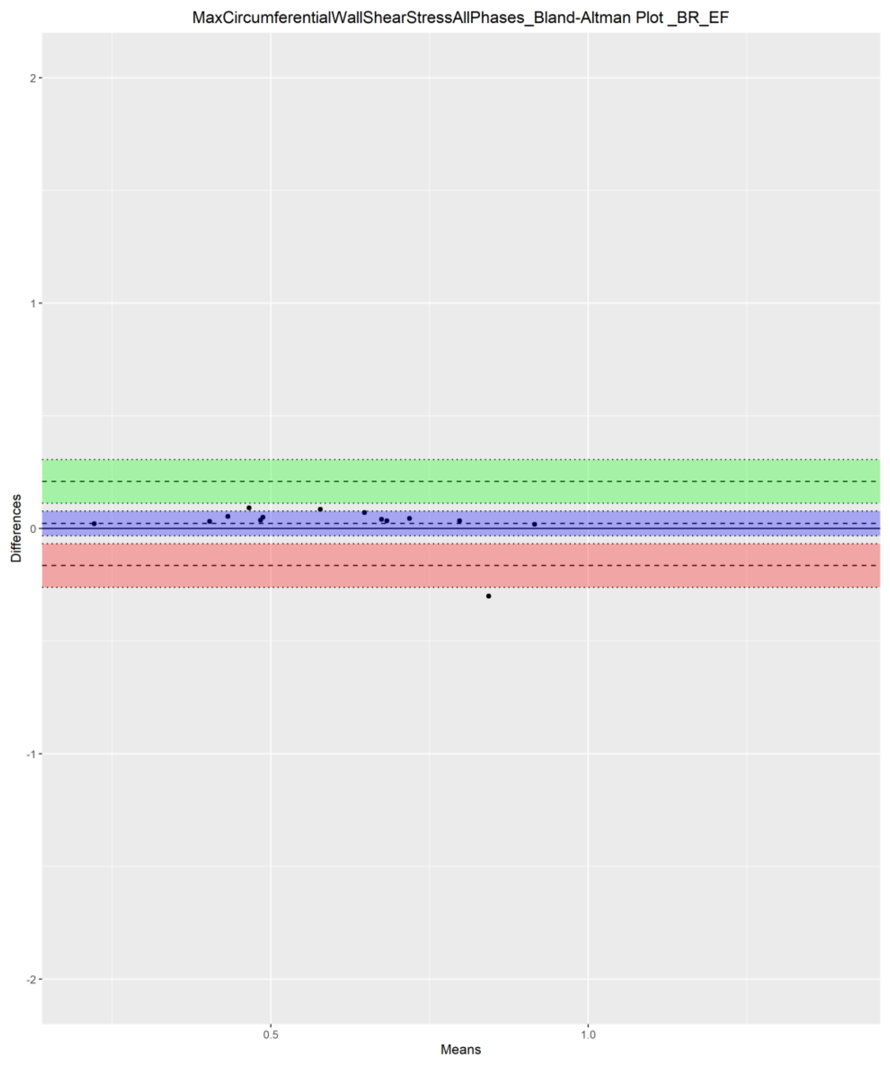

Siem3T

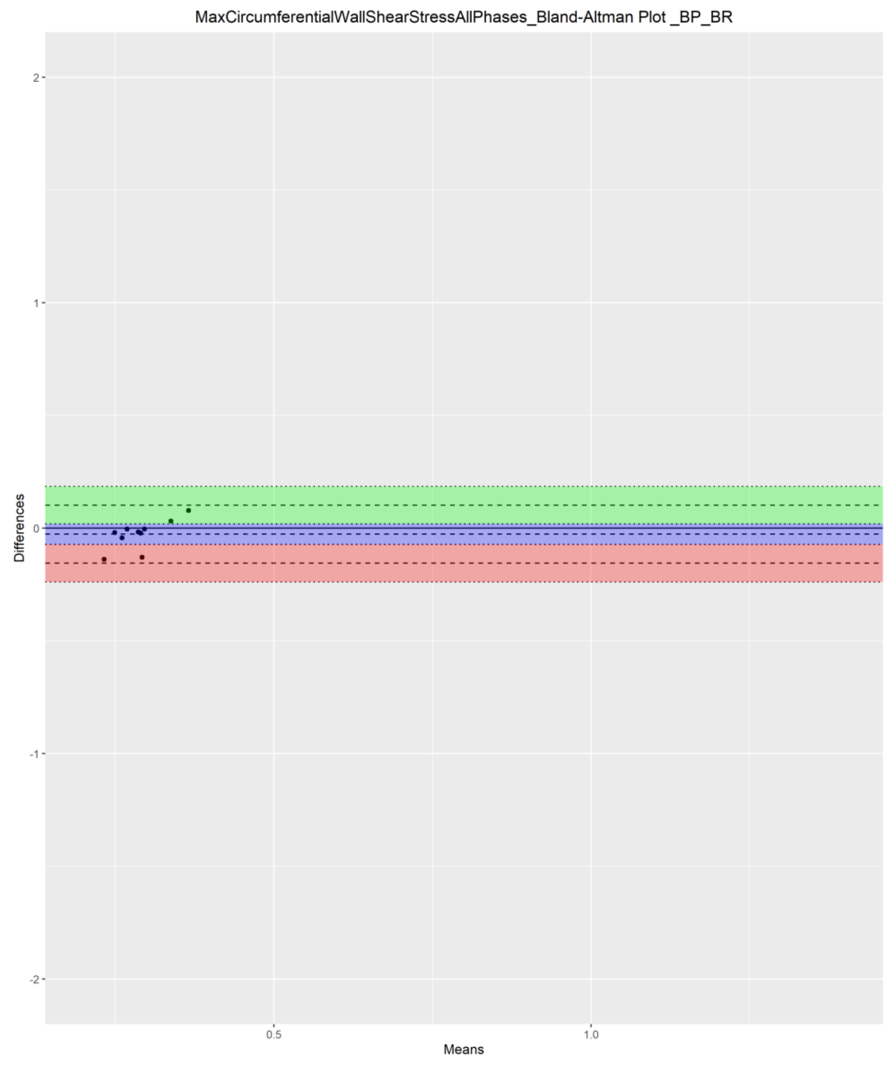

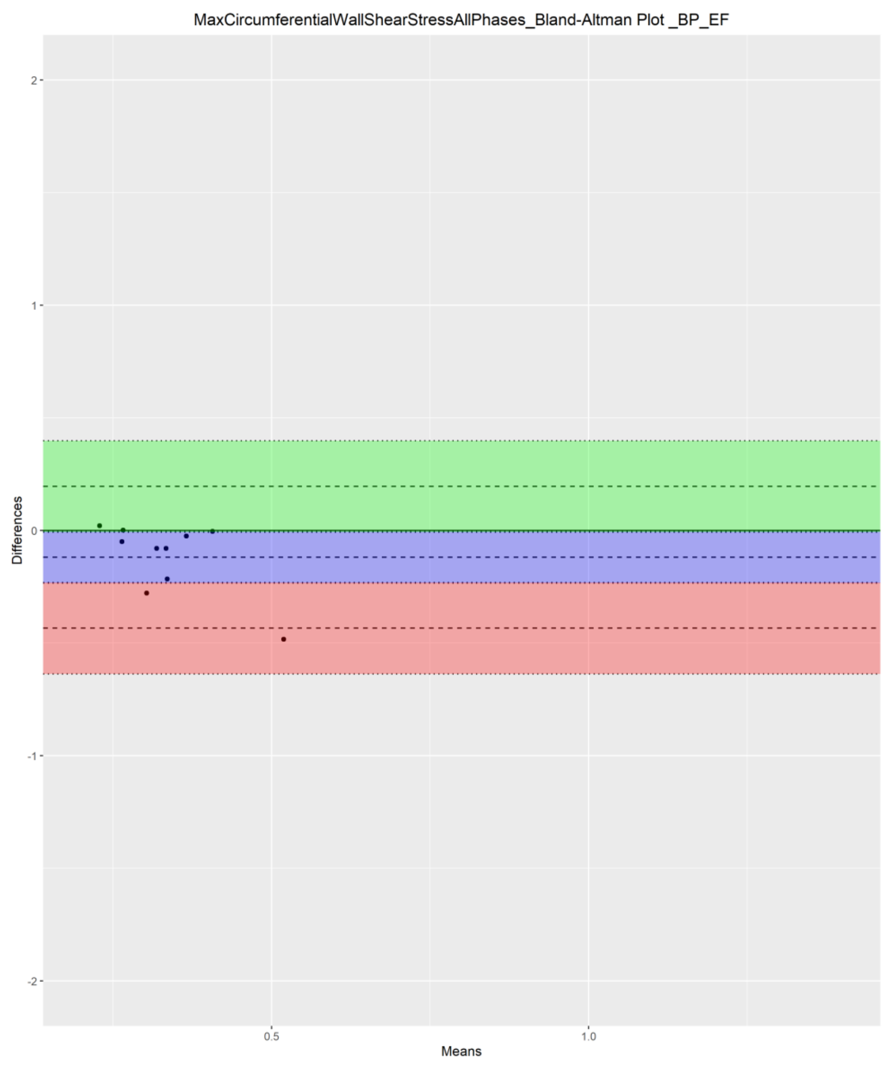

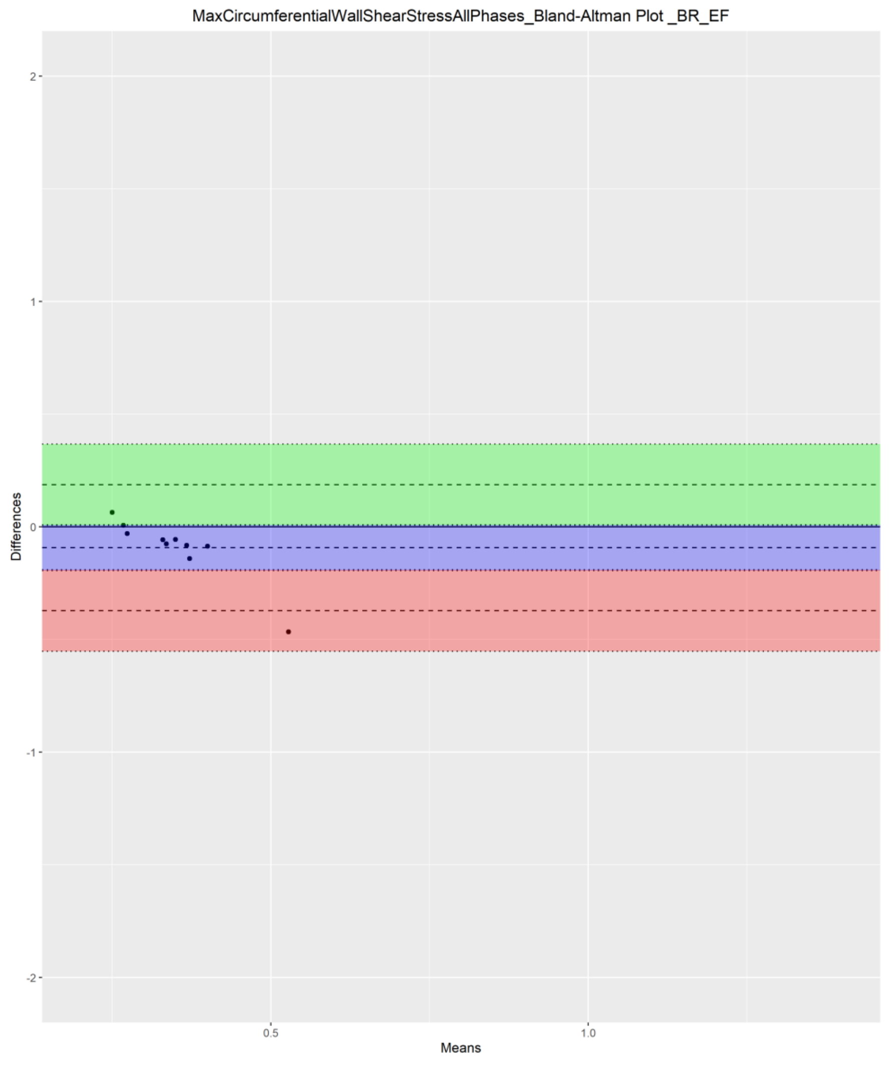

+ TAB ICC

MaximumFlow

Phi1.5T

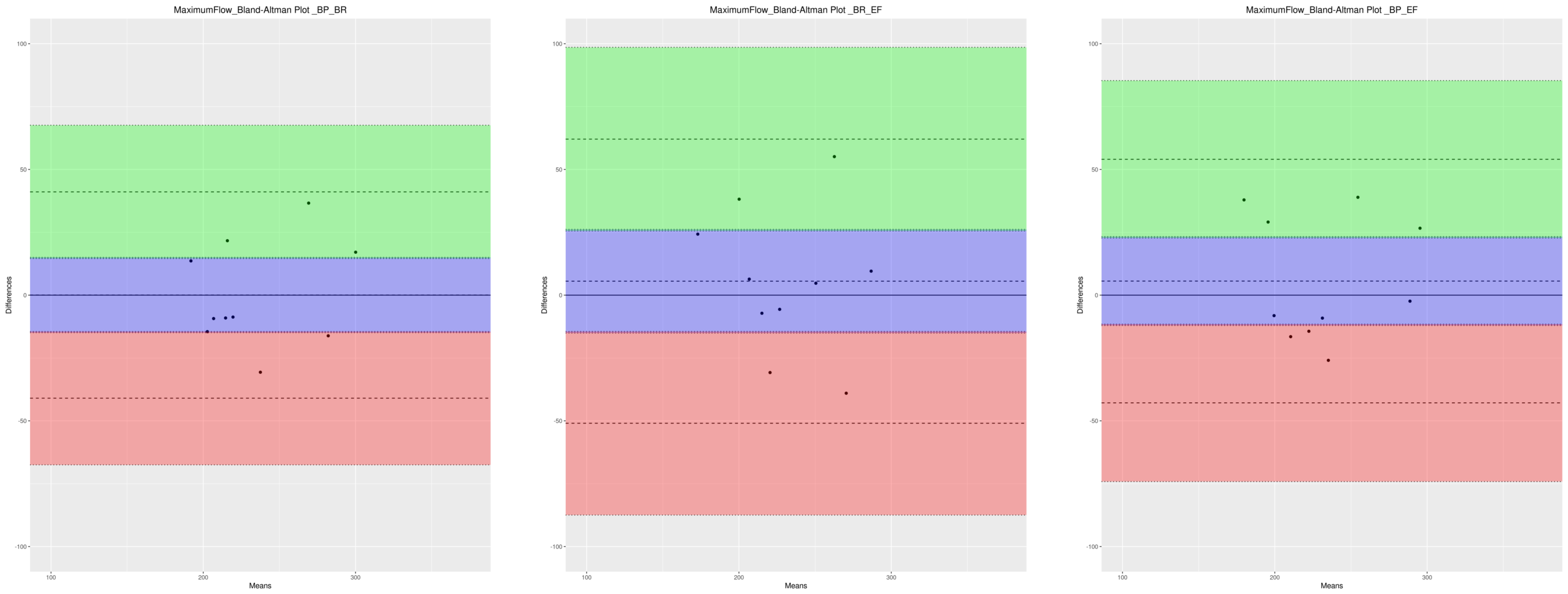

Phi3T

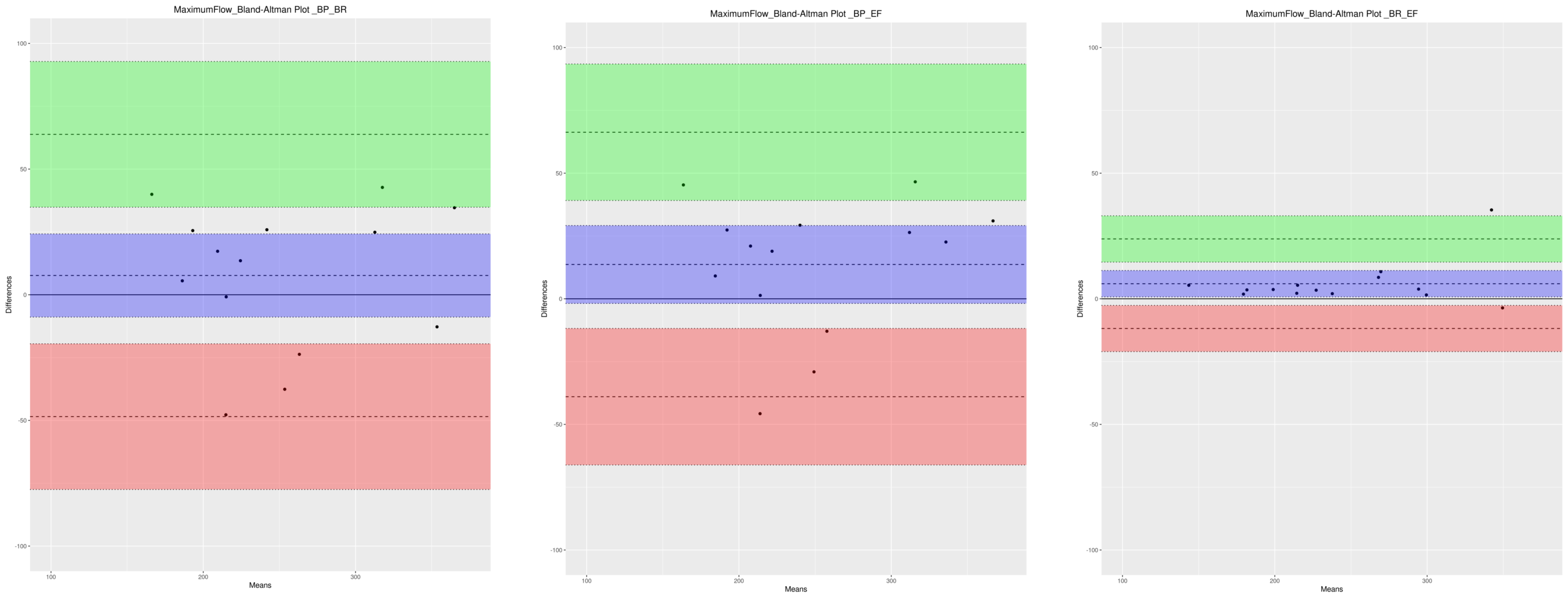

Siem3T

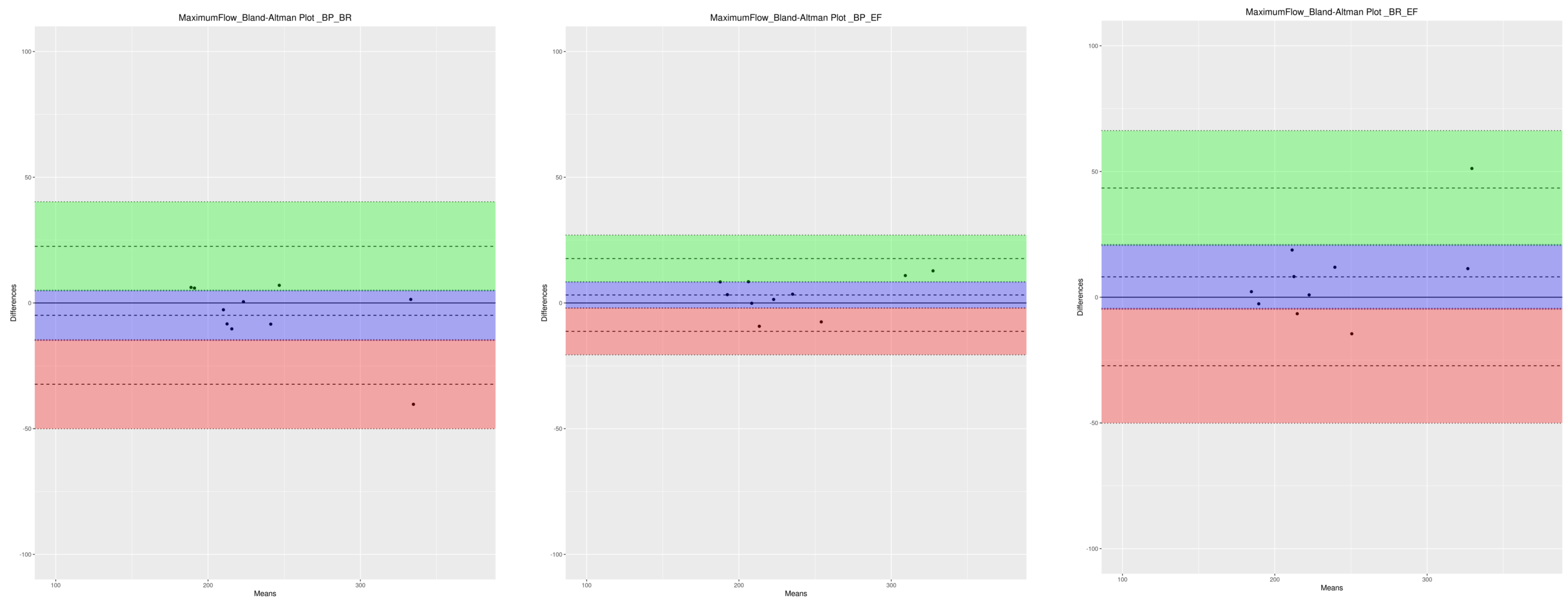

MaximumMeanVelocity

Phi1.5T

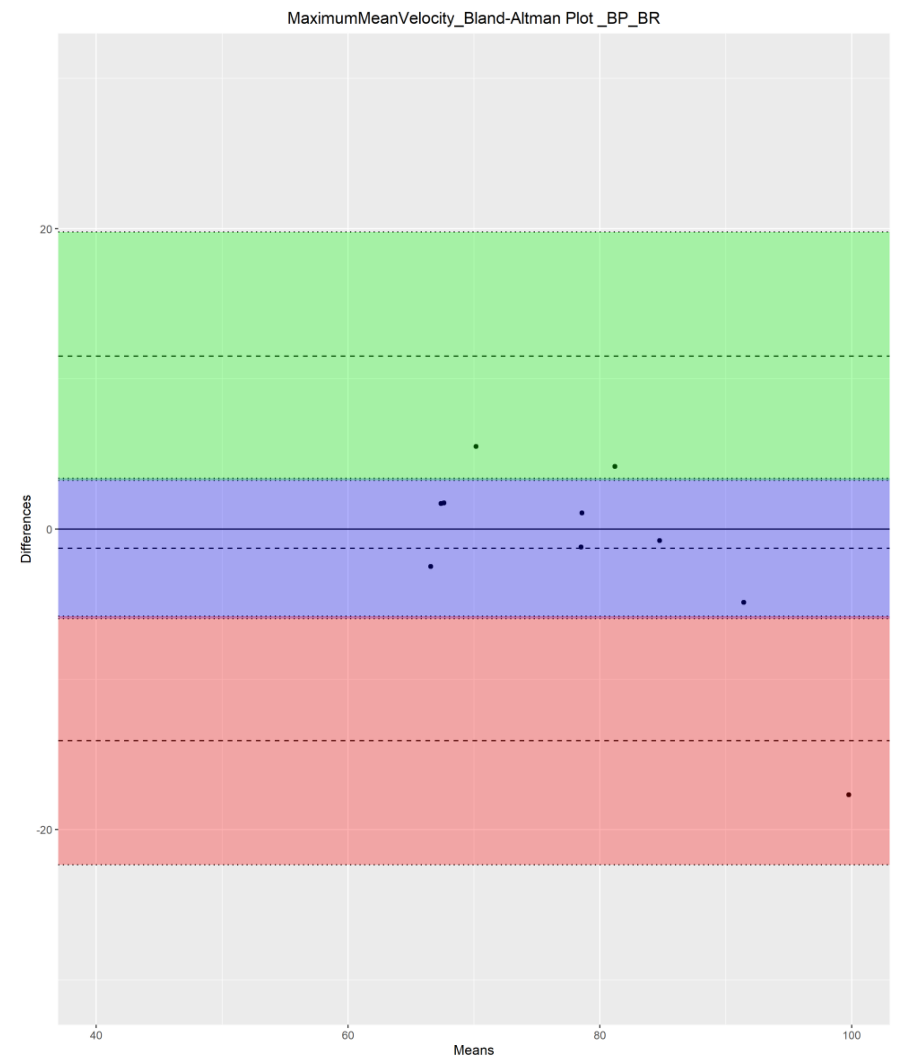

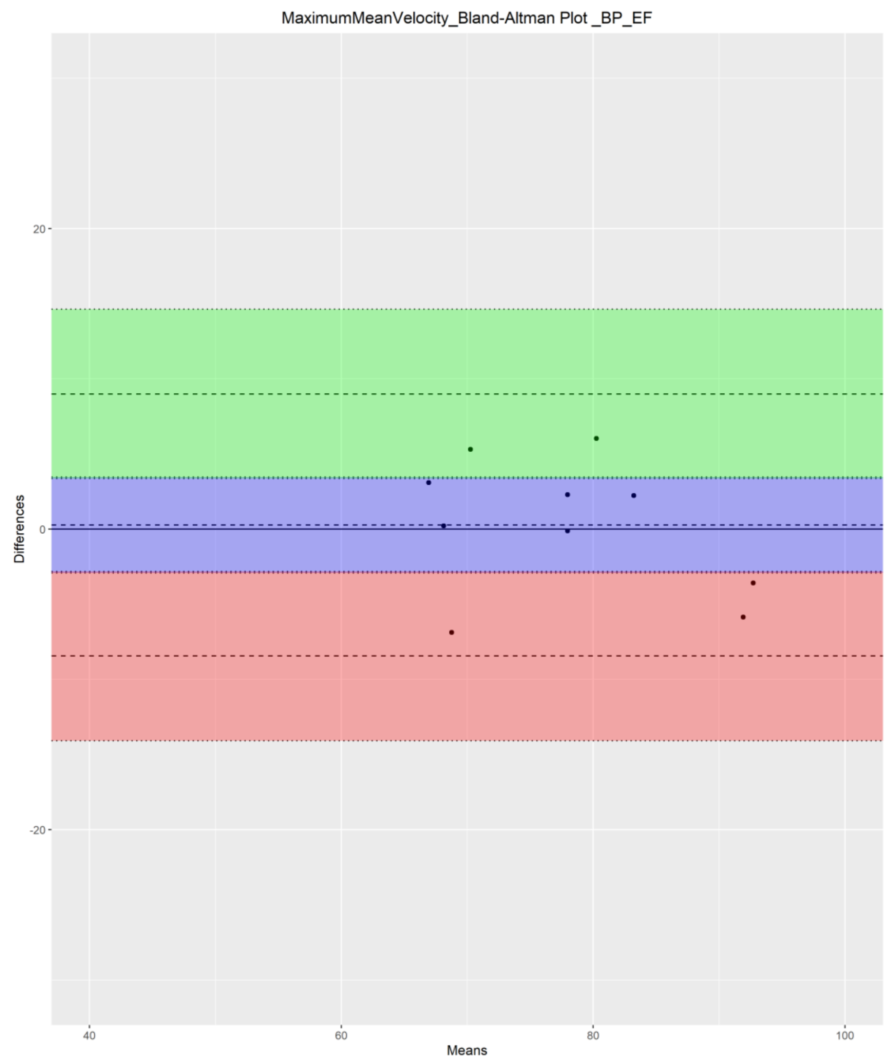

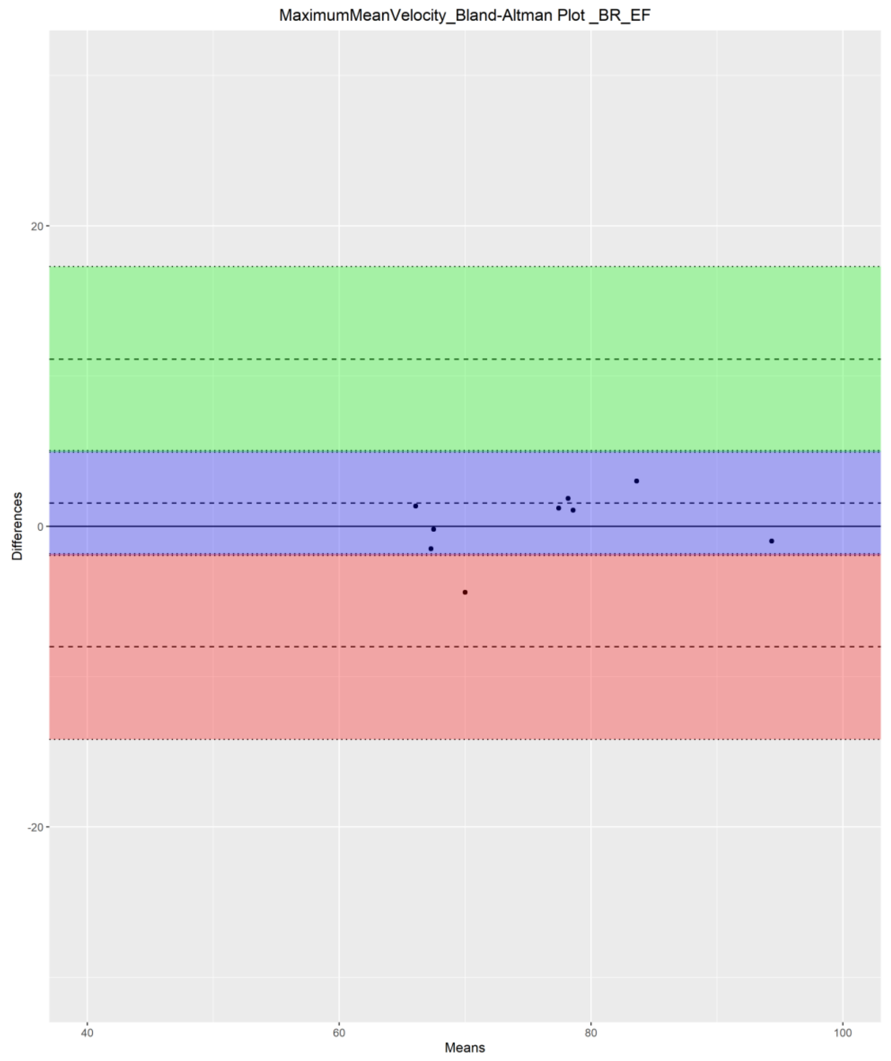

Phi3T

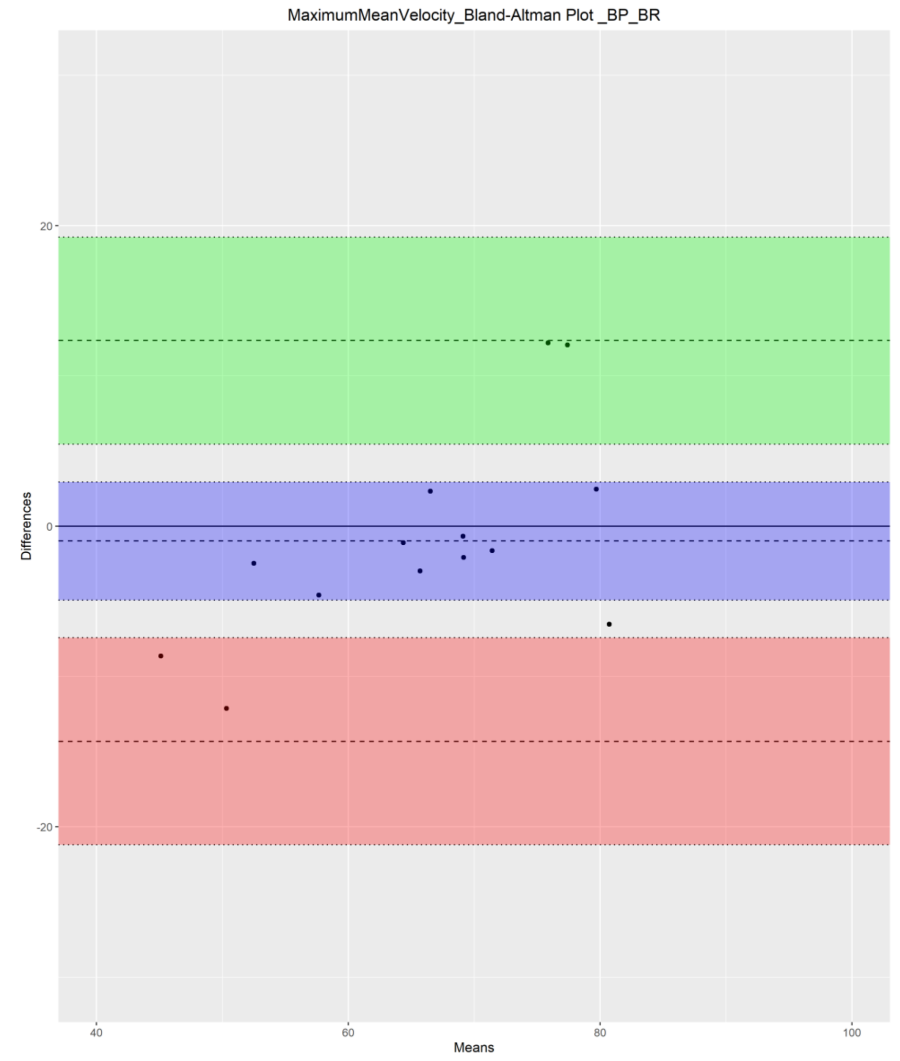

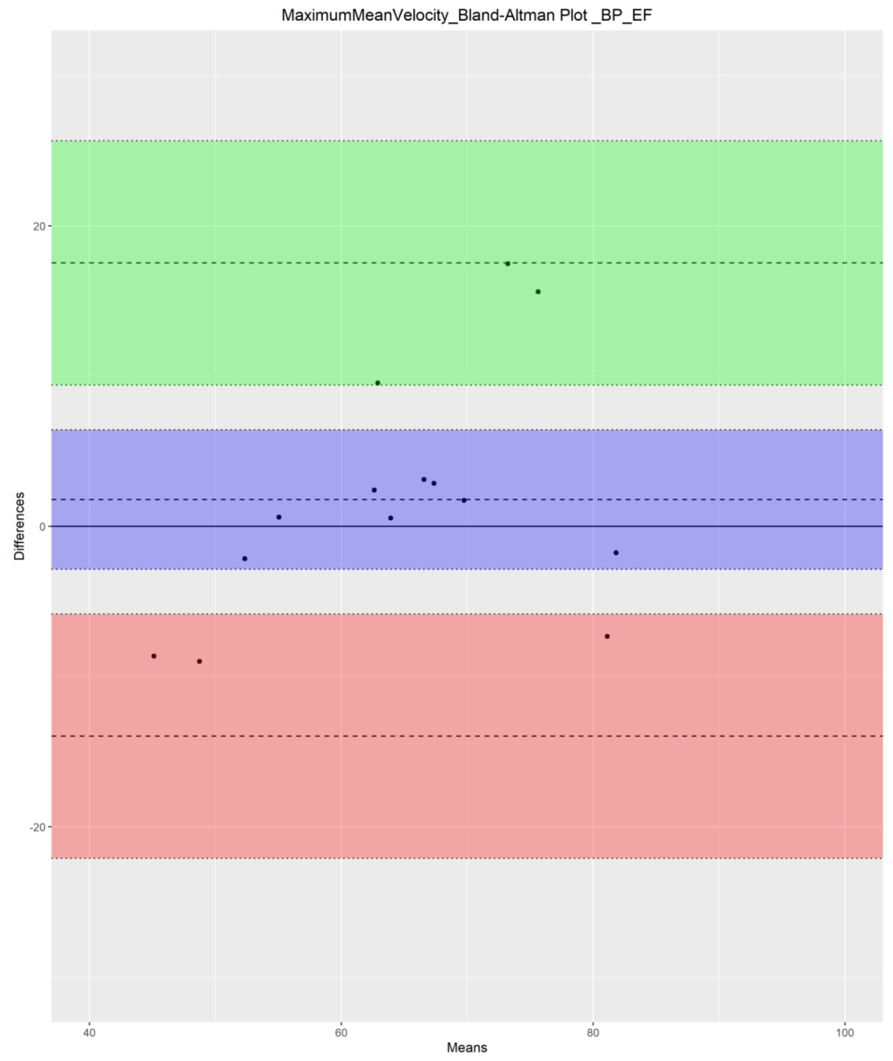

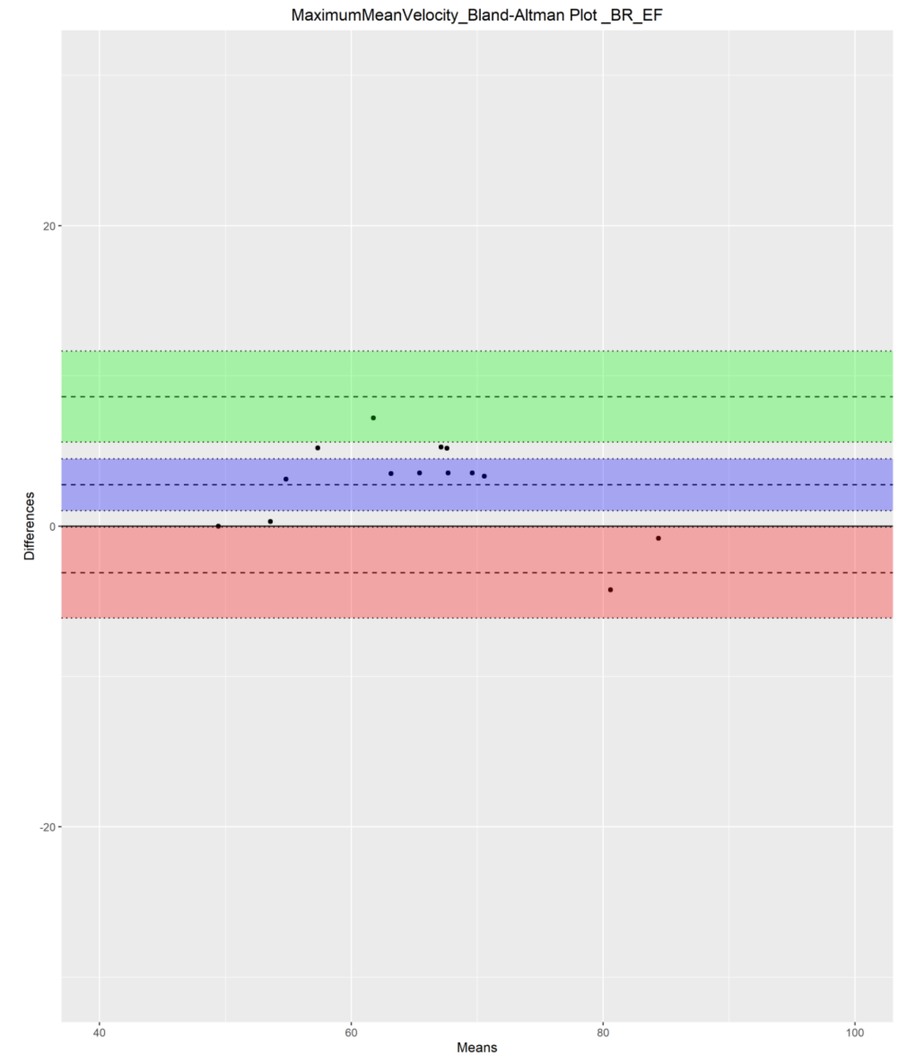

Siem3T

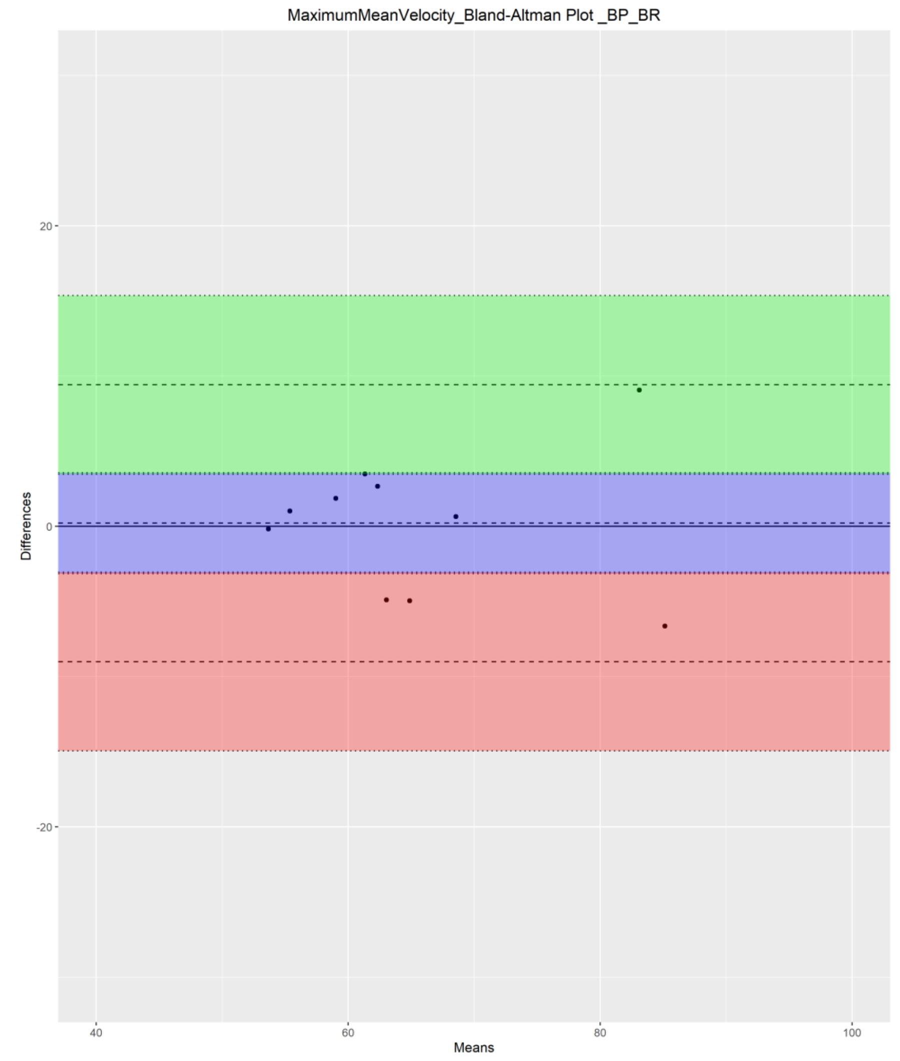

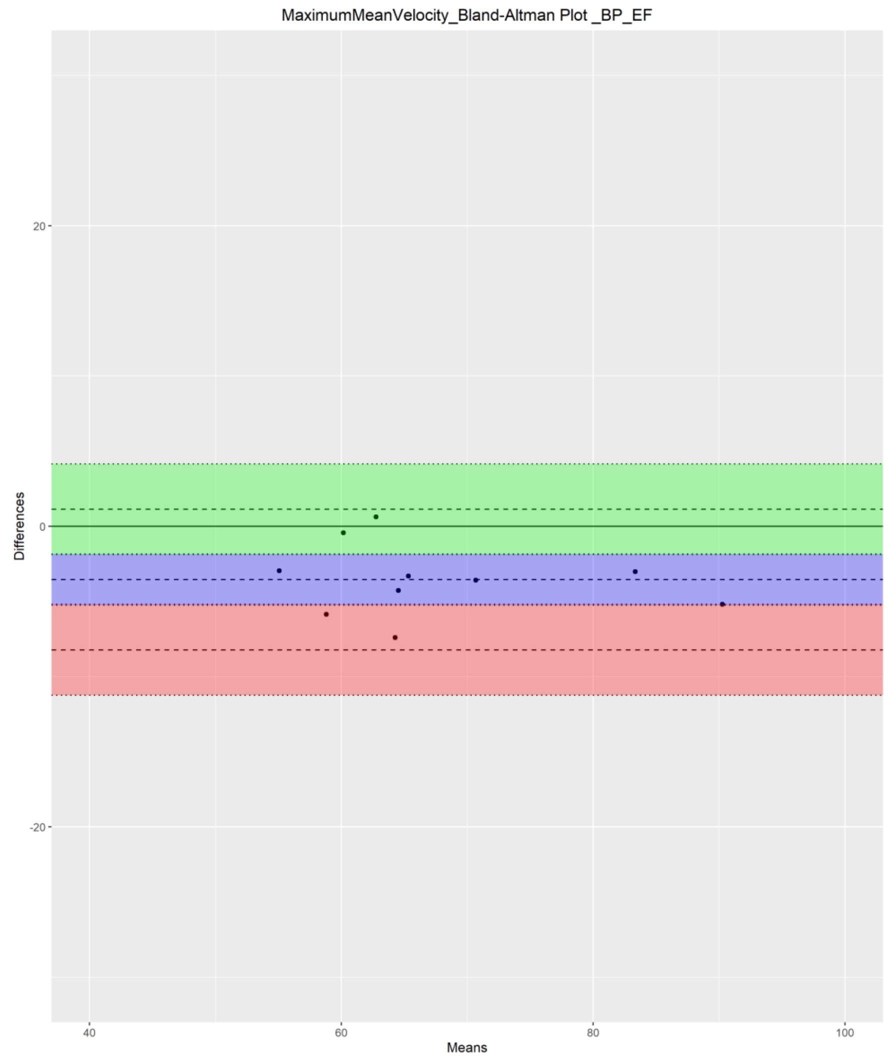

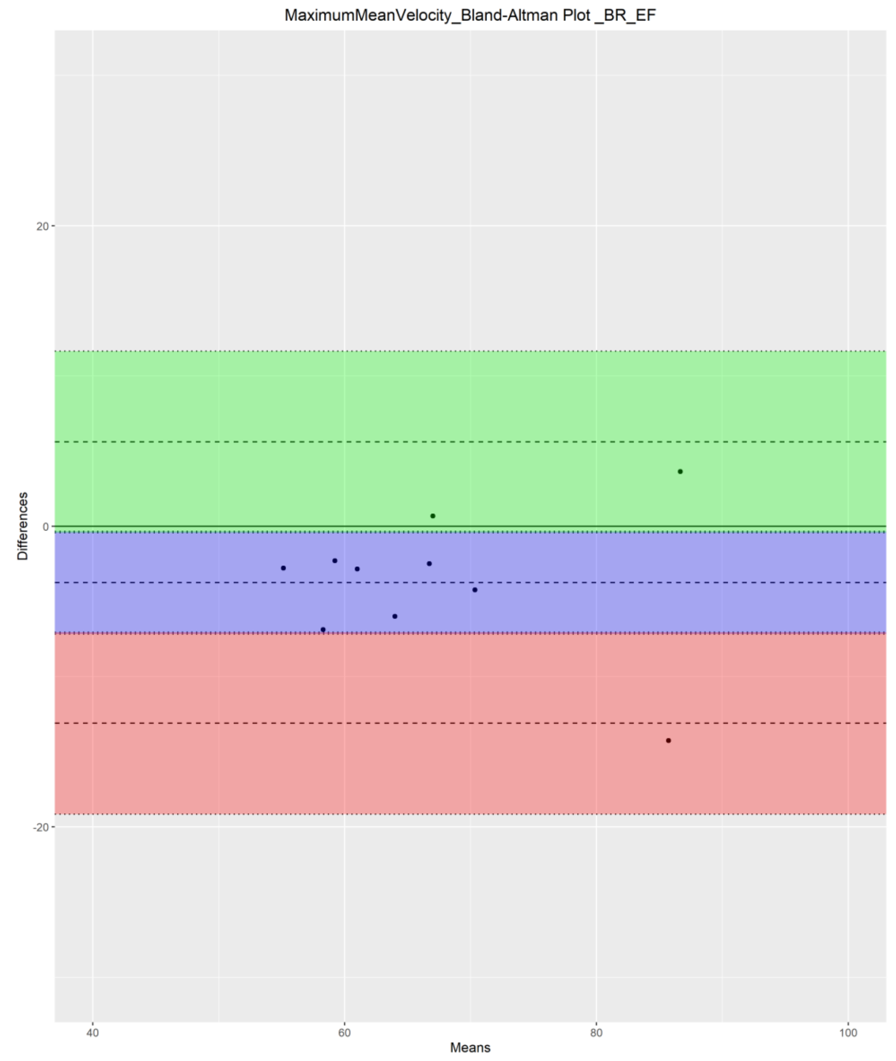

MaxPressureGradient

Phi1.5T

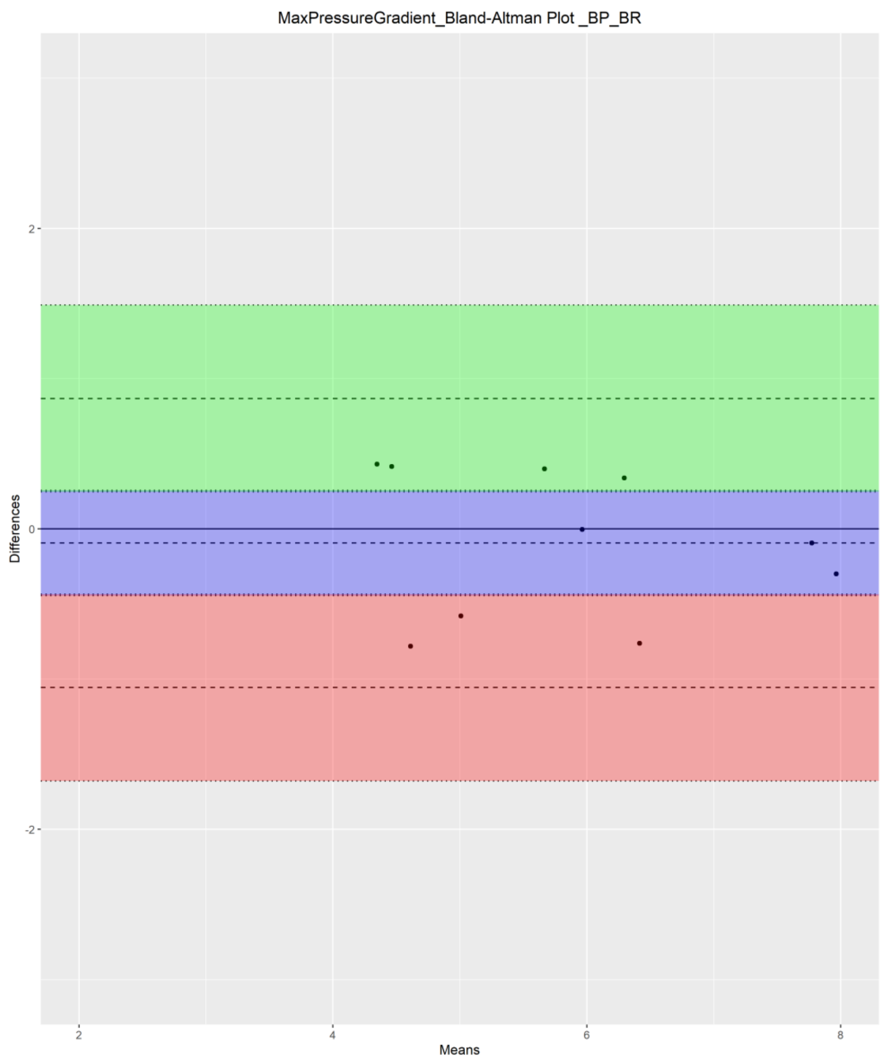

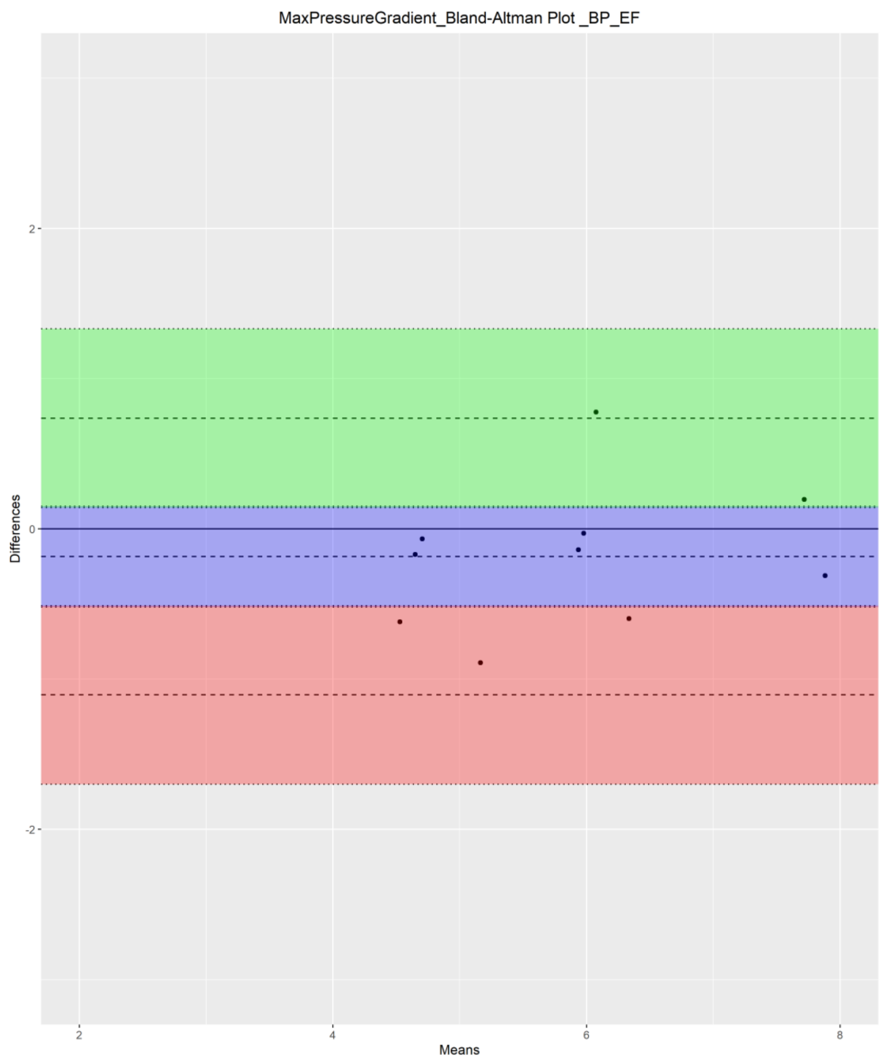

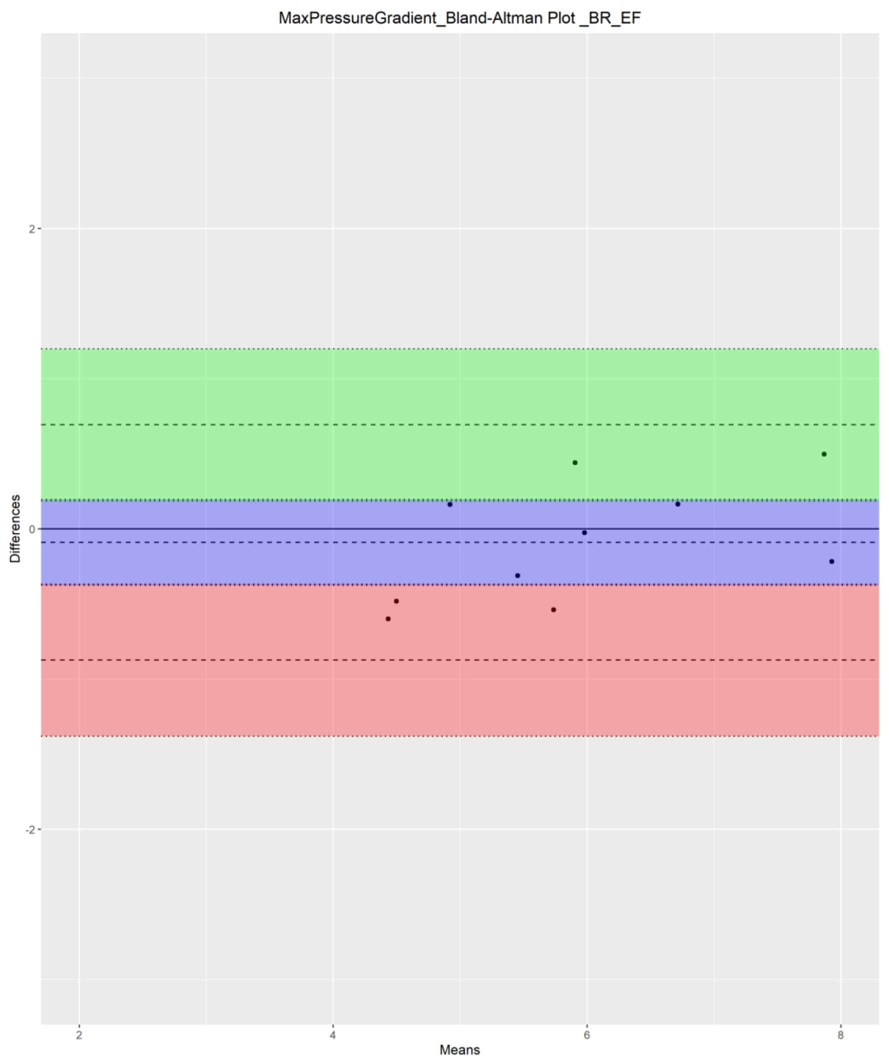

Phi3T

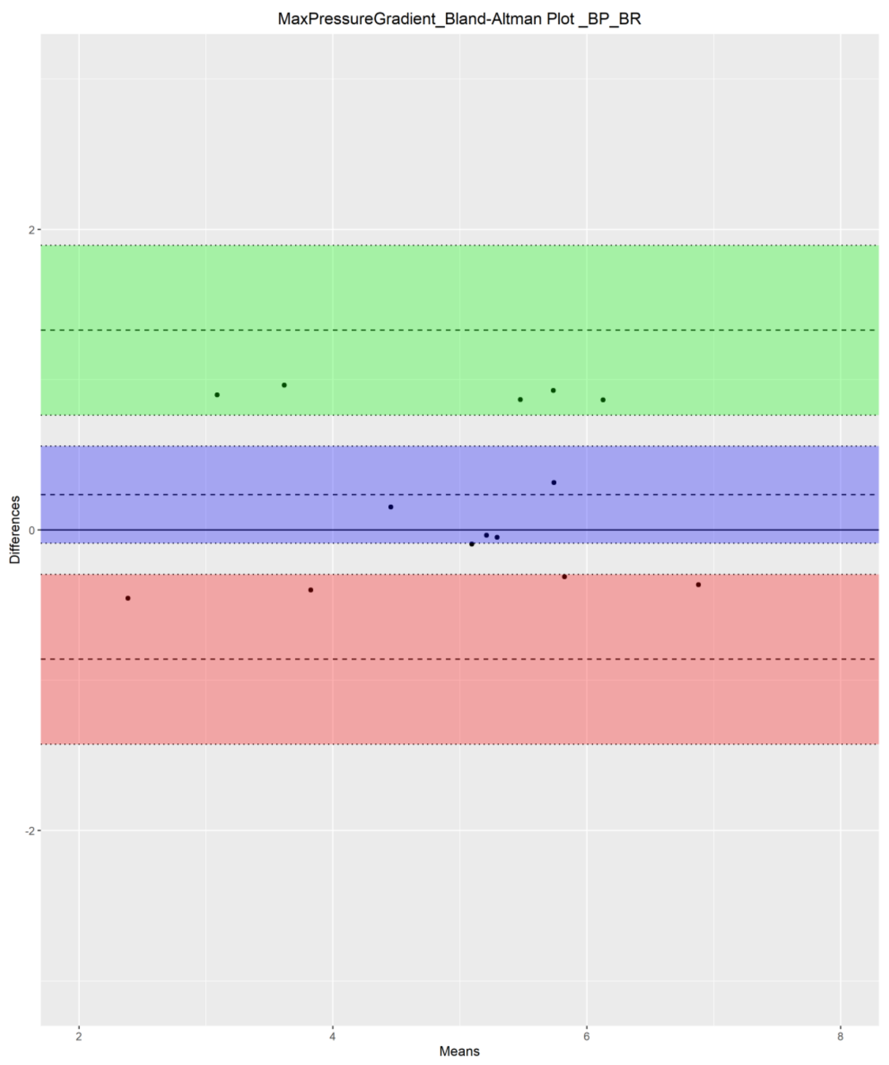

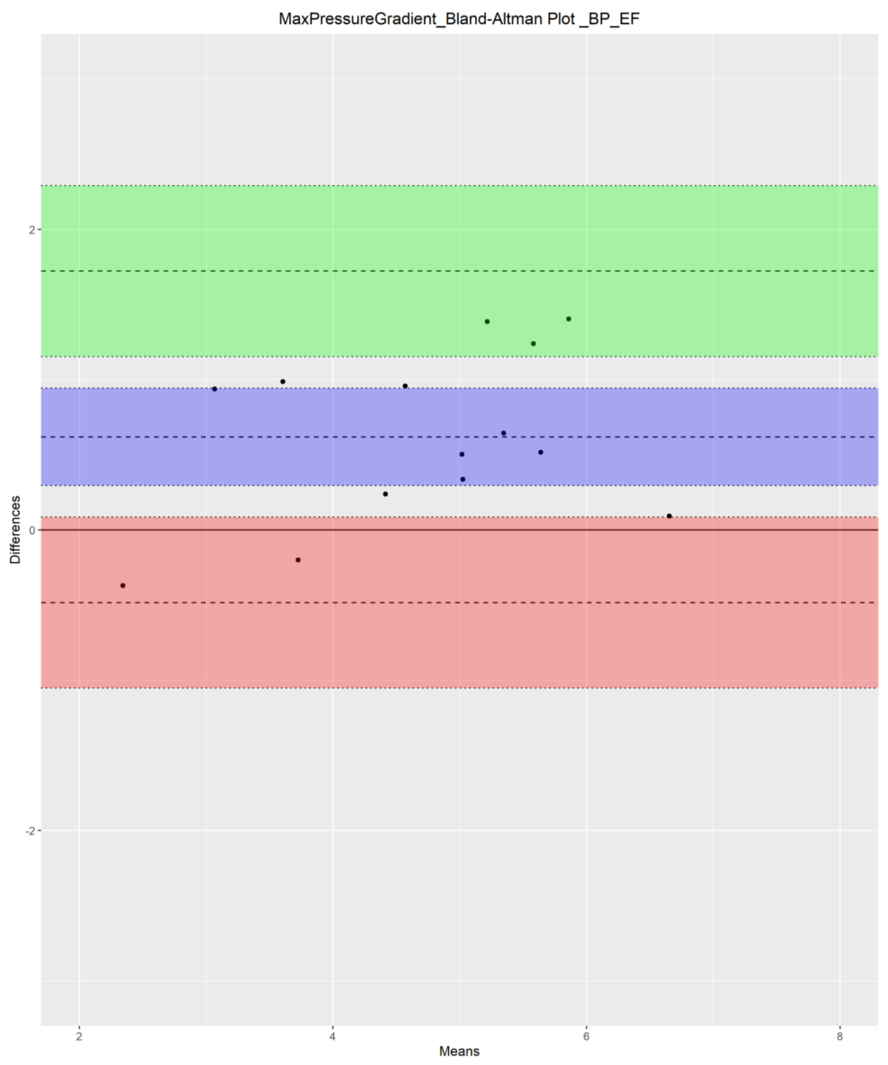

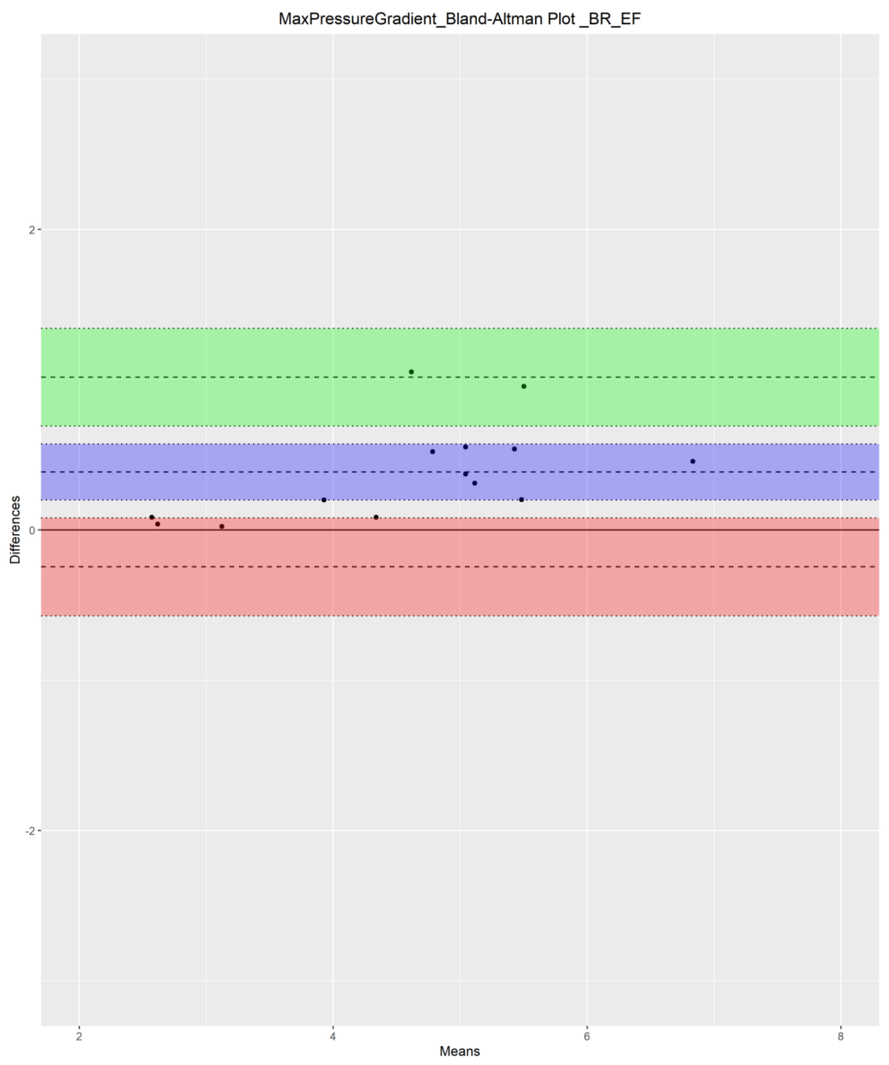

Siem3T

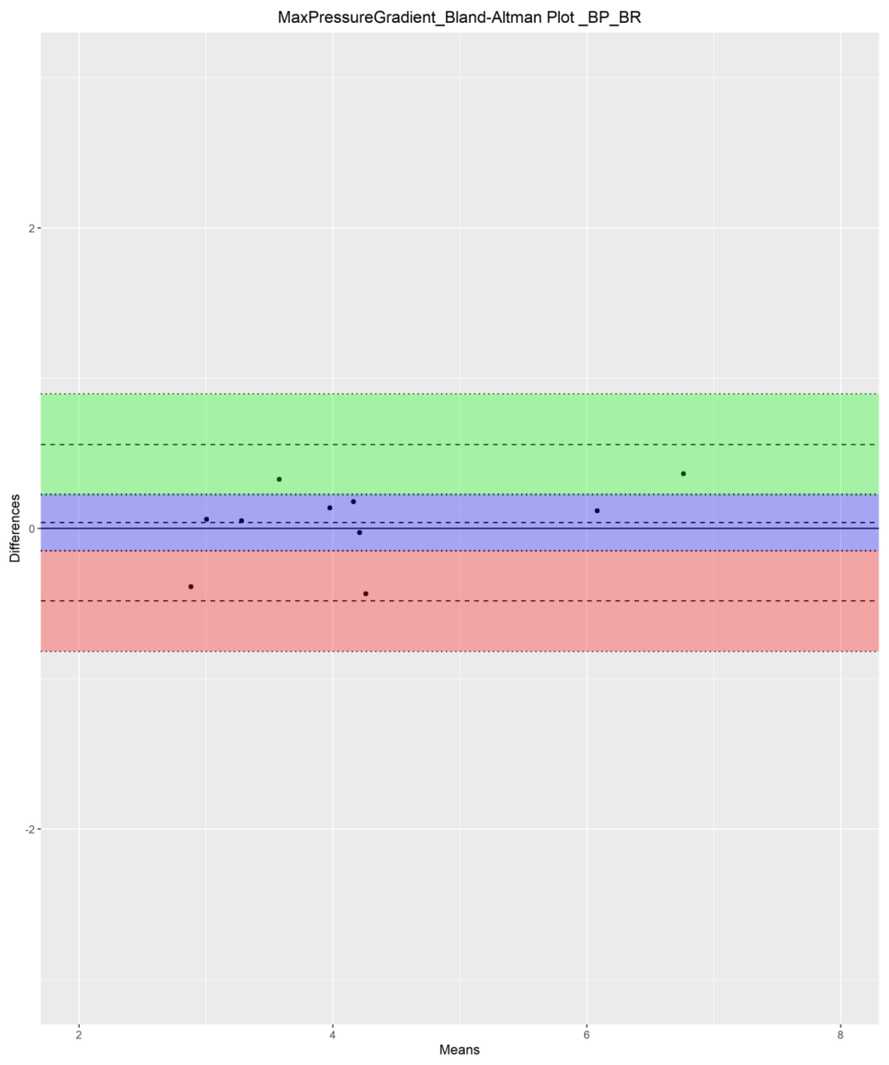

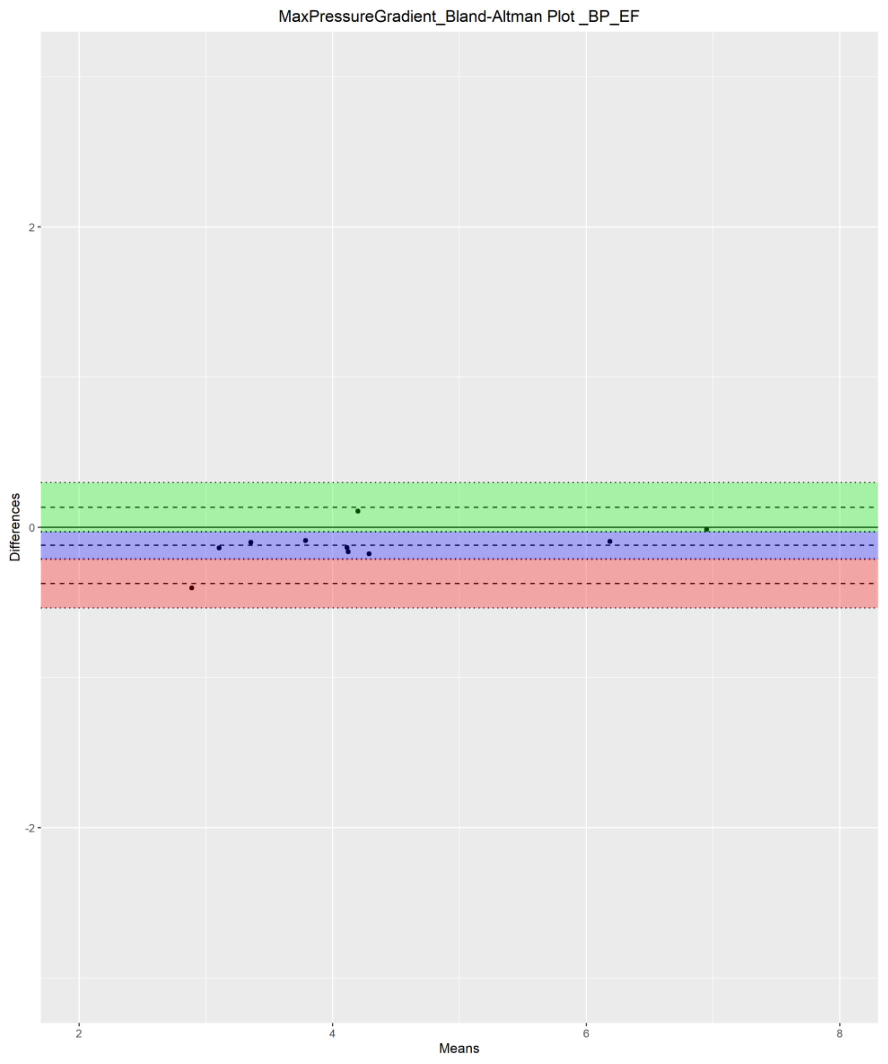

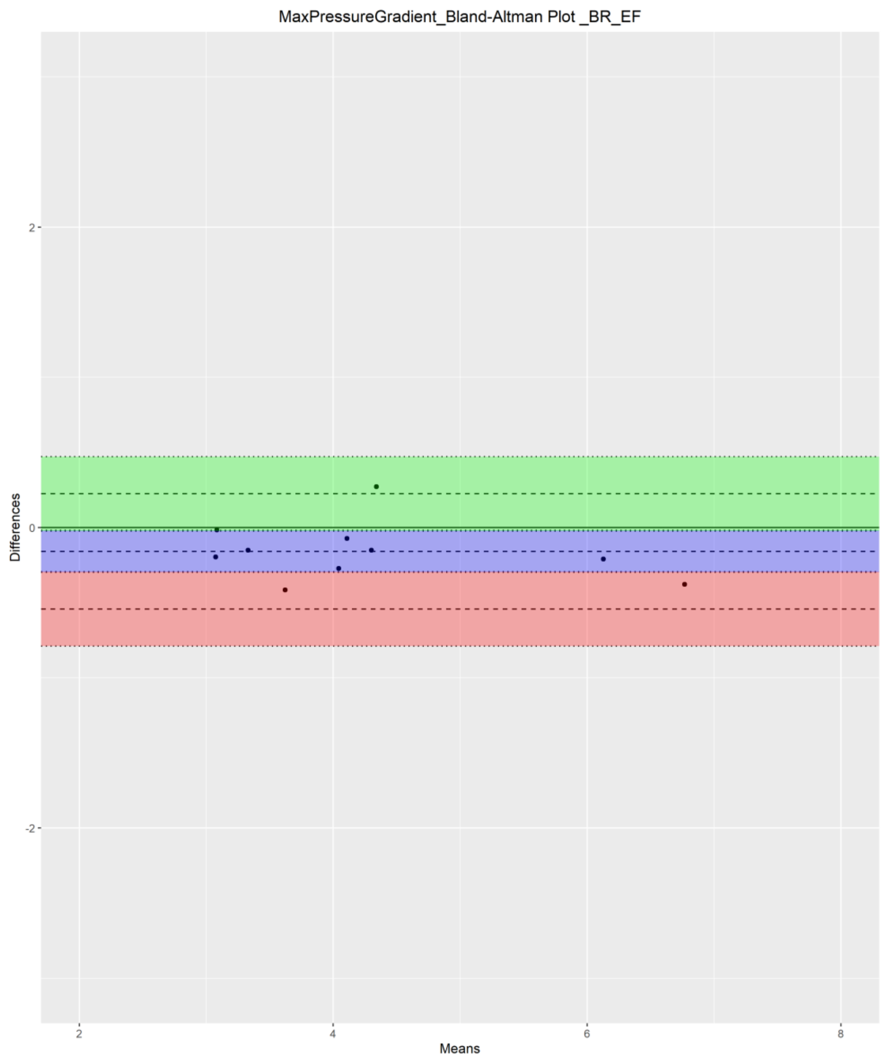

MeanPressureGradient

Phi1.5T

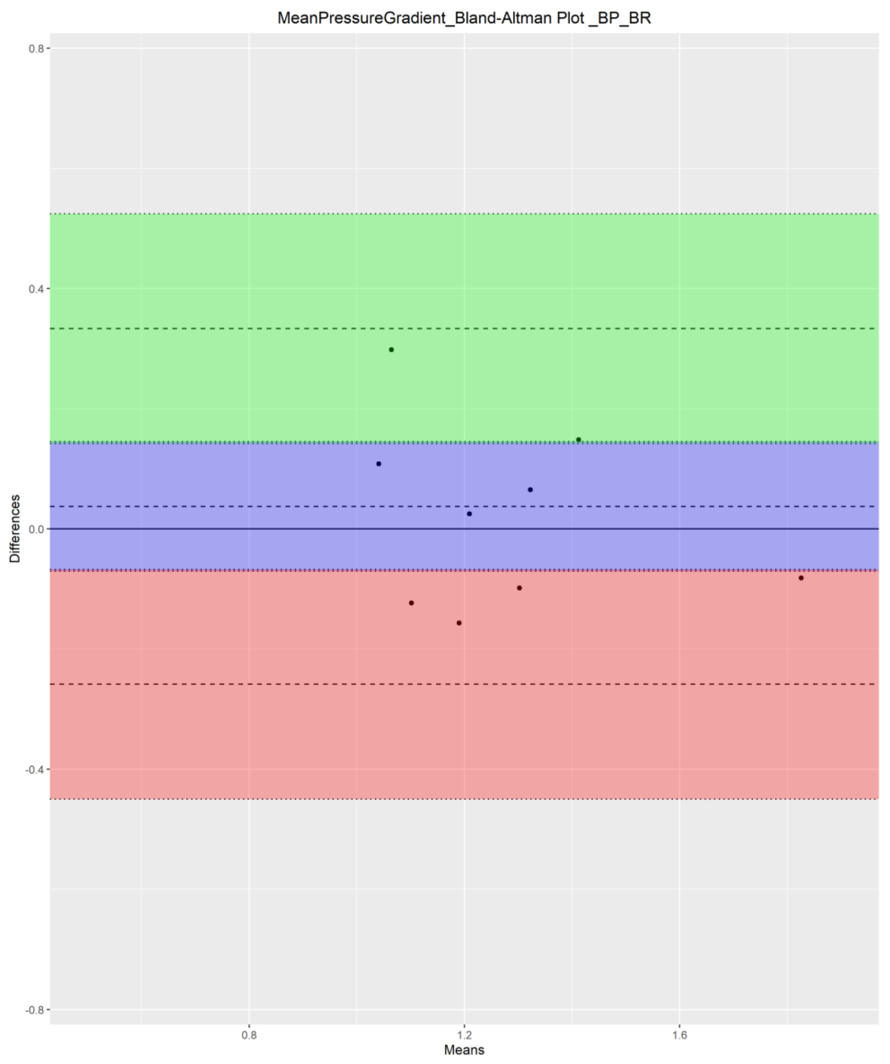

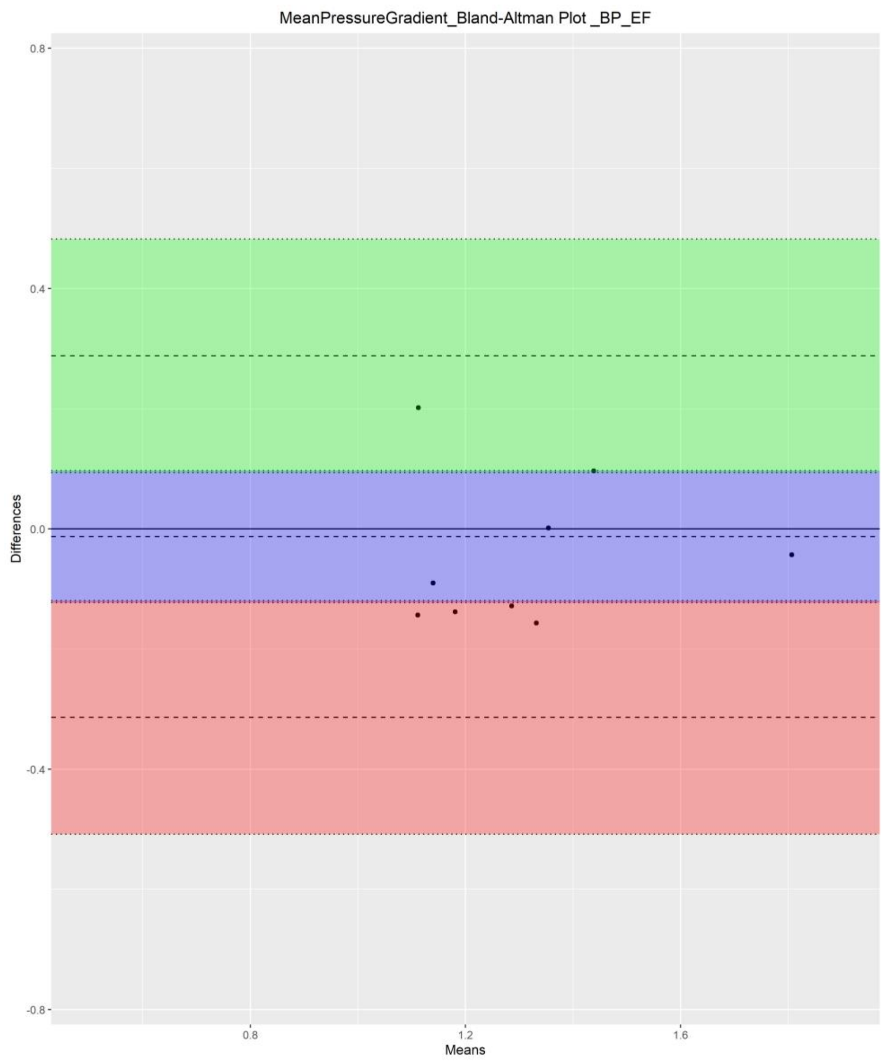

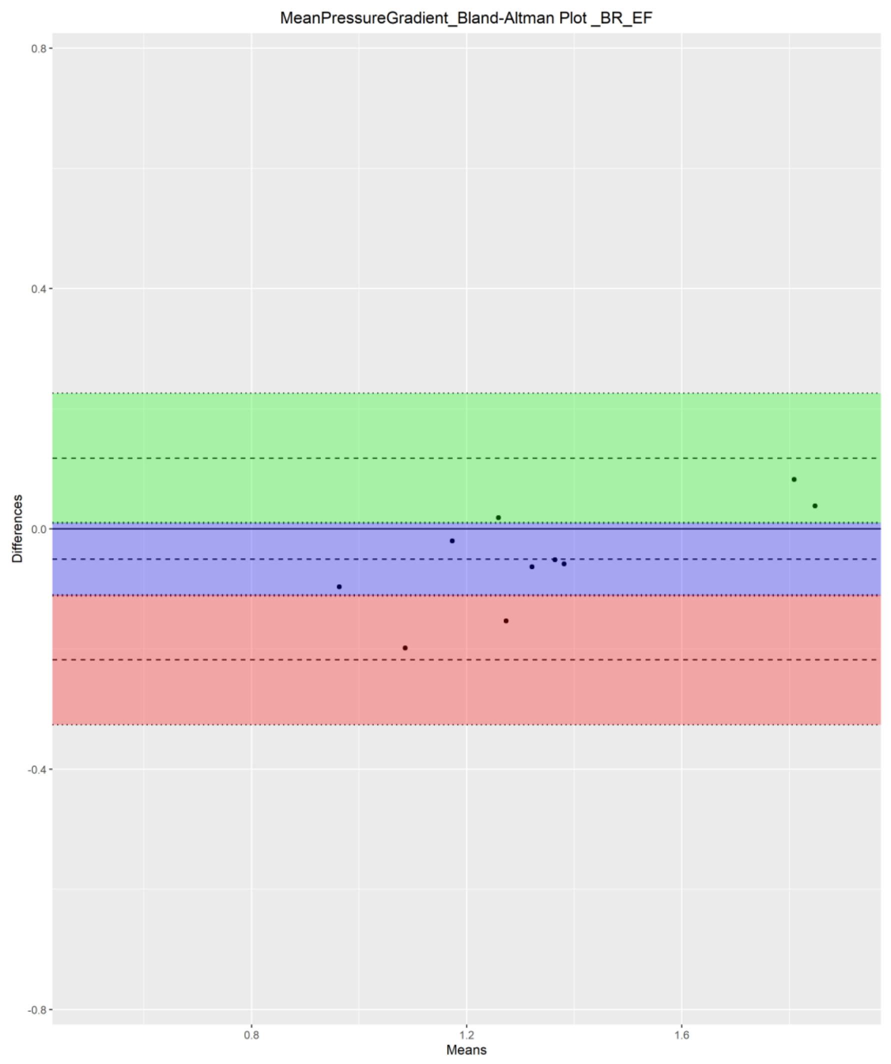

Phi3T

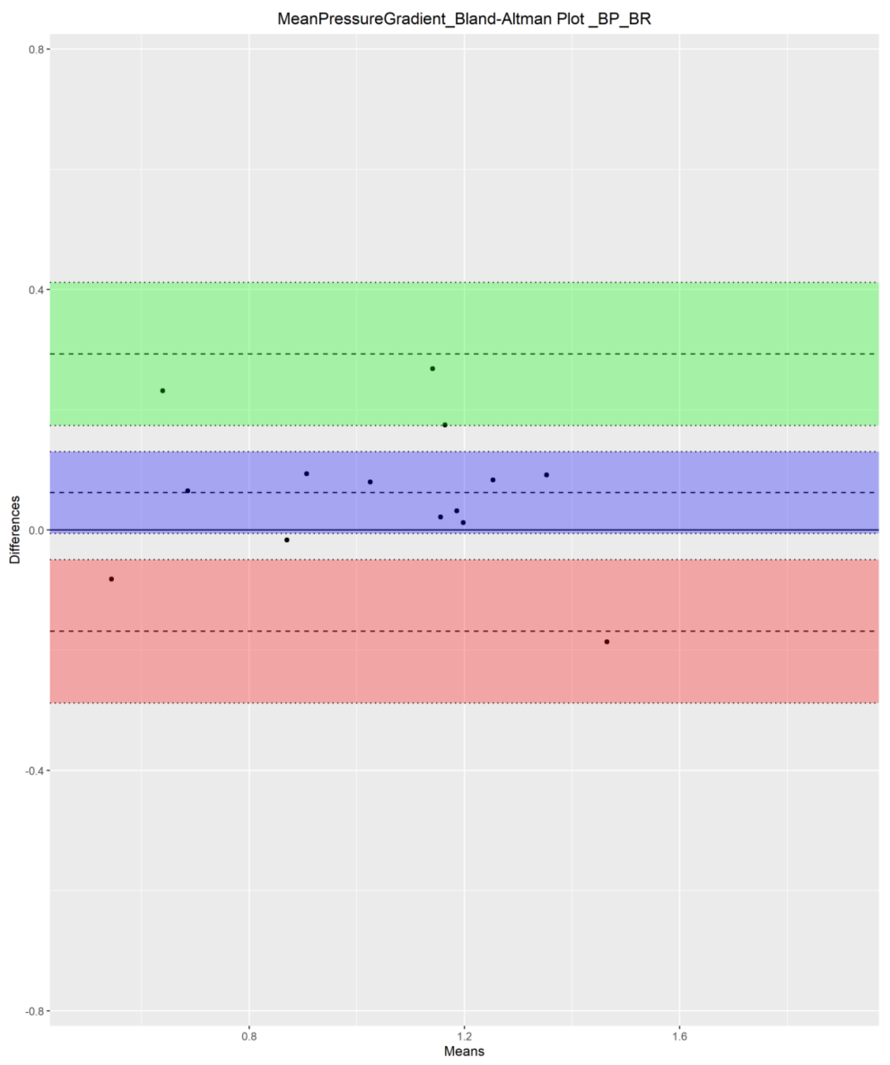

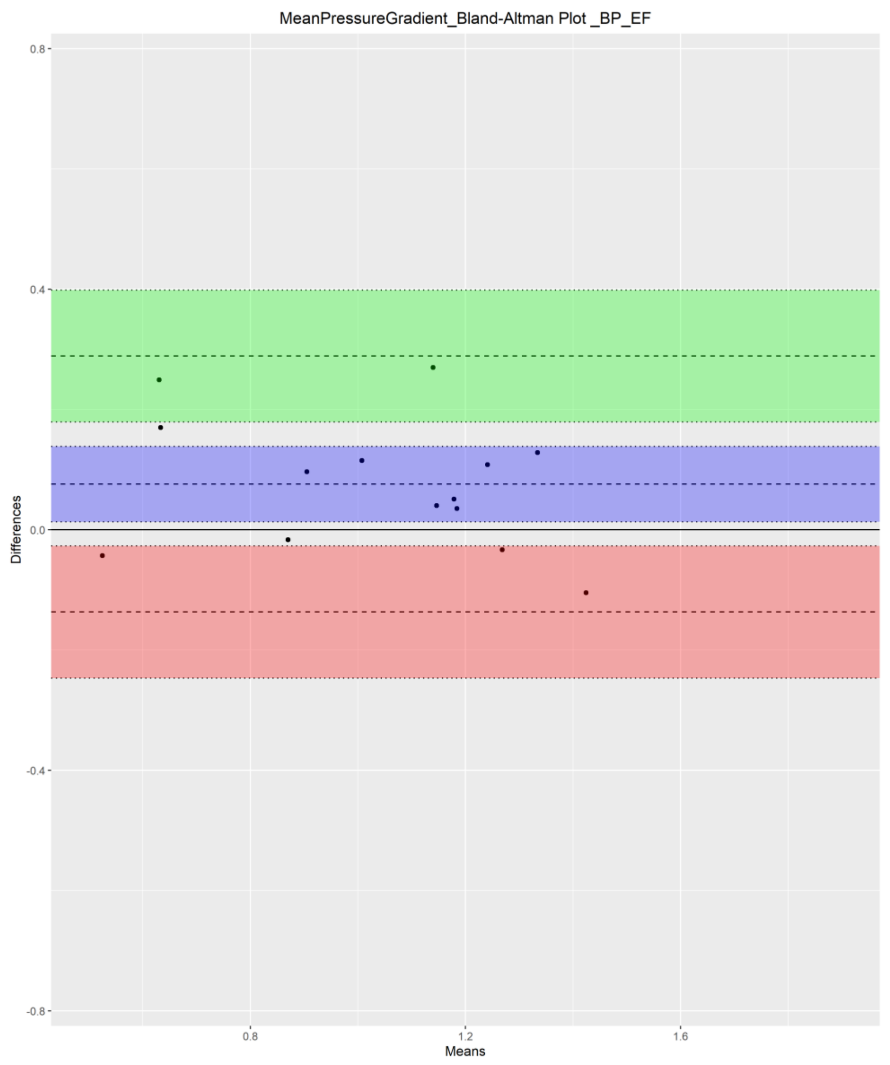

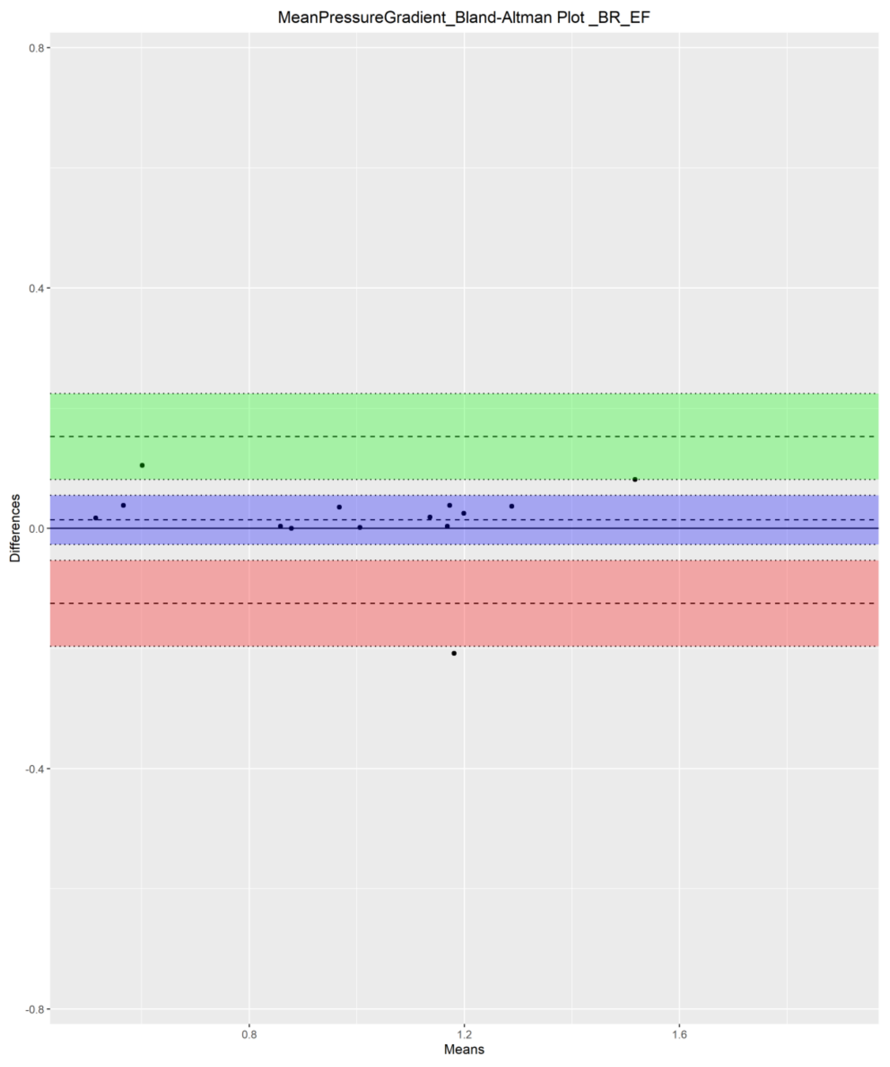

Siem3T

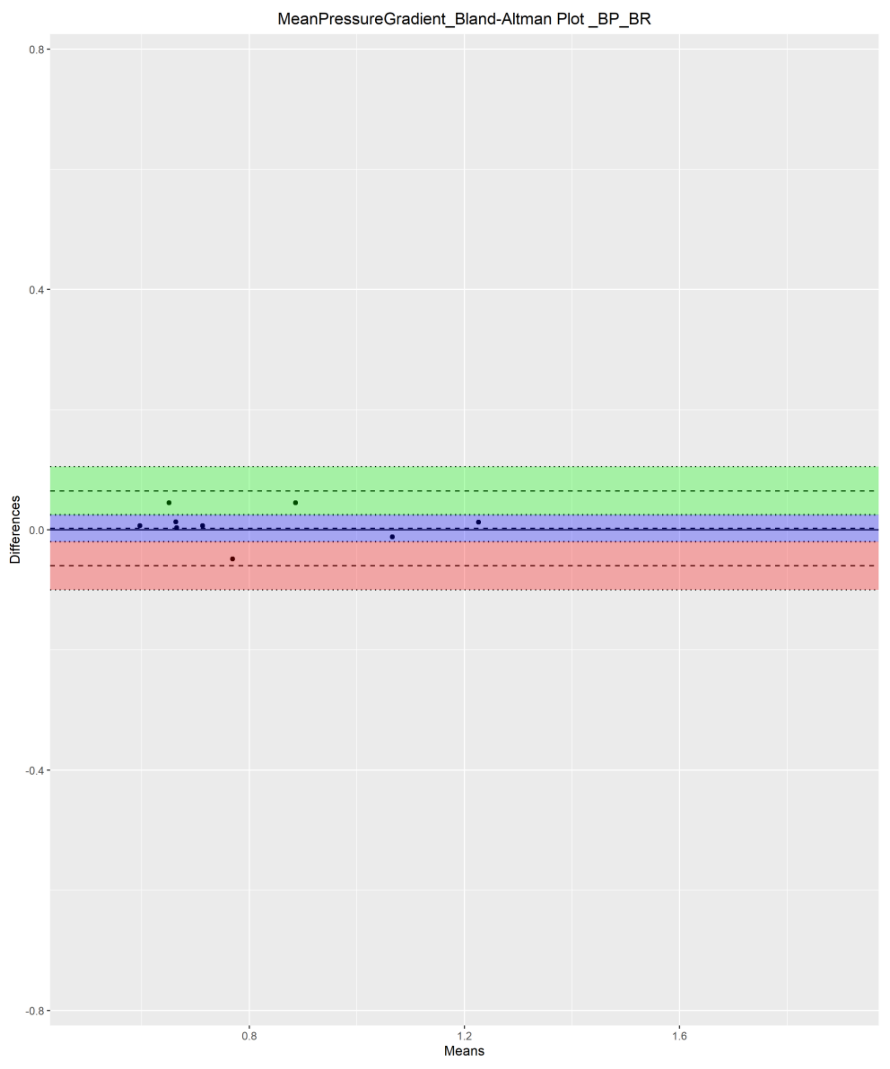

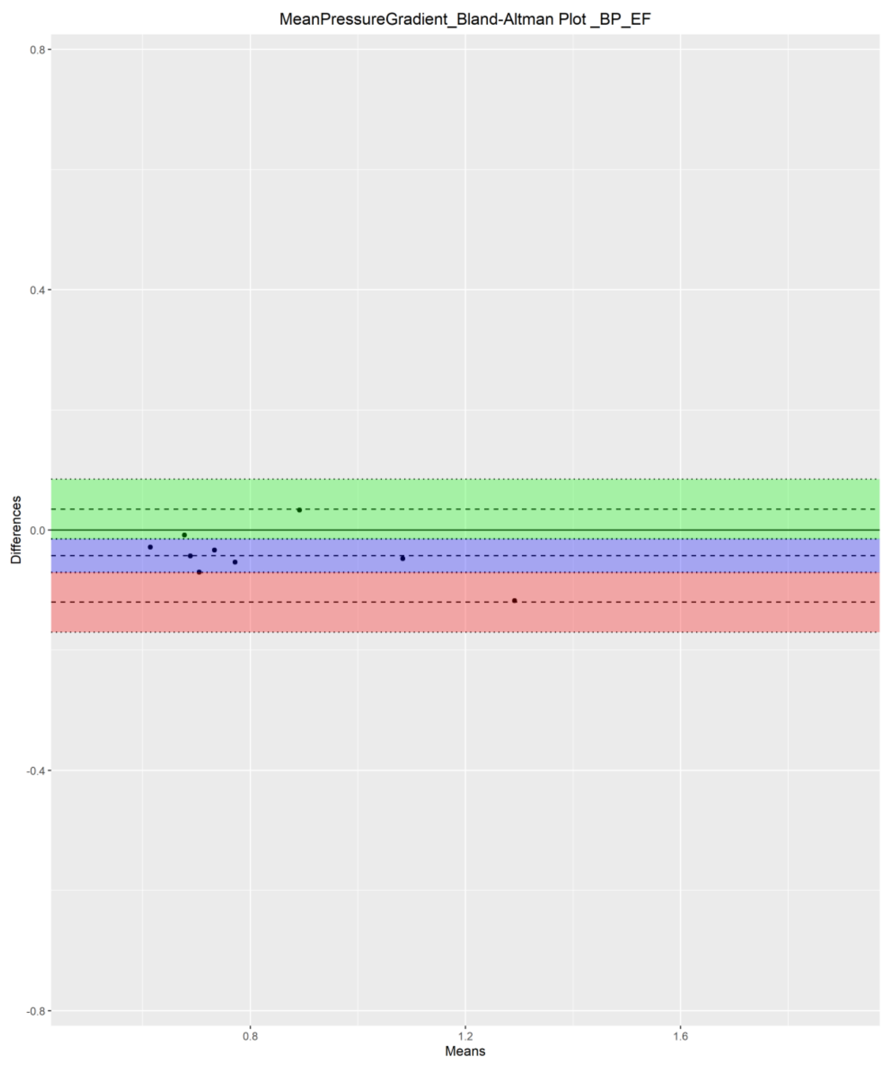

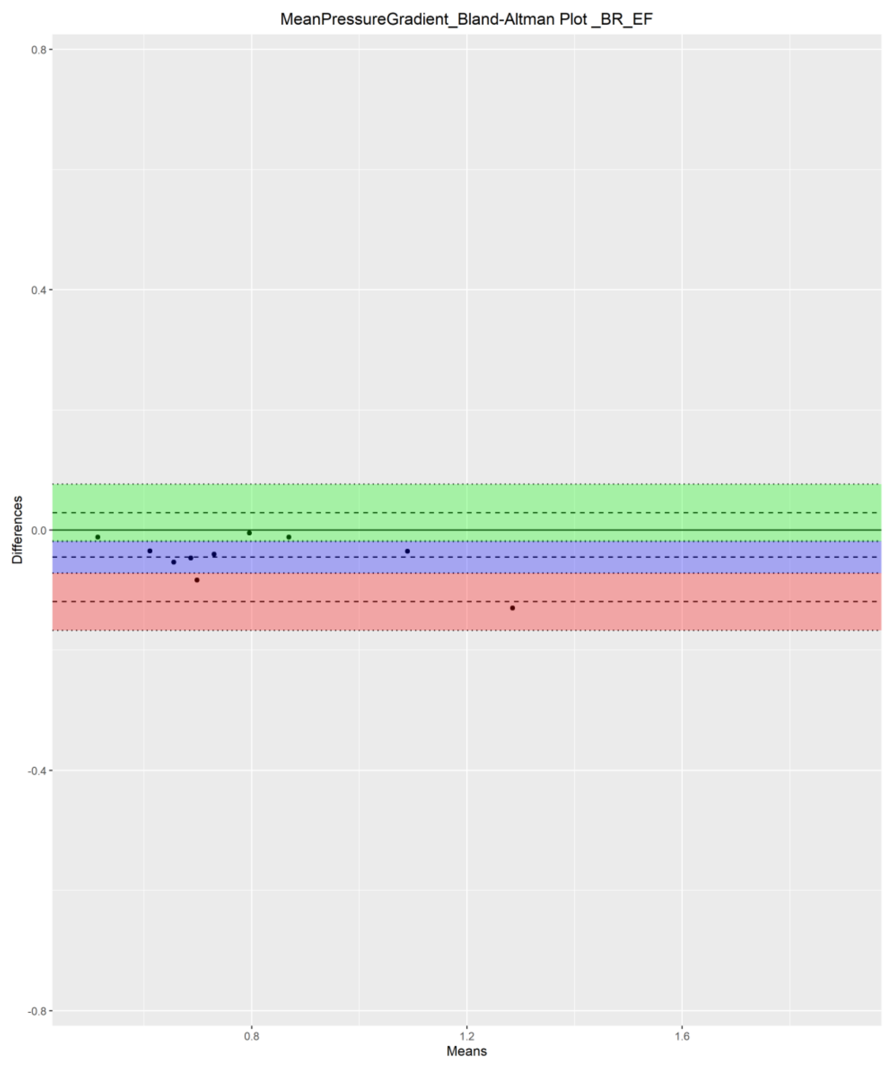

TotalVolume

Phi1.5T

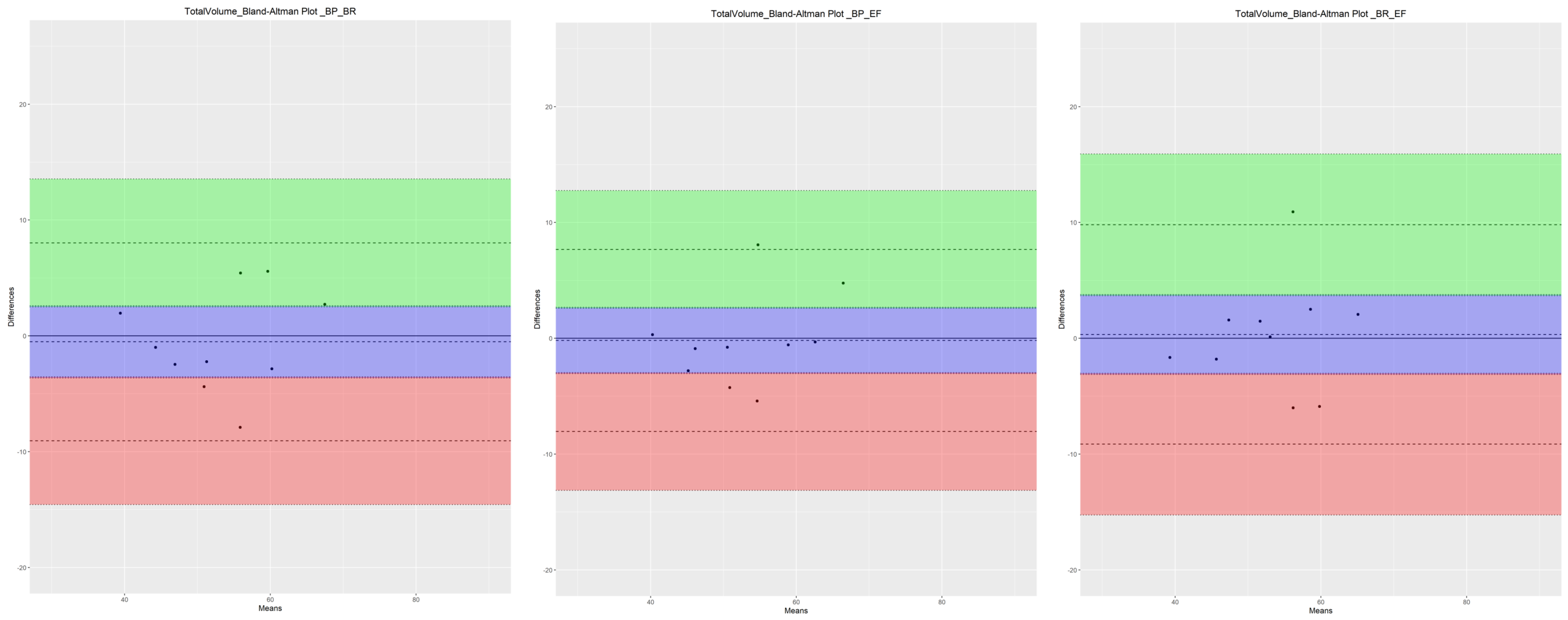

Phi3T

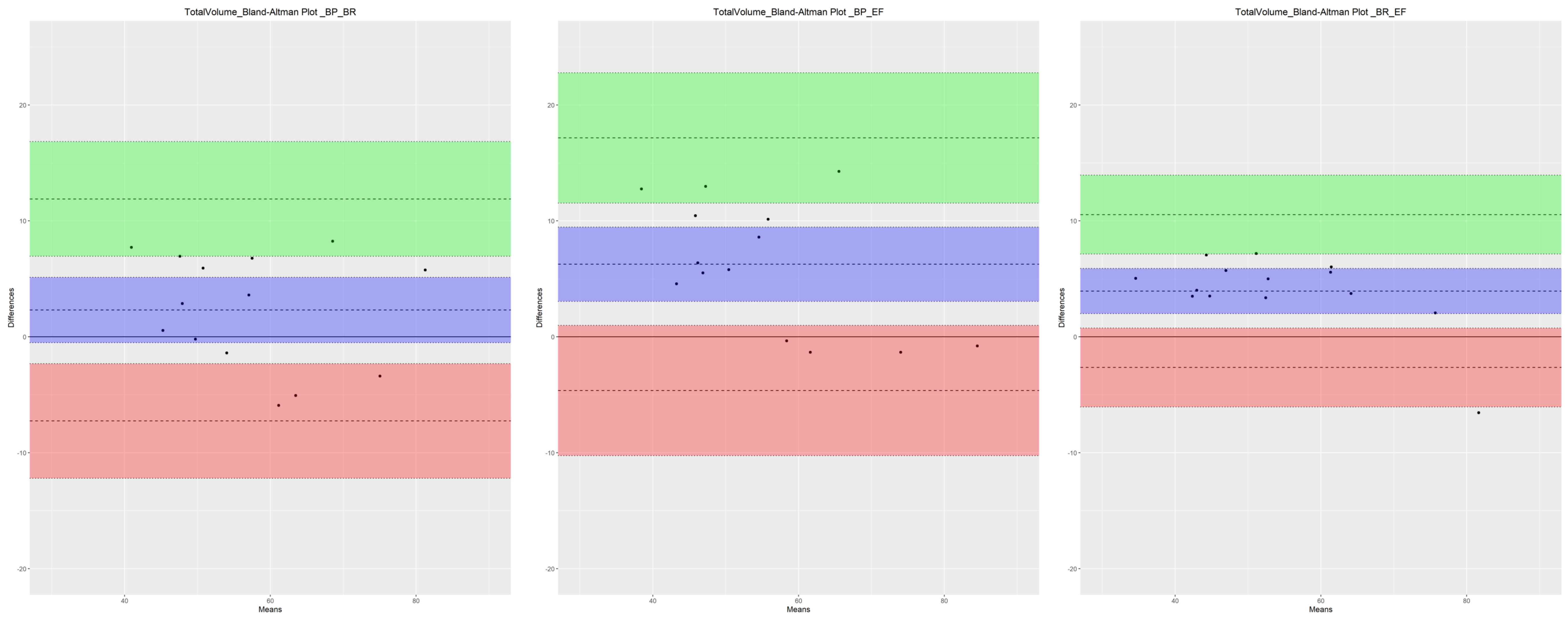

Siem3T

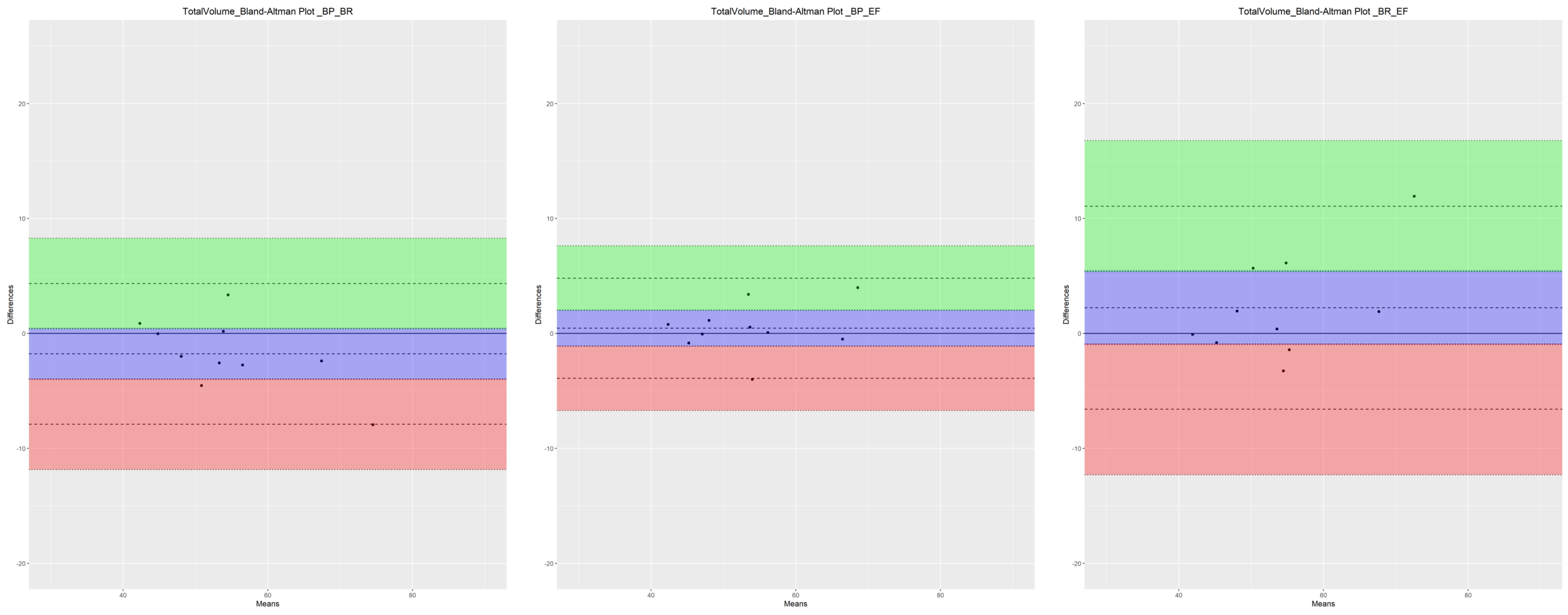

Supplement: Supplementary file 1 [file jcm-12-02960-s001.zip › Supplementary Figure S2_Bland_Altman.PDF]
