# Supplementary figures and images for: 4D-Flow Cardiovascular Magnetic Resonance Sequence for Aortic Assessment: Multi-Vendor and Multi-Magnetic Field Reproducibility in Healthy Volunteers
Source: J Clin Med. 2023 Apr 19;12(8):2960. doi: 10.3390/jcm12082960 (PMC10141060; doi:10.3390/jcm12082960)

BP\_Phi1.5T

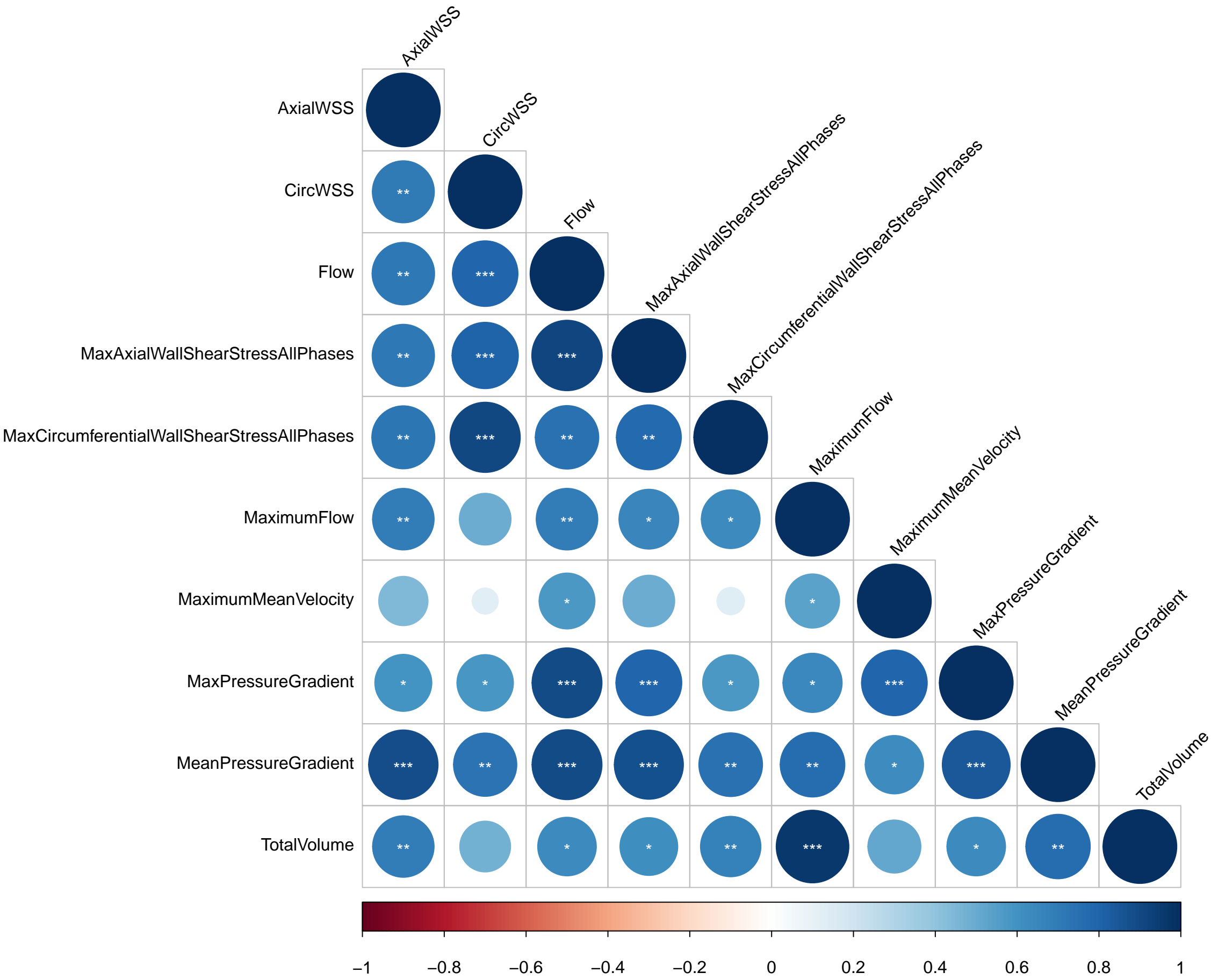

BP\_Phi3T

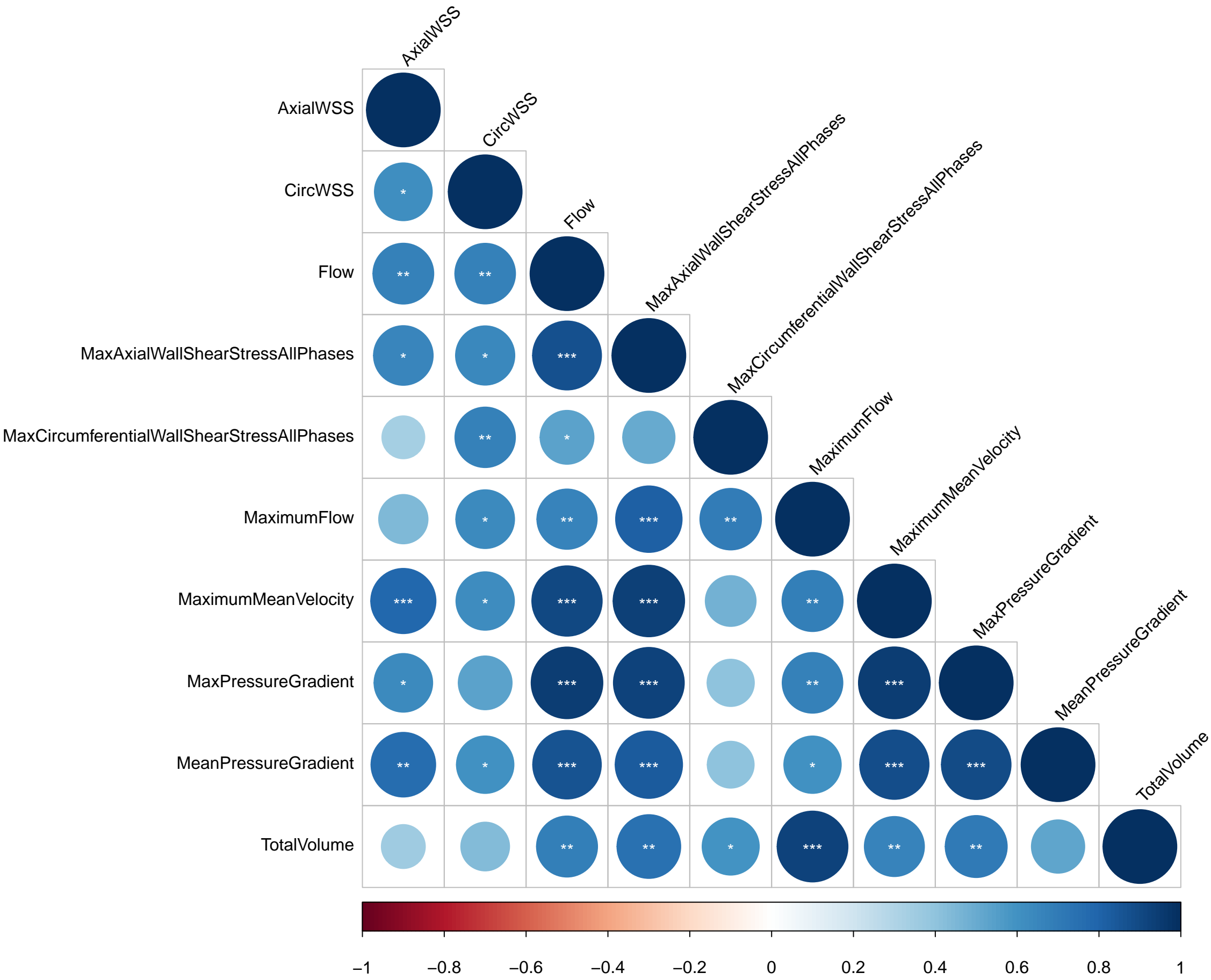

BP\_Siem3T

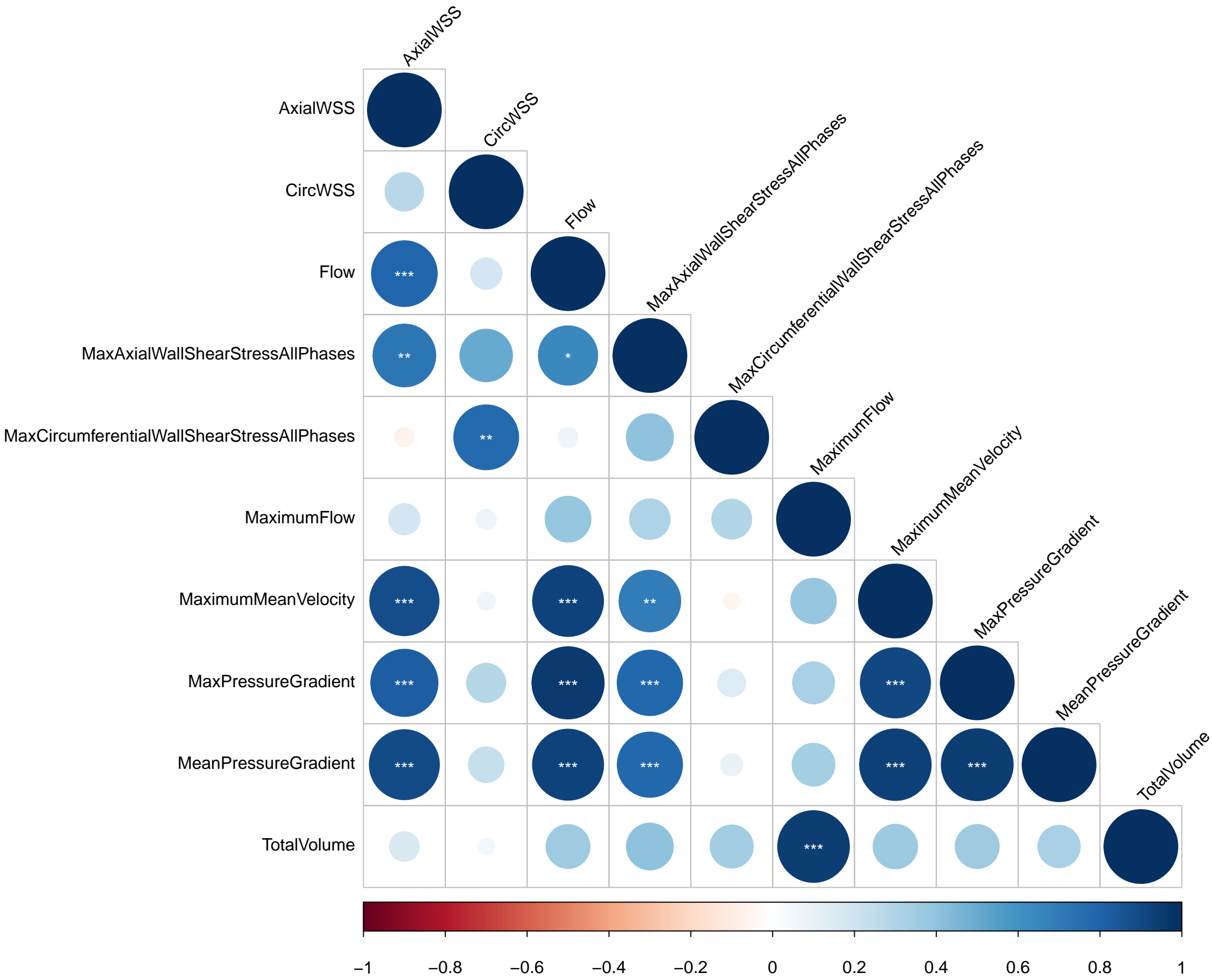

BR\_Phi1.5T

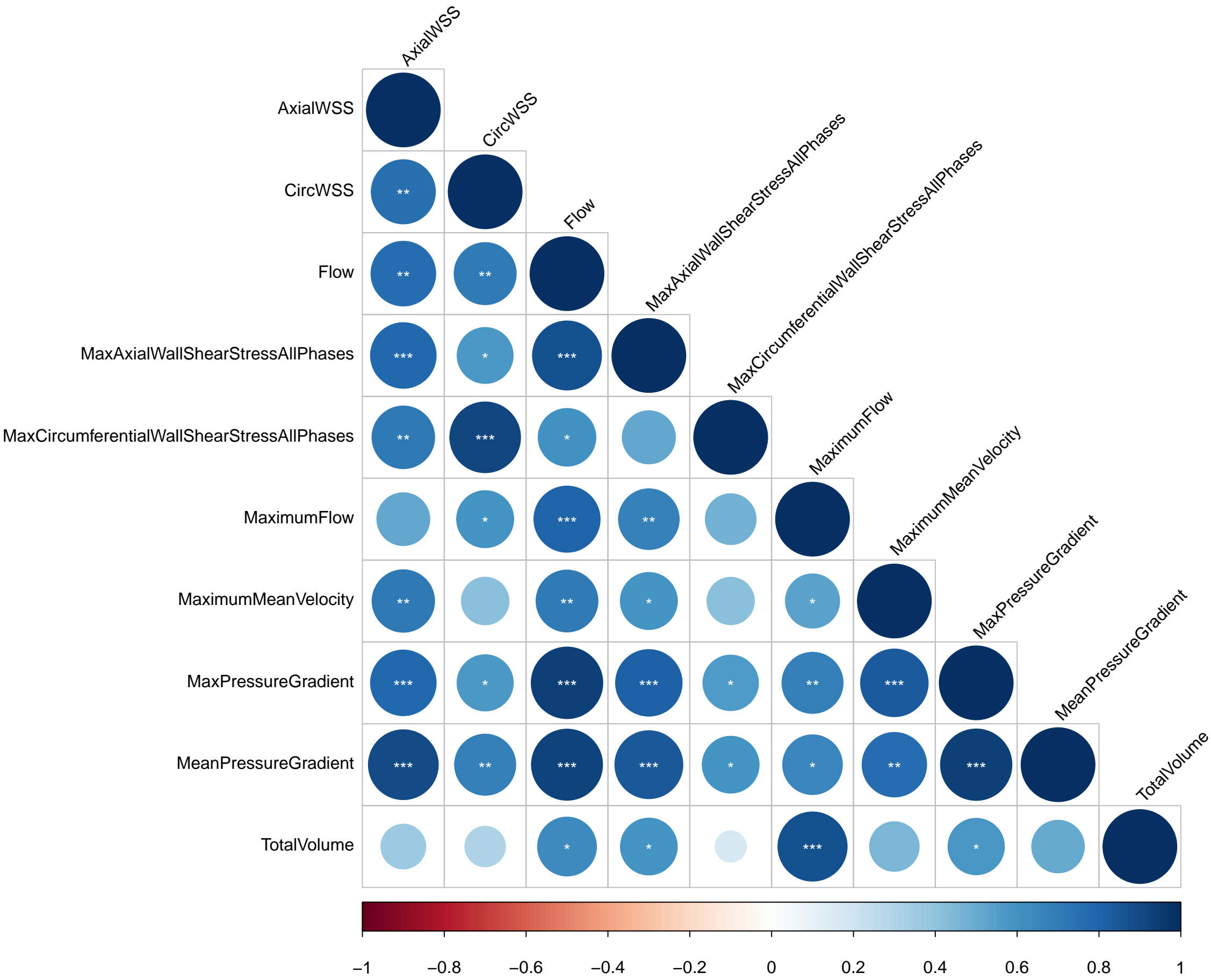

BR\_Phi3T

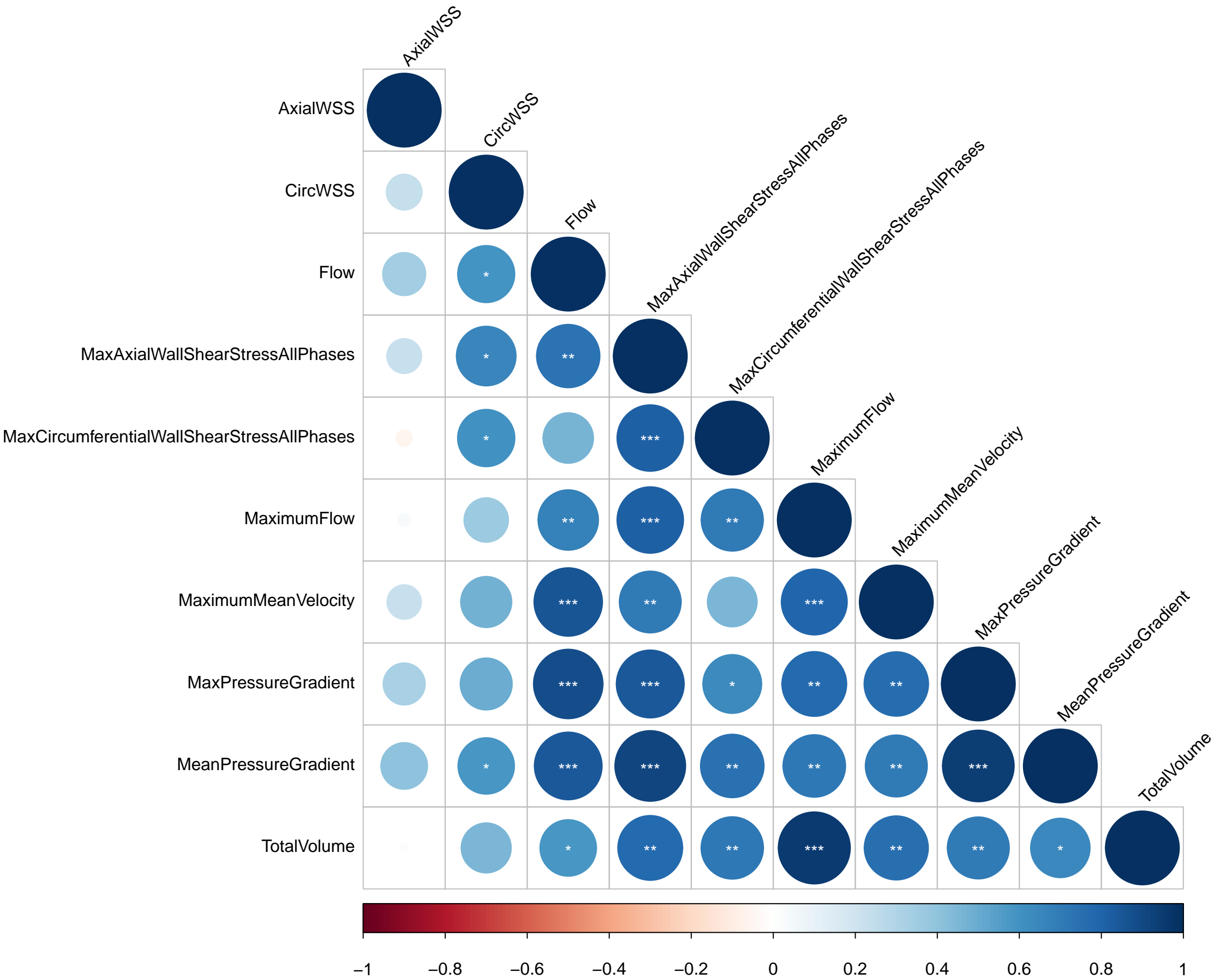

BR\_Siem3T

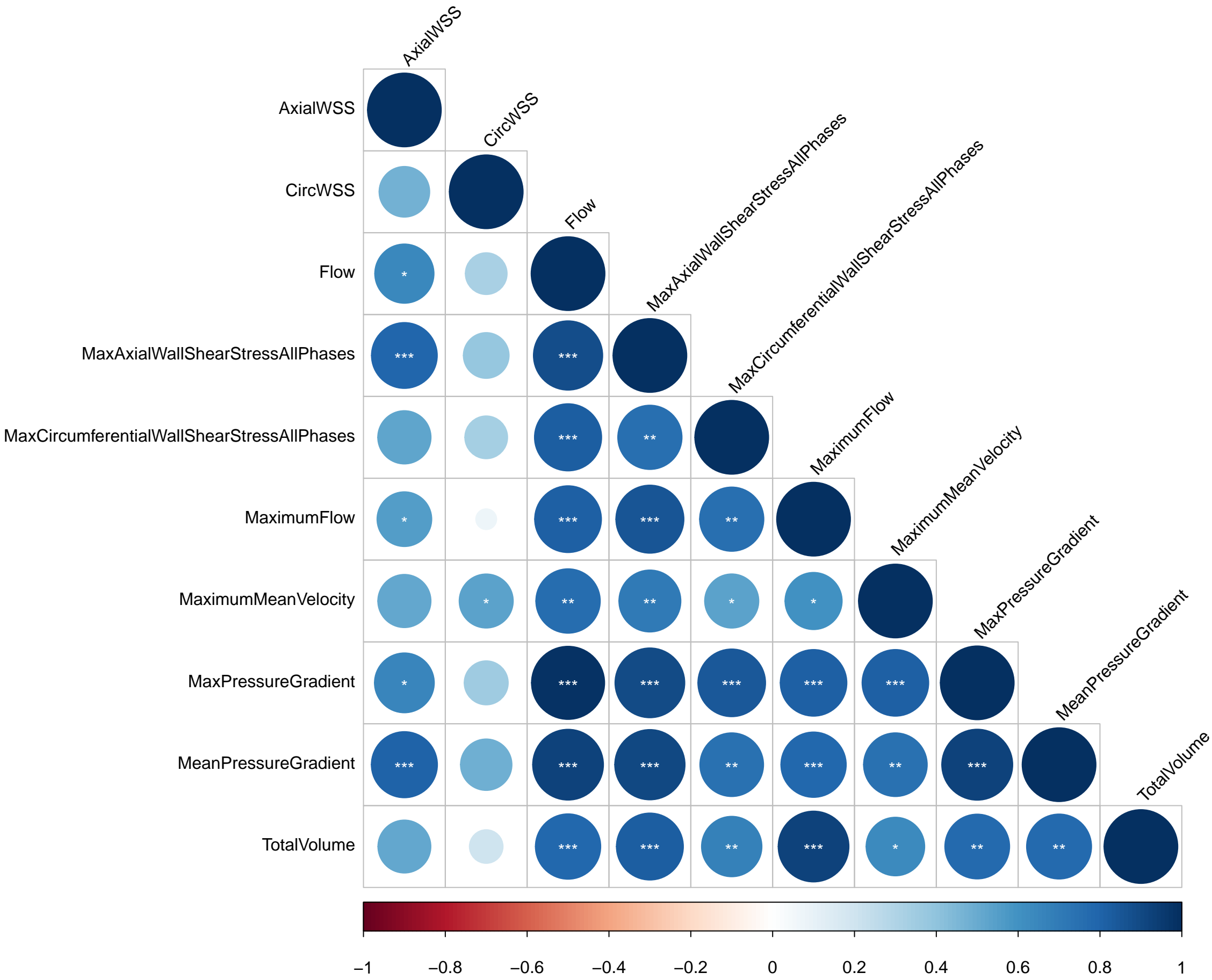

EF\_Phi1.5T

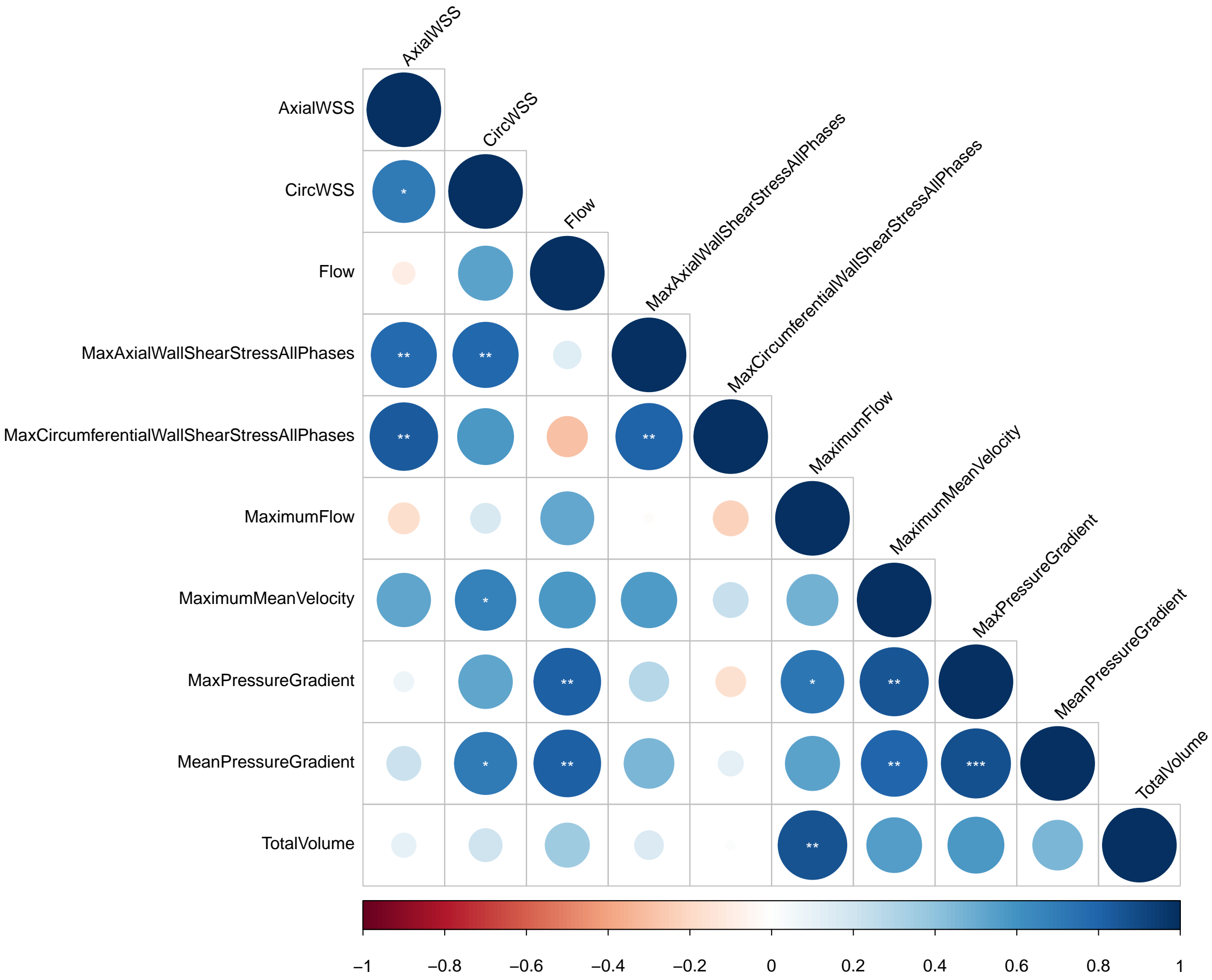

# EF\_Phi3T

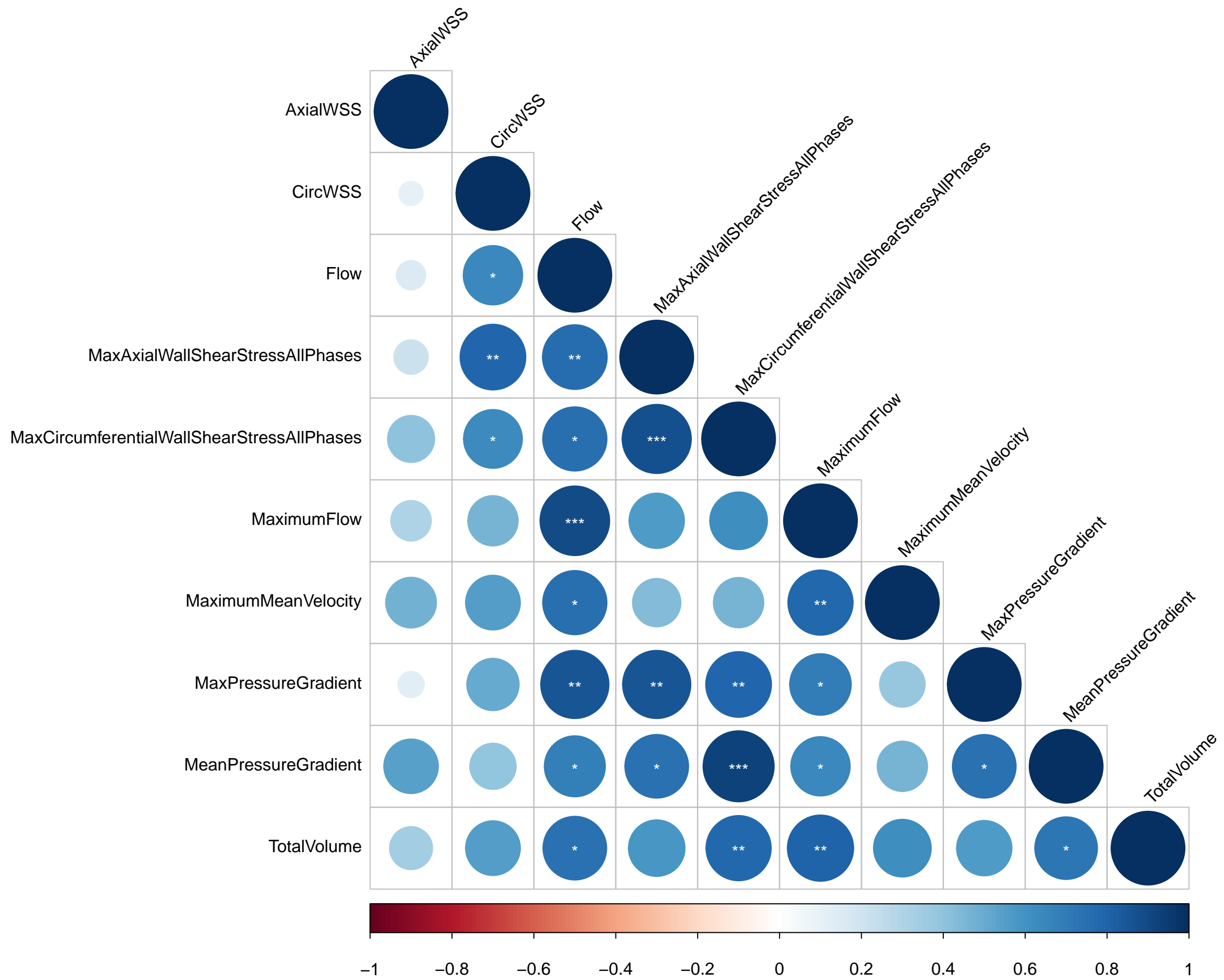

EF\_Siem3T

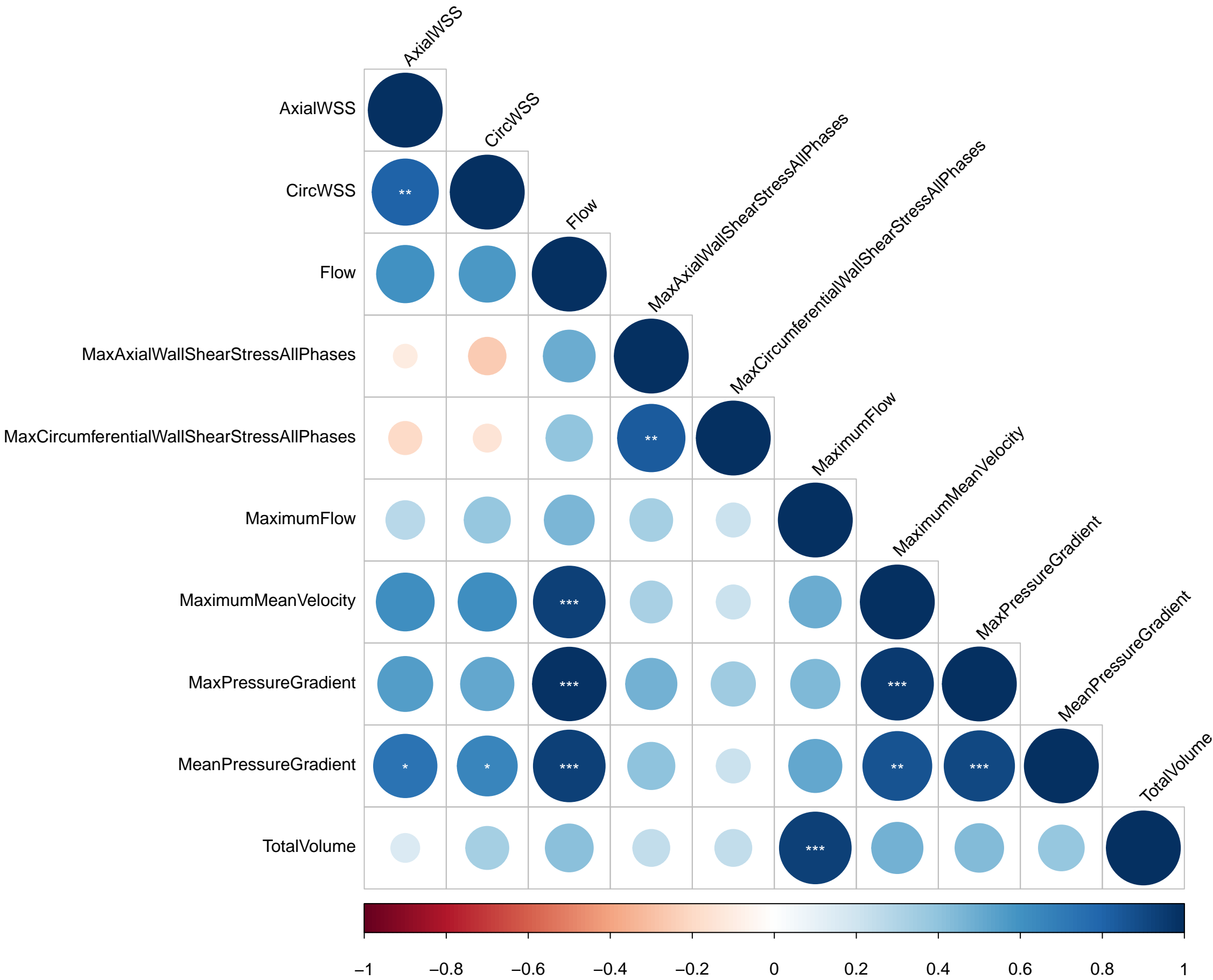

Supplement: Supplementary file 1 [file jcm-12-02960-s001.zip › Supplementary Figure S1_Correlation.PDF]
